# Preserved Cognitive Function After Statin Administration During Cancer Treatment With Doxorubicin: A Secondary Analysis of a Randomized Clinical Trial

**DOI:** 10.1001/jamanetworkopen.2025.38325

**Published:** 2025-10-21

**Authors:** Pamela J. Grizzard, Nathaniel S. O’Connell, Stephen R. Rapp, Kristine C. Olson, Moriah P. Bellissimo, Alexandria N. Hughes, Amy C. Ladd, Kathryn E. Weaver, Lynne I. Wagner, Kathryn J. Ruddy, Bonnie Ky, Ralph B. D’Agostino, W. Gregory Hundley

**Affiliations:** 1 Pauley Heart Center, Division of Cardiology, Department of Internal Medicine, Virginia Commonwealth University School of Medicine, Richmond; 2 Department of Biostatistics and Data Science, Wake Forest University School of Medicine, Winston-Salem, North Carolina; 3 Department of Psychiatry and Behavioral Medicine, Wake Forest University School of Medicine, Winston-Salem, North Carolina; 4 Department of Social Sciences and Health Policy, Wake Forest University School of Medicine, Winston-Salem, North Carolina; 5 Department of Health Policy and Management, University of North Carolina at Chapel Hill; 6 Division of Medical Oncology, Department of Oncology, Mayo Clinic, Rochester, Minnesota; 7 Department of Medicine, Perelman School of Medicine at the University of Pennsylvania, Philadelphia

## Abstract

**Importance:**

Receipt of anthracycline-based chemotherapy and statin therapy have been found to be associated with deterioration of cognitive function in patients with cancer.

**Objective:**

To assess the association of statins with attention, verbal fluency, and executive function in patients receiving doxorubicin treatment for cancer.

**Design, Setting, and Participants:**

This secondary analysis of the Preventing Anthracycline Cardiovascular Toxicity With Statins randomized clinical trial, conducted across 31 US community and academic sites from February 5, 2014, through September 24, 2020, involved participants with stage I to IV lymphoma or stage I to III breast cancer undergoing doxorubicin treatment. The data analysis was conducted between August 1, 2024, and April 4, 2025.

**Interventions:**

Participants were randomized (1:1) to receive a daily 40-mg dose of atorvastatin or placebo before initiating doxorubicin treatment and then continued for 24 months.

**Main Outcomes and Measures:**

Attention, executive function, and verbal fluency were assessed using the Trail Making Test part A (TMT-A) and part B (TMT-B) and the Controlled Oral Word Association test, respectively. Time to complete the assessments were modeled using a linear mixed model, while errors (counts) were modeled using a generalized linear mixed model assuming a Poisson distribution.

**Results:**

A total of 238 participants (mean [SD] age, 49 [12] years; 217 female [91.2%]) were randomized to the statin (n = 118) or placebo (n = 120) groups. Values for TMT-A at 6 and 24 months were similar between the statin group (mean, 32.5 seconds [95% CI, 29.4-35.7 seconds] and 29.8 seconds [95% CI, 26.4-33.2 seconds], respectively) and the placebo group (mean, 28.4 seconds [95% CI, 25.2-31.5 seconds] and 27.8 seconds [95% CI, 24.5-31.0 seconds], respectively). From before treatment to 24 months after treatment, statin recipients had a significant mean improvement of 10.2 seconds for TMT-B (95% CI, 1.9-18.5 seconds), whereas placebo recipients had a nonsignificant improvement of only 0.2 seconds for TMT-B (95% CI, −8.5 to 8.1 seconds). Analysis of the time-by-treatment interaction showed no significant difference between groups. For Controlled Oral Word Association scores in the same period, both the placebo and statin groups showed a mean improvement of 3.62 points (95% CI, 1.71-5.54 points) and 4.74 points (95% CI, 2.69-6.79 points), respectively, with no difference between groups.

**Conclusions and Relevance:**

This secondary analysis of a randomized clinical trial suggests that the receipt of statins during doxorubicin therapy for lymphoma or breast cancer was not associated with worsening cognitive functions over 24 months. Within-group analyses suggested that statins may have contributed to better executive function on the TMT-B.

**Trial Registration:**

ClinicalTrials.gov Identifier: NCT01988571

## Key Points

**Question:**

What is the association of atorvastatin with cognitive function in patients receiving doxorubicin for cancer treatment over 24 months?

**Findings:**

In this secondary analysis involving 238 patients from the Preventing Anthracycline Cardiovascular Toxicity With Statins randomized clinical trial, there was a significant improvement of 10.2 seconds in time to complete the Trail Making Test part B within the statin group compared with only 0.2 seconds in the placebo group; however, between-group differences were not significant. Scores for 2 additional tests of cognitive function were similar between the groups.

**Meaning:**

These findings suggest that participants who receive statins during anthracycline-based cancer treatment may not experience deterioration in cognitive function.

## Teaser

This secondary analysis of the Preventing Anthracycline Cardiovascular Toxicity With Statins trial assessed the association of statins with cognitive function in patients receiving doxorubicin treatment for cancer.

## Introduction

Statins (3-hydroxy-3-methylglutaryl coenzyme A reductase inhibitors)^1^ are prescribed to an estimated 35 million patients in the US and have been proven effective in lowering low-density lipoprotein (LDL) cholesterol and the occurrence of cardiovascular events, including myocardial infarction and stroke.^2,3^ Statin-related cognitive adverse effects have been a topic of interest among patients and health care professionals since 2012, when a US Food and Drug Administration safety announcement was published.^4,5^ Cognitive difficulties, including executive function, a central cognitive process involving regulation of complex behaviors, planning, and cognitive flexibility,^6,7^ also have occurred in up to 35% of patients undergoing cancer treatment.^8,9^

Recently, due to their pleiotropic antioxidative and anti-inflammatory properties, statins have been studied in the prevention of left ventricular myocardial injury in patients receiving doxorubicin for breast cancer and lymphoma.^10,11,12^ However, at this time, it is unknown whether the administration of statins promotes deterioration of cognitive functions, such as memory, language, attention, and executive function, in patients receiving doxorubicin for treatment of lymphoma or breast cancer.

Accordingly, this study examined the association between statins and cognition in clinical trial patients receiving doxorubicin for breast cancer or lymphoma. Given the prior evidence and considering our study population, we hypothesized greater deterioration of cognitive function in patients randomized to atorvastatin vs placebo 24 months after initiating doxorubicin treatment.

## Methods

### Study Design and Population

This preplanned secondary analysis of a multicenter, double-blind, placebo-controlled, randomized clinical trial (RCT), the Preventing Anthracycline Cardiovascular Toxicity With Statins (PREVENT) study, was performed through the Wake Forest National Cancer Institute Community Oncology Research Program Research Base, Eastern Cooperative Oncology Group–American College of Radiology Imaging Network, and Alliance for Clinical Trials in Oncology networks.^11^ The trial protocol (Supplement 1) and informed consent form were approved by the Wake Forest University School of Medicine Institutional Review Board. All participants provided written informed consent and were aged 21 years or older with an expected survival of more than 2 years. The trial followed the Consolidated Standards of Reporting Trials (CONSORT) reporting guideline.

The primary RCT randomly assigned (1:1) 279 adults enrolled across 31 community and academic sites between February 4, 2014, and September 24, 2020, to receive a daily single 40-mg dose of atorvastatin or placebo prior to initiation of doxorubicin for stage I to III breast cancer or stage IV lymphoma and for at least 24 months (eFigure 1 in Supplement 2). Participant compliance with taking the study agent was monitored through diaries and returned pill checks at 6 and 24 months. Cognitive function, including memory, attention, verbal fluency, and executive function, were predefined secondary outcomes.^11^

### Neurocognitive Measures and Outcome

The trial included a battery of validated neurocognitive measures recommended for studies of patients with cancer.^13^ Included were assessments of verbal learning and memory (Hopkins Verbal Learning Test–Revised),^14^ working memory (Digit Span Test), verbal fluency (Controlled Oral Word Association [COWA] test), attention (Trail Making Test part A [TMT-A]), and executive function (Trail Making Test part B [TMT-B]). The PREVENT investigators previously reported that patients receiving chemotherapy plus statin did not experience a worsening of verbal learning and memory (Hopkins Verbal Learning Test–Revised)^15^ compared with those receiving placebo.^11^ This preplanned secondary analysis focused on attention, verbal fluency, and executive function, as these measures have been identified as common deficits experienced in breast cancer survivors.^16^ Neurocognitive testing was completed before treatment (ie, baseline) and then 6 and 24 months after initiating doxorubicin. A decision was made after enrolling only 24 participants to remove the Digit Span Test to reduce participant burden and enhance accrual; thus, these data were not analyzed.

For TMT-A, the respondent is instructed to connect 25 numbered circles organized in a random pattern on a sheet of paper in proper sequence (eg, 1-2-3 and so forth) as quickly as possible. For TMT-B with its added complexity and set-shifting requirements, the respondent is instructed to connect 25 randomly ordered circles, half of which are numbered (1-13) and half of which are lettered (A-L) in alternating sequence (eg, 1-A-2-B and so forth) as quickly as possible. The primary score for either the TMT-A or TMT-B is the total time in seconds required to complete each task. A lower time to complete and lower error count correspond to better performance.^17^

The COWA test is used to assess verbal fluency and executive function.^18,19^ Respondents are asked to name as many words as possible, all beginning with a specified letter, in 1 minute. Three trials are administered and summed, with higher scores reflecting better verbal fluency. The 2 versions (version A, which uses letters *C*, *F*, and *L*, and version B, which uses *P*, *R*, and *W*) were administered alternately to reduce practice effects.^20^ Scores are calculated by totaling the number of acceptable words produced for all 3 letters minus repetition.^19^ The main objective of this secondary analysis was to evaluate whether chemotherapy plus statin is associated with worsened cognitive functions of attention, verbal fluency, or executive function compared with chemotherapy alone during the 24-month study period.

### Statistical Analysis

Patient characteristics and biomarkers were summarized using mean, SD, median, and minimum to maximum for continuous variables and frequency and percentages for categorical variables. Our analyses of time to complete TMT-A and TMT-B (in seconds) and COWA scores from baseline to 24 months were modeled using linear mixed models, while errors (counts) were modeled in a generalized linear mixed model. A Pearson χ^2^ test was first used to test for overdispersion of the count data, and due to its absence, we pursued a Poisson model with log link function over a negative binomial model. In each model, time was considered as a discrete estimator (pretreatment [baseline], 6 months, and 24 months), along with treatment group (statin vs placebo), random intercepts for each patient, relevant patient characteristics, and clinical biomarkers included as confounding variables. Confounding variables considered in the initial model were age, education, cancer type (lymphoma vs breast), income, job status, self-identified race (Black, White, or other [American Indian or Alaska Native, Asian, Native Hawaiian or Pacific Islander, or unknown]), self-identified ethnicity (Hispanic/Latino, not Hispanic/Latino, or unknown), systolic blood pressure, diastolic blood pressure, left ventricular ejection fraction, cholesterol, LDL, high-density lipoprotein (HDL), glucose, troponin I, and inflammatory markers^21^ (C-reactive protein, interleukin 6, and tumor necrosis factor α). The inclusion of race and ethnicity in our statistical models was done to help reduce variability and increase precision of the effects being estimated; to ensure that the models were tractable and results reliable, we collapsed all races other than Black and White into a single other category.

For covariate selection, imputation by random forest using the R package missForest, version 1.5^22,23^ was used to impute missing values, then least absolute shrinkage and selection operator regression using the R package glmmLasso, version 1.6.3 with the tuning parameter λ was chosen based on the bayesian information criterion.^24^ From this least absolute shrinkage and selection operator model, the variables selected were used in a complete case analysis in linear mixed models. Interaction effects were included to assess significant changes over time by each treatment group; these are type III sums of squares tests of fixed effects that, for simplicity, are described as interaction effects throughout. Interaction effects were included in the models to assess the primary hypothesis for each analysis (is there a differential change over time in the outcome by treatment group?). Changes over time were interpreted within groups based on this included interaction effect, regardless of its *P* value. However, statistical significance between groups or changes over time were assessed independently at an α of .05 without controlling for multiple testing. All analyses were conducted between August 1, 2024, and April 4, 2025, using RStudio, version 4.4.1 (Posit Software, PBC).

## Results

### Sample Characteristics

We included 238 participants (mean [SD] age, 49 [12] years; 217 female [91.2%] and 21 male [8.8%]; 30 identifying as Black [12.6%], 201 as White [84.5%], and 7 as other [2.9%] race; 5 identifying as Hispanic/Latino [2.1%], 232 as not Hispanic/Latino [97.5%], and 1 as unknown [0.4%] ethnicity) from the overall 279 with neurocognitive outcome measures at multiple time points. There were 120 participants (50.4%) in the placebo group and 118 (49.6%) in the statin group; 202 (84.9%) were diagnosed with breast cancer and 36 (15.1%) with lymphoma. The mean (SD) body mass index (calculated as weight in kilograms divided by height in meters squared) for the cohort was within the overweight category (29.9 [7.1]). The cohort was predominately married (156 participants [65.5%]), employed (159 participants [66.8%]), and had a college degree (138 participants [58.0%]). Further details of participant demographics by group are shown in Table 1. Characteristics of participants with and without cognitive outcome data are shown in eTables 1 and 2 in Supplement 2. We observed that the characteristics of participants with and without cognitive outcome data were similar in the overall study and the analyzed population. Table 2 presents clinical markers and biomarker data by treatment group and time point for lipid levels, cardiac function, glucose concentrations, and inflammation.

**Table 1.** Baseline Participant Demographics

Characteristic	Participants, No. (%)		
	Placebo group (n = 120)	Statin group (n = 118)	Overall cohort (N = 238)
Height, mean (SD), cm	167 (8.6)	166 (7.4)	166 (8.0)
Weight, mean (SD), kg	86.0 (20.7)	79.2 (19.6)	82.6 (20.4)
Body mass index, mean (SD)^a^	31.0 (7.5)	28.7 (6.6)	29.9 (7.1)
Age, mean (SD), y	49.2 (11.6)	48.9 (12.2)	49.1 (11.9)
Sex			
Female	108 (90.0)	109 (92.4)	217 (91.2)
Male	12 (10.0)	9 (7.6)	21 (8.8)
Race			
Black	18 (15.0)	12 (10.2)	30 (12.6)
White	101 (84.2)	100 (84.7)	201 (84.5)
Other^b^	1 (0.8)	6 (5.1)	7 (2.9)
Ethnicity			
Hispanic/Latino	3 (2.5)	2 (1.7)	5 (2.1)
Not Hispanic/Latino	117 (97.5)	115 (97.5)	232 (97.5)
Unknown	0	1 (0.8)	1 (0.4)
Cancer type			
Breast	102 (85.0)	100 (84.7)	202 (84.9)
Lymphoma	18 (15.0)	18 (15.3)	36 (15.1)
Cancer stage			
I	17 (14.2)	20 (16.9)	37 (15.5)
II	68 (56.7)	65 (55.1)	133 (55.9)
III	34 (28.3)	29 (24.6)	63 (26.5)
IV	1 (0.8)	4 (3.4)	5 (2.1)
Marital status			
Married	80 (66.7)	76 (64.4)	156 (65.5)
Other^c^	40 (33.3)	42 (35.6)	82 (34.5)
Education			
College	77 (64.2)	61 (51.7)	138 (58.0)
Graduate or professional	24 (20.0)	29 (24.6)	53 (22.3)
High school	19 (15.8)	28 (23.7)	47 (19.7)
Income, $			
<35 000	32 (26.7)	30 (25.4)	62 (26.1)
35 000-75 000	37 (30.8)	39 (33.1)	76 (31.9)
>75 000	51 (42.5)	49 (41.5)	100 (42.0)
Job status			
Disabled	6 (5.0)	3 (2.5)	9 (3.8)
Employed	84 (70.0)	75 (63.6)	159 (66.8)
Retired	12 (10.0)	13 (11.0)	25 (10.5)
Other^d^	18 (15.0)	27 (22.9)	45 (18.9)
TMT-A time			
Mean (SD) time, s	28.1 (11.0)	32.1 (15.4)	30.1 (13.5)
Missing	0	0	0
TMT-B, time			
Mean (SD) time, s	69.0 (39.3)	74.1 (42.7)	71.6 (41.0)
Missing	9 (7.5)	3 (2.5)	12 (5.0)
TMT-B − TMT-A			
Time, mean (SD), s	40.6 (34.6)	41.9 (34.7)	41.3 (34.6)
Missing	9 (7.5)	3 (2.5)	12 (5.0)
TMT-A errors			
Mean (SD) No.	0.24 (0.5)	0.31 (0.6)	0.27 (0.6)
Missing	1 (0.8)	0	1 (0.4)
TMT-B errors			
Mean (SD) No.	0.4 (0.9)	0.5 (1.0)	0.5 (1.0)
Missing	10 (8.3)	3 (2.5)	13 (5.5)
COWA score			
Mean (SD) points	40.72 (11.83)	37.08 (10.82)	38.91 (11.47)
Missing	1 (0.8)	0 (0)	1 (0.4)

Abbreviations: COWA, Controlled Oral Word Association Test; TMT-A, Trail Making Test part A; TMT-B, Trail Making Test part B.

^a^ Calculated as weight in kilograms divided by height in meters squared.

^b^ Included American Indian or Alaska Native, Asian, Native Hawaiian or Pacific Islander, or unknown.

^c^ Included single never married, living in a married-like relationship, separated or divorced, widowed, or prefer not to answer.

^d^ Included not working, homemaker, and unknown.

**Table 2.** Participant Health and Biomarker Data

Variable	Baseline, mean (SD)		6 mo, Mean (SD)		24 mo, Mean (SD)	
	Placebo (n = 120)	Statin (n = 118)	Placebo (n = 120)	Statin (n = 118)	Placebo (n = 120)	Statin (n = 118)
SBP, mm Hg	129 (18.3)	125 (17.1)	125 (16.7)	121 (16.6)	127 (17.1)	124 (16.1)
DBP, mm Hg	78.1 (12.7)	76.0 (10.7)	76.7 (15.7)	73.9 (9.50)	75.9 (10.7)	74.9 (12.1)
LVEF, %	61.8 (5.37)	62.6 (6.52)	57.5 (6.45)	57.5 (6.28)	57.4 (6.87)	57.7 (5.59)
Cholesterol, mg/dL^a^	193 (32.8)	195 (41.6)	200 (30.9)	156 (37.4)	196 (36.3)	172 (41.9)
LDL cholesterol, mg/dL^a^	112 (27.4)	113 (29.3)	116 (29.1)	75.2 (31.7)	110 (34.0)	89.6 (37.4)
HDL cholesterol, mg/dL^a^	56.6 (14.0)	59.9 (18.1)	55.4 (15.9)	59.3 (17.2)	60.6 (17.1)	58.7 (16.1)
Glucose, mg/dL^b^	91.3 (10.3)	92.2 (15.1)	99.2 (26.1)	95.6 (17.0)	97.0 (17.8)	96.9 (19.1)
Troponin I, ng/mL^c^	0.01 (0.01)	0.02 (0.08)	0.03 (0.11)	0.03 (0.07)	0.02 (0.11)	0.01 (0.010)
CRP, mg/dL^d^	8.80 (17.1)	7.38 (21.2)	4.93 (8.04)	3.79 (14.0)	3.72 (3.85)	4.53 (9.76)
IL-6, pg/mL	4.94 (9.71)	5.66 (10.1)	3.45 (4.79)	3.19 (3.86)	3.36 (3.24)	3.90 (7.79)
TNF-α, pg/mL	1.76 (2.46)	1.51 (1.36)	1.24 (0.52)	1.09 (0.50)	1.20 (0.48)	1.23 (0.59)

Abbreviations: CRP, C-reactive protein; DBP, diastolic blood pressure; HDL, high-density lipoprotein; IL-6, interleukin 6; LDL, low-density lipoprotein; LVEF, left ventricular ejection fraction; SBP, systolic blood pressure; TNF-α, tumor necrosis factor α.

^a^ To convert to mmol/L, multiply by 0.0259.

^b^ To convert to mmol/L, multiply by 0.0555.

^c^ To convert to g/L, multiply by 1.

^d^ To convert to mg/L, multiply by 10.

### Cognitive Function

**Figure 1.**
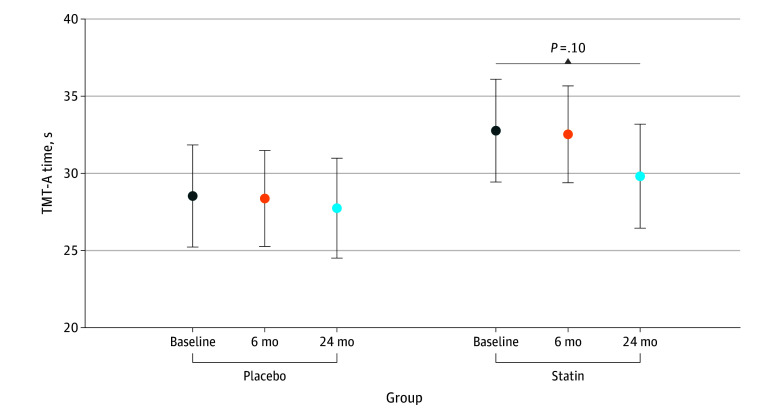
Time to Complete the Trail Making Test Part A (TMT-A) Error bars indicate the 95% CI estimated from the longitudinal linear mixed model shown in eTable 4 in Supplement 2.

Mean scores for the 3 cognitive measures at each time point are presented by group in eTable 3 in Supplement 2. In both the placebo and statin groups, scores for time to completion of TMT-A and TMT-B and COWA scores improved from baseline to 24 months. In multivariable models (adjusted for age, education, cancer type, income, job status, race and ethnicity, systolic blood pressure, diastolic blood pressure, left ventricular ejection fraction, cholesterol, LDL, HDL, glucose, troponin I, and inflammatory markers) for TMT-A time to completion (eTable 4 in Supplement 2; Figure 1) and error count (eTable 5 and eFigure 2 in Supplement 2), no statistically significant differences were found by treatment group over time. Mean time to completion in the statin group was slightly slower than the placebo group at baseline (32.8 seconds [95% CI, 29.4-36.1 seconds] and 28.5 seconds [95% CI, 25.2-31.9 seconds], respectively), but the change over time within the statin group at 6 months and 24 months (mean, 32.5 seconds [95% CI, 29.4-35.7 seconds] and 29.8 seconds [95% CI, 26.4-33.2 seconds], respectively) did not significantly differ from that of the placebo group (mean, 28.4 seconds [95% CI, 25.2-31.5 seconds] and 27.8 seconds [95% CI, 24.5-31.0 seconds], respectively) (interaction *P* = .59). From baseline to 24 months, we did not find a statistically significant change in time to completion of TMT-A in the placebo and statin groups. Similarly for the error count, the change over time was similar, but not significant between the statin and placebo groups.

**Figure 2.**
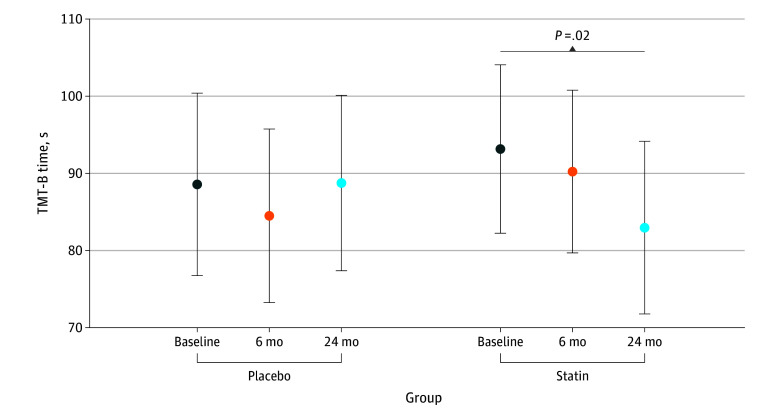
Time to Complete the Trail Making Test Part B (TMT-B) Error bars indicate the 95% CIs estimated from the longitudinal linear mixed model shown in eTable 6 in Supplement 2.

The TMT-B sample mean (SE) for time to completion before initiating chemotherapy was 93.1 (5.6) seconds for the statin group and 88.6 (6.0) seconds for the placebo group. From baseline to 24 months, we observed a statistically significant mean decrease of 10.2 seconds (improvement) in time to completion of TMT-B within the statin group (95% CI, 1.9-18.5; *P* = .02) but did not find a significant change in the placebo group (mean, 0.2 seconds [95% CI, −8.5 to 8.1]; *P* = .96). In TMT-B minus TMT-A–derived scores, we observed a significant decrease over time in the statin group (baseline minus 24-month difference, 7.7 seconds [95% CI, 0.5-14.8]; *P* = .04) but did not find a significant change over time in the placebo group. For TMT-B, we observed no significant difference between the placebo and statin groups (eTable 6 in Supplement 2; Figure 2) over the 24-month study period for time to completion.

We evaluated our assessments of change in TMT-A and TMT-B after accounting for age, education, income, race and ethnicity, cancer type, diastolic blood pressure, HDL, glucose, and tumor necrosis factor α. Unadjusted results for all measures are shown in eTable 7 in Supplement 2. We observed that an increase in age was associated with longer time to completion, such that each 1-year increase in age was associated with a 0.24-second increase (95% CI, 0.11-0.37 seconds) and 0.83-second increase (95% CI, 0.50-1.17 seconds) in TMT-A and TMT-B, respectively (*P* < .001). The effect of placebo vs statin on TMT-B over time was not differentially associated with age younger than 52 years or 52 years or older. Participants with higher levels of education than high school had significantly faster completion times for TMT-B, such that college-educated patients completed the test a mean of 19.87 seconds faster (95% CI, 9.72-30.01 seconds; *P* < .001), and graduate-educated patients completed the test a mean of 13.07 seconds faster (95% CI, 0.22-25.91 seconds; *P* = .046). Furthermore, compared with White participants, Black participants had a slightly longer, but nonsignificant time to completion of 10.8 seconds (95% CI, −1.2 to 22.8 seconds; *P* = .08), while participants of a race other than Black or White had a significantly slower time to completion of 37.3 seconds (95% CI, 14.2-60.4 seconds; *P* = .002).

We found a similar difference in change over time with respect to TMT-B error count between the statin and placebo groups (eTable 8 and eFigure 3 in Supplement 2). The TMT-B sample mean (SE) for rate of errors before initiating chemotherapy was 0.42 (0.09) for the statin group and 0.37 (0.08) for the placebo group. Notably, a small, but nonsignificant decline of 0.12 errors from baseline to 24 months (95% CI, −0.03 to −0.27; *P* = .06) was observed in the statin group. However, we did not find such a change in the placebo group. Additionally, we observed that higher age was associated with a greater error count (incidence rate ratio, 1.03 [95% CI, 1.01-1.05]; *P* < .001), while a college-level education was associated with fewer errors (incidence rate ratio, 0.52 [95% CI, 0.32-0.82]; *P* = .005) compared with a high school education.

It took a mean of 8.3 more seconds (95% CI, 5.07-11.53 seconds; *P* < .001) to complete the TMT-B for every 10 years in age. Of note, similar improvements in TMT-B times and errors occurred among younger and older female participants receiving statins (interaction *P* = .50).

**Figure 3.**
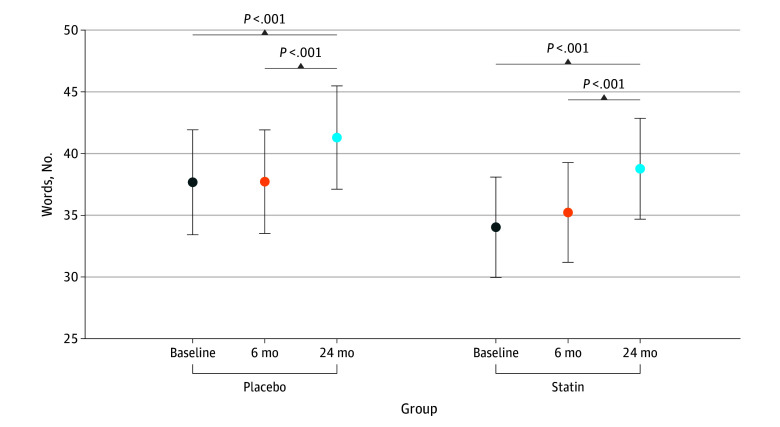
Word Frequency on the Controlled Oral Word Association Test Error bars indicate the 95% CIs estimated from the generalized linear mixed model shown in eTable 9 in Supplement 2.

For COWA scores, we observed in both groups a significant increase from baseline to 24 months, with the placebo group scores increasing by 3.62 points (95% CI, 1.71-5.54 points; *P* < .001) and the statin group scores increasing by 4.74 points (95% CI, 2.69-6.79 points; *P* < .001) (Figure 3; eTable 9 in Supplement 2). However, the difference in this change between groups did not significantly differ (interaction *P* = .67). Consistent with TMT-B results, participants with a college- or graduate-level education had better performance scores, with an increase of 7.27 points (95% CI, 3.69-10.85 points; *P* < .001) and 8.77 points (95% CI, 4.26-13.28 points; *P* < .001), respectively, compared with those with a high school–level education.

## Discussion

This preplanned secondary analysis of PREVENT RCT participants who received doxorubicin for the treatment of breast cancer or lymphoma and daily statin therapy vs placebo found that (1) attention, verbal fluency, and executive function did not worsen in the 24 months after receipt of doxorubicin chemotherapy; (2) those receiving doxorubicin and statins did not experience an additional decline in cognitive function compared with those receiving placebo; and (3) executive function scores slightly improved at 24 months for those receiving statins compared with placebo. Not unexpectedly, mean times for both TMT-A and TMT-B were higher (indicating poorer performance) than for normative values for the population without cancer.^25^ Prior studies have shown 20-second to 29-second differences in completion time of TMT-B among patients with brain injury vs healthy control individuals.^26,27^ Here, the TMT-B sample mean for time to completion prior to initiating chemotherapy was 93.1 seconds for the statin group and 88.6 seconds for the placebo group. Prior research has implicated mental tracking and motor control in TMT-B time scores and error rates.^28,29^ Error rates are difficult to interpret in isolation, as errors are common in normal adults. A previous study reported that at least 1 error was made in 34.8% of control participants.^30^ Here, the TMT-B sample mean for rate of errors prior to initiating chemotherapy was 0.42 for the statin group and 0.37 for the placebo group.

Most statin users are older adults (>65 years of age) with cardiovascular disease who are at higher risk of cognitive decline due to age and comorbidities.^3^ Prior studies have found that statins may adversely alter cognitive performance, although recent findings have shown that statins may offer neuroprotection.^31^ A systematic review of RCTs and observational studies focusing on older individuals showed a lack of consistent evidence of adverse cognitive effects of statin use, including deterioration in global cognition or specific cognitive domains.^32^ Of note, our study shows that age was significantly associated with executive function (eTable 6 in Supplement 2).

Even mild executive function loss could impact treatment decisions and coping with life changes.^33^ A prior cohort study of more than 26 000 US adults found that compared with men, women may experience quicker declines in executive function.^34^ Our study suggests that statin administration during receipt of anthracycline-based chemotherapy may offer women some protection against decline in executive function. As noted, this change in executive function in women receiving statins and doxorubicin occurred in both younger and older women (aged <52 years or ≥52 years). Since this dichotomy in age was used to identify predoxorubicin treatment before and after menopause,^35^ these results suggest that statins may be beneficial for executive function in these patients regardless of menopausal status.

Prior studies that examined individuals at risk for cardiovascular disease reported that both elevated and lower naturally occurring cholesterol levels are associated with reduced performance on measures of executive function.^36,37,38,39^ Interestingly, our results show that improvements in TMT-B times coincided with declines in LDL cholesterol levels (Table 2). Although chronic low-grade inflammation is commonly associated with cardiovascular disease risk and cognitive decline,^40^ in our study, we did not observe an association between serum C-reactive protein, tumor necrosis factor α, and interleukin 6^40,41,42,43,44,45,46^ and either COWA, TMT-A, or TMT-B results.

### Limitations

This study had several limitations. First, assessing cognitive function repeatedly increases the potential for practice effects.^47,48^ Our use of alternative forms of the COWA may have mitigated unwanted effects, but there is not an alternate version of the TMT. Our randomized, double-blind study design would distribute this potential effect equally across study groups. Second, our relatively young study population represented primarily well-educated White women, limiting the generalizability of our results. Further study is needed to examine the impact of statins and doxorubicin on cognitive function among men and people of other racial backgrounds. Third, we did not control for a family-wise error rate across the hypotheses tested. While these hypotheses regarding cognition changes and treatment were specified a priori, we recognize that with multiple hypotheses assessed, the collective probability of a type I error across the analysis was greater than the independently assumed α of .05. Our findings should be interpreted within this context; significant findings without a multiple comparison adjustment highlight potential associations that may exist, but we acknowledge more should be done in diverse and larger populations to make more definitive claims.

## Conclusions

While prior research has indicated that statins may negatively impact cognitive function, this preplanned secondary analysis of the PREVENT RCT did not find a significant difference in cognitive function in participants receiving statins vs placebo while also receiving doxorubicin. The study found no evidence of statin-associated cognitive decline in primarily educated White women over 24 months after receiving anthracycline-based chemotherapy for lymphoma or breast cancer. The addition of 40 mg/d of atorvastatin was not adversely associated with attention, verbal fluency, or executive functions compared with placebo. Notably, within-group analyses suggested that statins may contribute to heightened scores on tests measuring executive function. Future studies are needed to determine whether statin administration preserves cognitive function during and after receipt of anthracycline-based chemotherapy for other forms of cancer.

## Notes

Supplement 1.
Trial Protocol

Supplement 2. **eFigure 1.** Flowchart of Included Study Participants **eTable 1.** Baseline Characteristics of Those With and Without Cognitive Outcome Data in the Overall Population **eTable 2.** Baseline Characteristics of Those With and Without Cognitive Outcome Data in the Analyzed Population **eTable 3. **Cognitive Assessment Scores Across All Three Visits by Group: Anthracycline Based Chemotherapy and Placebo or Anthracycline Based Chemotherapy and Statin **eTable 4. **Part A Trail Making Test (TMT-A), Time in Seconds to Complete **eTable 5.** Part A Trail Making Test (TMT-A), Number of Errors **eFigure 2.** Part A Trail Making Test (TMT-A), Number of Errors **eFigure 3.** Estimated Part B Trail Making Test (TMT-B) Errors **eTable 6.** Part B Trail Making Test (TMT-B), Time in Seconds to Complete **eTable 7.** Unadjusted Within-Group Changes From Baseline to 24 Months on Cognitive Assessment Scores Across All Measures **eTable 8.** Part B Trail Making Test (TMT-B), Number of Errors **eTable 9.** COWA-Estimated Frequency (Words)

Supplement 3.
Data Sharing Statement

## References

[1] Kim SW, Kang HJ, Jhon M, . Statins and inflammation: new therapeutic opportunities in psychiatry. Front Psychiatry. 2019;10:103. doi:10.3389/fpsyt.2019.00103 30890971 PMC6413672

[2] Gurwitz JH, Go AS, Fortmann SP. Statins for primary prevention in older adults: uncertainty and the need for more evidence. JAMA. 2016;316(19):1971-1972. doi:10.1001/jama.2016.15212 27838724 PMC5429025

[3] Chadha B, Frishman WH. Review of the protective effects of statins on cognition. Cardiol Rev. 2021;29(6):328-335. doi:10.1097/CRD.0000000000000359 33027065

[4] Gauthier JM, Massicotte A. Statins and their effect on cognition: let’s clear up the confusion. Can Pharm J (Ott). 2015;148(3):150-155. doi:10.1177/1715163515578692 26150888 PMC4483758

[5] Bitzur R. Remembering statins: do statins have adverse cognitive effects? Diabetes Care. 2016;39(suppl 2):S253-S259. doi:10.2337/dcS15-3022 27440840

[6] Asher A, Myers JS. The effect of cancer treatment on cognitive function. Clin Adv Hematol Oncol. 2015;13(7):441-450. 26353040

[7] Duff K, Schoenberg MR, Scott JG, Adams RL. The relationship between executive functioning and verbal and visual learning and memory. Arch Clin Neuropsychol. 2005;20(1):111-122. doi:10.1016/j.acn.2004.03.003 15620817

[8] Radin A, Ganz PA, Van Dyk K, Stanton AL, Bower JE. Executive functioning and depressive symptoms after cancer: the mediating role of coping. Psychosom Med. 2021;83(3):291-299. doi:10.1097/PSY.0000000000000926 33657085 PMC8691137

[9] Rodríguez Martín B, Fernández Rodríguez EJ, Rihuete Galve MI, Cruz Hernández JJ. Study of chemotherapy-induced cognitive impairment in women with breast cancer. Int J Environ Res Public Health. 2020;17(23):8896. doi:10.3390/ijerph17238896 33265966 PMC7730121

[10] Avila MS, Siqueira SRR, Ferreira SMA, Bocchi EA. Prevention and treatment of chemotherapy-induced cardiotoxicity. Methodist Debakey Cardiovasc J. 2019;15(4):267-273. doi:10.14797/mdcj-15-4-267 31988687 PMC6977564

[11] Hundley WG, D’Agostino R Jr, Crotts T, . Statins and left ventricular ejection fraction following doxorubicin treatment. NEJM Evid. 2022;1(9). doi:10.1056/EVIDoa2200097 36908314 PMC9997095

[12] Neilan TG, Quinaglia T, Onoue T, . Atorvastatin for anthracycline-associated cardiac dysfunction: the STOP-CA randomized clinical trial. JAMA. 2023;330(6):528-536. doi:10.1001/jama.2023.11887 37552303 PMC10410476

[13] Wefel JS, Vardy J, Ahles T, Schagen SB. International Cognition and Cancer Task Force recommendations to harmonise studies of cognitive function in patients with cancer. Lancet Oncol. 2011;12(7):703-708. doi:10.1016/S1470-2045(10)70294-1 21354373

[14] Benedict RHB, Schretlen D, Groninger L, Brandt J. Hopkins Verbal Learning Test – Revised: normative data and analysis of inter-form and test-retest reliability. Clin Neuropsychol. 2010;12(1):43-55. doi:10.1076/clin.12.1.43.1726

[15] Brandt J. The Hopkins Verbal Learning Test: development of a new memory test with six equivalent forms. Clin Neuropsychol. 1991;5(2):125-142. doi:10.1080/13854049108403297

[16] Kesler S, Hadi Hosseini SM, Heckler C, . Cognitive training for improving executive function in chemotherapy-treated breast cancer survivors. Clin Breast Cancer. 2013;13(4):299-306. doi:10.1016/j.clbc.2013.02.004 23647804 PMC3726272

[17] Reitan RM. Validity of the Trail Making Test as an indicator of organic brain damage. Percept Mot Skills. 1958;8(3):271-276. doi:10.2466/pms.1958.8.3.271

[18] Suchy-Dicey AM, Vo TT, Oziel K, . Psychometric Properties of Controlled Oral Word Association (COWA) Test and associations with education and bilingualism in American Indian adults: The Strong Heart Study. Assessment. 2024;31(3):745-757. doi:10.1177/10731911231180127 37338127 PMC10840386

[19] Benton AL, Hamsher dSK, Sivan AB. Controlled Oral Word Association Test (COWAT). APA PsychTests; 1983. doi:10.1037/t10132-000

[20] Ruff RM, Light RH, Parker SB, Levin HS. Benton Controlled Oral Word Association Test: reliability and updated norms. Arch Clin Neuropsychol. 1996;11(4):329-338. doi:10.1093/arclin/11.4.329 14588937

[21] Guo Z, Zheng Y, Geng J, . Unveiling the link between systemic inflammation markers and cognitive performance among older adults in the US: a population-based study using NHANES 2011-2014 data. J Clin Neurosci. 2024;119:45-51. doi:10.1016/j.jocn.2023.11.004 37979310

[22] Stekhoven DJ, Bühlmann P. missForest–Non-parametric missing value imputation for mixed-type data. Bioinformatics. 2012;28(1):112-118. doi:10.1093/bioinformatics/btr597 22039212

[23] Stekhoven DJ. Package ‘missForest’: nonparametric missing value imputation using random forest. R package version 1.5. The Comprehensive R Archive Network. 2022. Accessed July 30, 2025. https://cran.r-project.org/web/packages/missForest/missForest.pdf

[24] Groll A. glmmLasso: Variable selection for generalized linear mixed models by L1-penalized estimation. R package version 1.6.3. The Comprehensive R Archive Network. 2023. Accessed July 30, 2025. https://CRAN.R-project.org/package=glmmLasso

[25] Tombaugh TN. Trail Making Test A and B: normative data stratified by age and education. Arch Clin Neuropsychol. 2004;19(2):203-214. doi:10.1016/S0887-6177(03)00039-8 15010086

[26] Klusman LE, Cripe LI, Dodrill CB. Analysis of errors on the Trail Making Test. Percept Mot Skills. 1989;68(3 pt 2):1199-1204. doi:10.2466/pms.1989.68.3c.1199 2762086

[27] Chan E, MacPherson SE, Robinson G, . Limitations of the Trail Making Test part-B in assessing frontal executive dysfunction. J Int Neuropsychol Soc. 2015;21(2):169-174. doi:10.1017/S135561771500003X 25697352

[28] Ashendorf L, Jefferson AL, O’Connor MK, Chaisson C, Green RC, Stern RA. Trail Making Test errors in normal aging, mild cognitive impairment, and dementia. Arch Clin Neuropsychol. 2008;23(2):129-137. doi:10.1016/j.acn.2007.11.005 18178372 PMC2693196

[29] Mahurin RK, Velligan DI, Hazleton B, Mark Davis J, Eckert S, Miller AL. Trail Making Test errors and executive function in schizophrenia and depression. Clin Neuropsychol. 2006;20(2):271-288. doi:10.1080/13854040590947498 16690547

[30] Ruffolo LF, Guilmette TJ, Willis GW. Comparison of time and error rates on the Trail Making Test among patients with head injuries, experimental malingerers, patients with suspect effort on testing, and normal controls. Clin Neuropsychol. 2000;14(2):223-230. doi:10.1076/1385-4046(200005)14:2;1-Z;FT223 10916197

[31] Jamshidnejad-Tosaramandani T, Kashanian S, Al-Sabri MH, Kročianová D, Clemensson LE, Gentreau M, Schiöth HB. Statins and cognition: modifying factors and possible underlying mechanisms. Front Aging Neurosci. 2022;14:968039. doi:10.3389/fnagi.2022.968039 36046494 PMC9421063

[32] Adhikari A, Tripathy S, Chuzi S, Peterson J, Stone NJ. Association between statin use and cognitive function: A systematic review of randomized clinical trials and observational studies. J Clin Lipidol. 2021;15(1):22-32.e12. doi:10.1016/j.jacl.2020.10.007 33189626

[33] Visovatti MA, Reuter-Lorenz PA, Chang AE, Northouse L, Cimprich B. Assessment of cognitive impairment and complaints in individuals with colorectal cancer. Oncol Nurs Forum. 2016;43(2):169-178. doi:10.1188/16.ONF.43-02AP 26906128 PMC9241527

[34] Levine DA, Gross AL, Briceño EM, . Sex differences in cognitive decline among US adults. JAMA Netw Open. 2021;4(2):e210169. doi:10.1001/jamanetworkopen.2021.0169 33630089 PMC7907956

[35] Koothirezhi R, Ranganathan S. Postmenopausal Syndrome. StatPearls Publishing LLC; 2024. 32809675

[36] Elias PK, Elias MF, D’Agostino RB, Sullivan LM, Wolf PA. Serum cholesterol and cognitive performance in the Framingham Heart Study. Psychosom Med. 2005;67(1):24-30. doi:10.1097/01.psy.0000151745.67285.c2 15673620

[37] Reijmer YD, van den Berg E, Dekker JM, . Development of vascular risk factors over 15 years in relation to cognition: the Hoorn Study. J Am Geriatr Soc. 2012;60(8):1426-1433. doi:10.1111/j.1532-5415.2012.04081.x 22861348

[38] Gendle MH, Spaeth AM, Dollard SM, Novak CA. Functional relationships between serum total cholesterol levels, executive control, and sustained attention. Nutr Neurosci. 2008;11(2):84-94. doi:10.1179/147683008X301469 18510808

[39] Leritz EC, McGlinchey RE, Salat DH, Milberg WP. Elevated levels of serum cholesterol are associated with better performance on tasks of episodic memory. Metab Brain Dis. 2016;31(2):465-473. doi:10.1007/s11011-016-9797-y 26873100 PMC4913474

[40] Kipinoinen T, Toppala S, Rinne JO, Viitanen MH, Jula AM, Ekblad LL. Association of midlife inflammatory markers with cognitive performance at 10-year follow-up. Neurology. 2022;99(20):e2294-e2302. doi:10.1212/WNL.0000000000201116 36195448 PMC9694835

[41] Choi J, Joseph L, Pilote L. Obesity and C-reactive protein in various populations: a systematic review and meta-analysis. Obes Rev. 2013;14(3):232-244. doi:10.1111/obr.12003 23171381

[42] Ahmed B, Sultana R, Greene MW. Adipose tissue and insulin resistance in obese. Biomed Pharmacother. 2021;137:111315. doi:10.1016/j.biopha.2021.111315 33561645

[43] Hotamisligil GS, Shargill NS, Spiegelman BM. Adipose expression of tumor necrosis factor-alpha: direct role in obesity-linked insulin resistance. Science. 1993;259(5091):87-91. doi:10.1126/science.7678183 7678183

[44] Trayhurn P, Wood IS. Adipokines: inflammation and the pleiotropic role of white adipose tissue. Br J Nutr. 2004;92(3):347-355. doi:10.1079/BJN20041213 15469638

[45] Graßmann S, Wirsching J, Eichelmann F, Aleksandrova K. Association between peripheral adipokines and inflammation markers: a systematic review and meta-analysis. Obesity (Silver Spring). 2017;25(10):1776-1785. doi:10.1002/oby.21945 28834421

[46] Schäffler A, Schölmerich J, Salzberger B. Adipose tissue as an immunological organ: toll-like receptors, C1q/TNFs and CTRPs. Trends Immunol. 2007;28(9):393-399. doi:10.1016/j.it.2007.07.003 17681884

[47] Jung SO, Kim JEE, Kim HJ. Assessing objective cognitive impairments in cancer survivors: features and validity of measures for research and clinical applications. Asia Pac J Oncol Nurs. 2023;10(11):100309. doi:10.1016/j.apjon.2023.100309 37928414 PMC10622612

[48] Calamia M, Markon K, Tranel D. Scoring higher the second time around: meta-analyses of practice effects in neuropsychological assessment. Clin Neuropsychol. 2012;26(4):543-570. doi:10.1080/13854046.2012.680913 22540222

