# Supplementary material for: Preserved Cognitive Function After Statin Administration During Cancer Treatment With Doxorubicin: A Secondary Analysis of a Randomized Clinical Trial
Source: JAMA Netw Open. 2025 Oct 21;8(10):e2538325. doi: 10.1001/jamanetworkopen.2025.38325 (PMC12541534; doi:10.1001/jamanetworkopen.2025.38325)
Supplement: Supplement 1. — Trial Protocol [file jamanetwopen-e2538325-s001.pdf]

**NCI Protocol #:**	**WF-98213**
**WF Protocol #:**	**98213**
**IND # (If applicable):**	**N/A**

Protocol title: Preventing Anthracycline Cardiovascular Toxicity with Statins (PREVENT)

**Organization Name:**	**Wake Forest School of Medicine**
**Protocol Principal Investigator:**	**W. Gregory Hundley, MD**<br>Departments of Internal Medicine-Cardiology and Radiology<br>1 Medical Center Blvd.<br>Winston-Salem, NC 27157<br>Telephone: (336) 716-0607<br>Fax: (336) 716-9188<br>E-mail address: ghundley@wakehealth.edu
**Consortium Name:**	**Wake Forest NCORP Research Base**
**Name of Consortium Multi-Principal Investigator:**	**Glenn Lesser, MD**<br>Wake Forest School of Medicine<br>Department of Internal Medicine<br>Section on Hematology and Oncology<br>Medical Center Boulevard<br>Winston-Salem, NC 27157<br>Telephone: (336) 716-0891<br>Fax: (336) 716-6275<br>E-mail address: glesser@wakehealth.edu
**Organization:**	**Wake Forest School of Medicine**
**Co-Investigator:**	**Stephen R. Rapp, PhD**<br>Psychiatry and Behavioral Medicine<br>Medical Center Boulevard<br>Winston-Salem, NC 27157<br>Telephone: (336) 716-6995<br>Fax: (336) 716-6830<br>E-mail address: srapp@wakehealth.edu
**Organization:**	**Wake Forest School of Medicine**
**Co-Investigator:**	**Heidi Klepin, MD**<br>Department of Internal Medicine<br>Section on Hematology and Oncology<br>Medical Center Boulevard<br>Winston-Salem, NC 27157<br>Telephone: (336) 716-5772<br>Fax: (336) 716-5687<br>E-mail: hklepin@wakehealth.edu

Organization: **Wake Forest School of Medicine**
Co-Investigator: **David Herrington, MD**
Department of Internal Medicine
Section on Cardiology
Medical Center Boulevard
Winston-Salem, NC 27157
Telephone: (336) 716-4950
Fax: (336) 716-9188
E-mail Address: dherring@wakehealth.edu

Organization: **Wake Forest School of Medicine**
Co-Investigator: **Joseph Yeboah, MBChB**
Department of Internal Medicine
Section on Cardiology
Medical Center Boulevard
Winston-Salem, NC 27157
Telephone: (336) 716-7015
Fax: (336) 716-5324
E-mail address: jyeboah@wakehealth.edu

Organization: **Wake Forest School of Medicine**
Co-Investigator: **Sujethra Vasu, MBBS**
Department of Internal Medicine
Section on Cardiology
Medical Center Boulevard
Winston-Salem, NC 27157
Telephone: (336) 716-7015
Fax: (336) 716-5324
E-mail: svasu@wakehealth.edu

Organization: **Wake Forest School of Medicine**
Protocol Statistician: **Ralph D'Agostino, Jr., PhD**
Biostatistics Core Director
Department of Biostatistical Sciences
Division of Public Health Sciences
Medical Center Boulevard
Winston-Salem, NC 27157
Telephone: (336) 716-9410
Fax: (336) 716-5425
Email address: rdagosti@wakehealth.edu

Organization: **Wake Forest School of Medicine**
Site Coordinator: **Tammy Vogler, RN**
Department of Social Sciences and Health Policy
NCORP Research Base – 525@Vine, 4th floor
P.O. Box 573152
Medical Center Boulevard
Winston-Salem, NC 27157
Telephone: (336) 713-6907
Fax: (336) 716-6275
E-mail address: tvogler@wakehealth.edu

Organization: **Mayo Clinic**
Kathryn J. Ruddy, MD, MPH
Mayo Clinic
200 First Street SW
Rochester, MN 55905
Telephone: (507) 266-9160
Email: Ruddy.Kathryn@mayo.edu

Grant: 1R01 HL118740-01
NCI/National Heart Lung Blood Institute
6130 Executive Blvd., Room 2117
Bethesda, MD 20892 (For FedEx, use Rockville, MD 20852)
(301) 496-8563

3U10CA081851-12
NCI/Division of Cancer Prevention
Community Oncology & Prevention Trials Research Group
9609 Medical Center Drive, Room 5E502
Rockville, MD 20850
(240) 276-7050

Participating Organizations:
- **WAKE** / Wake Forest Research Base NCORP
- **ALLIANCE** / Alliance for Clinical Trials in Oncology
- **ECOG-ACRIN /** ECOG-ACRIN Cancer Research Group

Agent(s)/Supplier:

Initial Approval Dates: **PRC:** 06/13/13 **NCI:** 09/04/13
FDA: 06/28/13 (exempt) **IRB:** 08/02/13

Activation Date: **WF:** 02/05/2014
Sites: 02/10/2014

NCI Version Date: 03/27/2020

Renewal Dates: **CR1:** 06/04/14
CR2: 05/11/15
CR3: 04/25/16
CR4: 08/21/17
CR5: 07/03/18
CR6: 05/24/19

IRB Amendment Approval Dates:

Amend 1: 02/05/14
Amend 2: 02/10/14
Amend 3: 02/19/14
Amend 4: 05/05/14
Amend 5: 08/04/14
Amend 6: 08/28/14
Amend 7: 09/30/14 - WF Staff Change
Amend 8: 02/16/15
Amend 9: 04/17/15
Amend 10: 06/09/15
Amend 11: 06/24/15 - WF Staff Change
Amend 12: 07/29/15
Amend 13: 01/12/16 - WF Staff Change
Amend 14: 03/15/16 - WF Staff Change
Amend 15: 04/08/16
Amend 16: 04/21/16 - WF Staff Change
Amend 17: 07/25/16
Amend 18: 10/12/16
Amend 19: 11/01/16 - WF Staff Change
Amend 20: 03/21/17
Amend 21: 09/22/17
Amend 22: 10/25/17
Amend 23: 10/29/18 – WF Staff Change
Amend 24: 09/21/18 – flyer to pdf
Amend 25: 11/21/18 – WF Staff Change
Amend 26: 01/14/19 – WF Staff Change
Amend 27: 04/22/19 – Testing of stored samples
Amend 28: 07/10/19 – WF Staff Change
Amend 29: 09/21/16
Amend 30: 02/14/20 – WF Staff Change
Amend 31: 03/03/20 – WF Staff Change
Amend 32:

<table>
<tr><th colspan="3">CONTACT INFORMATION</th></tr>
<tr><th>For regulatory requirements:</th><th>For patient enrollments:</th><th>For study data submission:</th></tr>
<tr><td>Regulatory documentation must be submitted to the CTSU via the Regulatory Submission Portal. (Sign in at www.ctsu.org, and select Regulatory > Regulatory Submission.

Institutions with patients waiting that are unable to use the Portal should alert the CTSU Regulatory Office immediately at 1-866-651-2878 to receive further instruction and support.

Contact the CTSU Regulatory Help Desk at 1-866-651-2878 for regulatory assistance.</td><td>Please refer to the patient enrollment section 6.8 of the protocol for detailed instructions.</td><td>Data forms will be submitted to the WF NCORP Research Base.

NCORP Research Base Data
Wake Forest Baptist Medical Center
Building 525@Vine, 4th floor
Medical Center Boulevard
Winston-Salem, NC 27157

Fax to (336) 713-6476 according to the timetable in Section 11.1

Do not submit study data or forms to CTSU Data Operations. Do not copy the CTSU on data submissions.</td></tr>
<tr><td colspan="3">The most current version of the study protocol and all supporting documents must be downloaded from the protocol-specific Web page of the CTSU Member Web site located at https://www.ctsu.org. Access to the CTSU members' website is managed through the Cancer Therapy and Evaluation Program - Identity and Access Management (CTEP-IAM) registration system and requires user log on with CTEP-IAM username and password. Permission to view and download this protocol and its supporting documents is restricted and is based on person and site roster assignment housed in the CTSU RSS.</td></tr>
<tr><td colspan="3">For clinical questions (i.e. patient eligibility or treatment-related) contact the Study PI Dr, Gregory Hundley, ghundley@wakehealth.edu</td></tr>
<tr><td colspan="3">For non-clinical questions (i.e. unrelated to patient eligibility, treatment, or clinical data submission) contact the CTSU Help Desk by phone or e-mail:
CTSU General Information Line – 1-888-823-5923, or ctsucontact@westat.com. All calls and correspondence will be triaged to the appropriate CTSU representative.</td></tr>
<tr><td colspan="3"></td></tr>
<tr><td colspan="3">The CTSU Website is located at https://www.ctsu.org.</td></tr>
</table>

SCHEMA

Preventing Anthracycline Cardiovascular Toxicity with Statins (PREVENT)

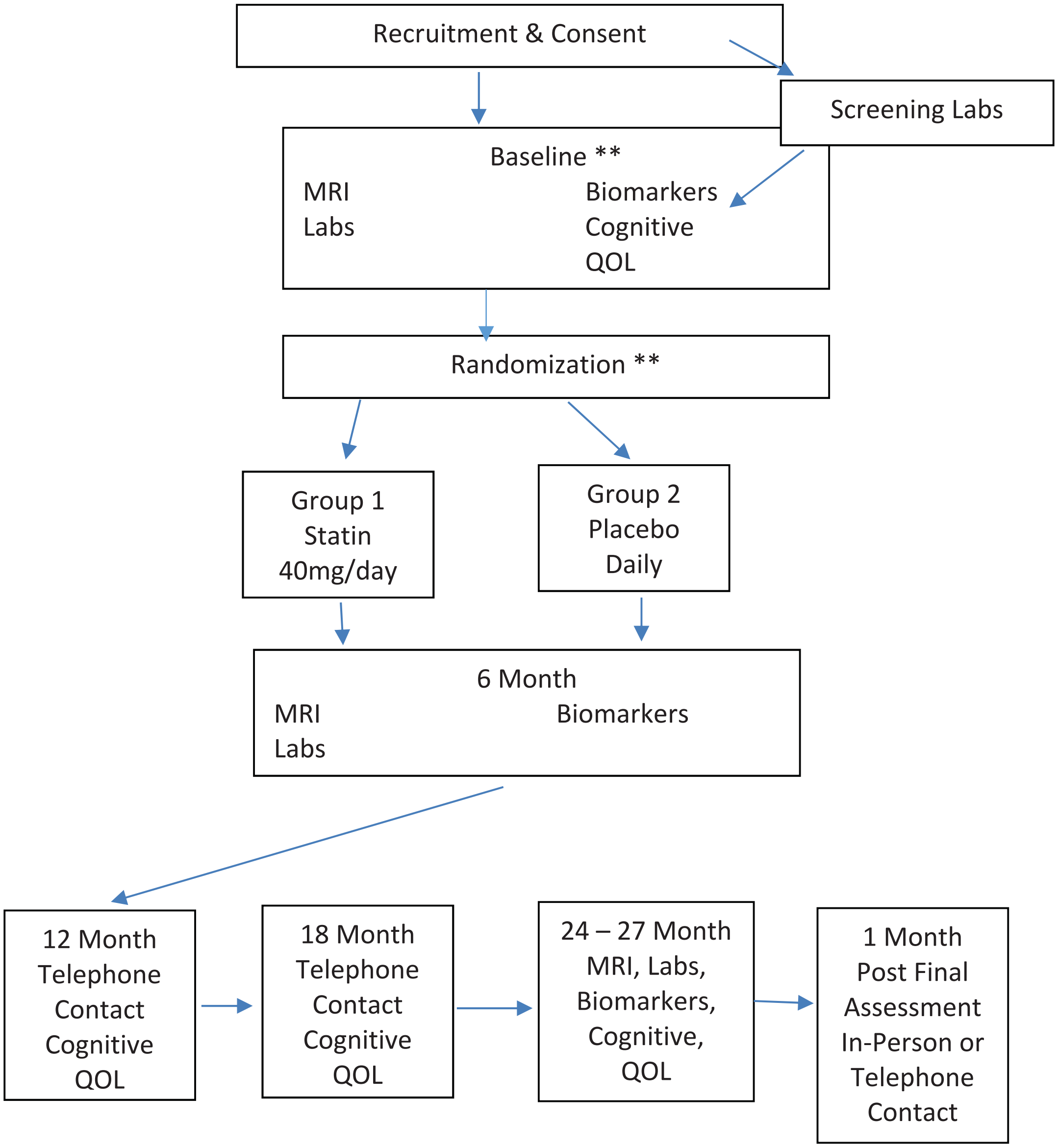

Stratification Factors: Breast Cancer Doxorubicin Total dose: ≤ 239 mg/m^2 vs. ≥ 240 mg/m^2

or

Breast Cancer Epirubicin Total dose: ≤ 399 mg/m^2 vs. ≥ 400 mg/m^2

or

Lymphoma Doxorubicin Total dose: ≤ 239 mg/m^2 vs. ≥ 240 mg/m^2

** Due to the scheduling complexity of protocol requirements prior to the start of the first chemotherapy treatment, baseline procedures and randomization may be performed at the sites discretion once the patient has signed the consent. The patient should be registered within 30 days of screening labs.

Study Sample: n=278

Study Duration: at least 24 months per subject but no greater than 27 months.

Patients completing at least 24 months of study medication will be followed for an additional 30 days after the last dose of study medication to evaluate any study related adverse events.

Brief Eligibility Criteria:

- Stage I-III breast cancer (including inflammatory and newly diagnosed recurrent breast cancer) or lymphoma Stage I-IV
- Scheduled to receive chemotherapy with any anthracycline
- Age: ≥ 21 years of age

INDEX

COVER PAGE

SCHEMA

1. OBJECTIVES 9

2. BACKGROUND 11

3. SUMMARY OF STUDY PLAN 15

4. PARTICIPANT SELECTION 17

5. AGENT ADMINISTRATION 19

6. PHARMACEUTICAL INFORMATION 21

7. CLINICAL EVALUATIONS AND PROCEDURES 31

8. PROTOCOL SPECIFIC TRAINING REQUIREMENTS 38

9. SPECIMEN MANAGEMENT 39

10. REPORTING ADVERSE EVENTS 39

11. STUDY MONITORING 42

12. STATISTICAL CONSIDERATIONS 44

REFERENCES

Consent Form
FDA Letter

APPENDICES

1. Data Submission Checklist
2. Eligibility Checklist-Enrollment
3. CV Risk Calculator and Instructions
4. Patient Medication Diary
5. Screening Form
6. Flow Sheet / Addenda
7. Current Medication Form
8. Radiation Treatment Form
9. Chemotherapy Treatment Form
10. Lab Instructions
11. Telephone Contact Form
12. Physician Statin Update (Includes Open Label Form)
13. Baseline Neurocognitive Booklet
14. 6 Month Neurocognitive Booklet
15. 12 Month Booklet
16. 18 Month Booklet
17. 24 Month Neurocognitive Booklet
18. Early Withdrawal Form
19. 1 Month Contact Form

1. OBJECTIVES

The overall goal of this proposal is to determine if atorvastatin administration attenuates deterioration in left ventricular ejection fraction (LVEF) in men and women receiving Anthracycline-based chemotherapy. Left ventricular (LV) dysfunction precedes the development of congestive heart failure (CHF), a major contributor to cardiovascular (CV) events, and the 2nd leading cause of morbidity and mortality for 7-year survivors treated with adjuvant chemotherapy for breast cancer.[1-7] This heightened incidence of CV events threatens to offset cancer-related survival and raise healthcare expenditures for breast cancer survivors. An intervention that reduces LV dysfunction and CHF would be *significant*.[1-7]

Anthracycline-based chemotherapy is an important component of therapy for those with advanced disease;[8] for breast cancer alone, 5-year survival exceeds 70% after Anthracycline-based adjuvant therapy.[9] Not only is doxorubicin an essential element in modern therapy of breast, soft tissue sarcomas and other solid tumors,[i,ii] it is thought to be an essential element of curative combination chemotherapy for acute leukemia, Hodgkin's disease, non-Hodgkin's lymphoma, and many childhood cancers.[iii,iv] Thus, for many individuals with advanced stages of cancer, Doxorubicin serves as an important part of their medical regimen.

The cytotoxic anti-tumor effects from Anthracycline-based adjuvant therapy are related to its interactions with the enzyme topoisomerase-IIα, production of double-strand DNA breaks, and the generation of intracellular cytotoxic free radicals.[10] Unfortunately, in cardiomyocytes, these cytotoxic free radicals promote oxidative and nitrosative stress that, in combination with other Anthracycline-based adjuvant therapy effects (inflammation and neurohormonal activation), promote LV dysfunction, myocardial replacement fibrosis, CHF, and CV events.[11] Primary prevention strategies that reduce Anthracycline-based chemotherapy mediated myocellular oxidative/nitrosative stress could diminish LV dysfunction and the subsequent CHF often experienced by patients after Anthracycline-based adjuvant therapy. No such strategies currently exist.

In the secondary prevention setting, the notion that modulating Anthracycline-based chemo therapy mediated oxidative/nitrosative stress can preserve LV function has been applied to women with poor resting LV function in need of Anthracycline-based adjuvant therapy.[12,13] In these women, intravenous pre-treatment with dexrazoxane, a free iron binding agent that reduces the generation of reactive oxygen and consequent nitrogen species, decreases further LV dysfunction and CV events.[12,13] This therapy is not utilized for primary prevention because of its expense, associated side effects, and the potential concern it may reduce anthracycline-mediated tumor regression.

Several lines of evidence suggest that generic, inexpensive, oral 3-hydroxy-3-methylglutaryl-coenzyme A (HMG-CoA) reductase inhibitors (statins) may prevent cardiomyocyte injury during and after Anthracycline-based adjuvant therapy.[14] This class of drugs is used commonly to treat hypercholesterolemia, but also reduces oxidative/nitrosative stress, inflammatory cytokines, and circulating neurohormones.[15,16] In animal models, statins diminish LV dysfunction and oxidative/nitrosative stress after Anthracycline-based chemotherapy.[14] In our pilot studies, we find that LV injury occurs early (in the first 6 months of Anthracycline-based chemo therapy administration), and LV performance is preserved in men and women receiving Anthracycline-based --chemo therapy when statins are co-administered for pre-existing CV disease during these 6 months.[19] Unlike some cardioprotective medications, statins do not promote hypotension, and recent data indicate that breast cancer recurrence does not increase among 18,000 5-year survivors receiving statins during and after treatment for breast cancer.[18]

Given the propensity of statins to ameliorate LV dysfunction and reduce CHF and other CV events, we seek to conduct a clinical trial to determine if statin therapy attenuates the early onset of LV dysfunction in men and women treated with Anthracycline-based chemotherapy for breast cancer or lymphoma. Accordingly, we propose the following.

Primary Objectives

Specific Aim 1: To determine if atorvastatin administration preserves LVEF 24 months after initiation of Anthracycline-based chemo-therapy for breast cancer or lymphoma.

Specific Aim 2: To determine if baseline to 6-month differences in LVEF predict baseline to 24-month differences in LVEF after Anthracycline-based chemotherapy and concomitant atorvastatin therapy.

To achieve these aims, we will perform a double-blind, placebo-controlled, randomized clinical trial of 0 or 40 mg of atorvastatin/day in 278 individuals scheduled to receive Anthracycline-based chemotherapy for treatment of Stage I-III breast cancer or lymphoma Stage I-IV with a projected > 2 year life expectancy. We will use innovative noninvasive magnetic resonance imaging (MRI) procedures to accurately measure LVEF. In addition, we will measure LV volumes, myocardial strain, fibrosis, aortic pulse wave velocity (PWV) and wall thickness, all factors that can influence LVEF by altering LV pre-load, after-load, and contractility.[19,20] Advanced serum biomarkers will be measured that assess for the presence of oxidative/nitrosative stress, systemic inflammation and circulating neurohormones that also may influence LVEF.

This study will test a new clinical paradigm to manage cancer patients scheduled to receive anthracyclines: primary prevention of Anthracycline-based chemotherapy-related LV dysfunction using pre-treatment with low-cost statins. In addition, this trial will be the first systematic collection of data regarding the mechanism(s) and time course by which LV dysfunction and subsequent CHF evolve in individuals given Anthracycline-based chemotherapy for breast cancer or lymphoma. These data will be useful to physicians trying to determine the optimal cardiac protection strategies when administering chemotherapeutic regimens to their breast cancer or lymphoma patients. The objective of this research is to use inexpensive medications to preserve CV health and thereby improve overall survival in the growing number of breast cancer and lymphoma patients.

Secondary Objectives

Specific Aim 1:

To document the effect of atorvastatin on cognitive function using a battery of neurocognitive tests (HVLT, Rey-Osterreith Figure, COWA, Trail-making Parts A and B, Digit Span and Grooved Pegboard) in breast cancer and lymphoma patients receiving an anthracycline.

Specific Aim 2:

To document the effect of atorvastatin on self-reported quality of life using validated questionnaires (PROMIS including: General form, Cog Concerns, Cog Abilities, Fatigue, Pain intensity and interference, Sleep Disturbance, Physical Functioning and Social Functioning) in breast cancer and lymphoma patients receiving an anthracycline.

2. BACKGROUND

This study is significant in that it addresses:

1) A prevalent clinical problem. CHF and CV events (34,000 annual events in the US) together are the 2nd most common cause of morbidity and mortality in women who receive adjuvant treatment for breast cancer.[21] Administration of Anthracycline-based chemotherapy is a major factor related to development of CHF in this population.[22]
2) A therapeutic intervention to counter the mechanism(s) by which Anthracycline-based chemo therapy injures the heart. To date, no therapies are routinely given to cancer patients to prevent oxidative and nitrosative stress, systemic inflammation, LV replacement fibrosis, and neurohormonal activation–all initiated on receipt of Anthracycline-based chemotherapy. Identifying a therapeutic intervention to address underlying mechanisms of Anthracycline-based chemotherapy-related LV dysfunction would be a significant clinical innovation.
3) The concept of pre-treatment and primary prevention of myocellular injury in cancer survivors. Existing clinical strategies for Anthracycline-based chemotherapy-related CHF are based on post-event clinical management. This study will test a new clinical paradigm**:** primary prevention of Anthracycline-based therapy-related LV dysfunction using low-cost statin pre-treatment.
4) The evolution of CHF and other subclinical CV disease in cancer and lymphoma survivors. The studies planned in this proposal will record the evolution of subclinical processes observed in other large epidemiologic investigations of CHF and CV disease. These data will provide important new information on how cancer or lymphoma survivors develop LV dysfunction that often precedes adverse CV events.
5) CV healthcare expenditures in patients with cancer. Over $800,000,000 per year in the US is spent providing CV-related care for patients experiencing LV dysfunction and CHF after treatment for breast cancer or lymphoma.[3] We will test the effectiveness of a generic, cost-efficient (2 to 5 cents/day), widely available medication that may prevent LV dysfunction in patients receiving Anthracycline-based chemotherapy for cancer.
6) NHLBI and NCI mandates including: a) the NHLBI strategic plan goal to improve understanding of the clinical mechanisms of CV disease and thereby enable better prevention, diagnosis and treatment; and b) the NCI's commitment to determine actionable strategies to reduce the burden of morbidity and mortality in patients with cancer.

In this section, we document the significant background data/information substantiating these statements.

Today, CV events including primarily CHF are the 2nd leading cause of morbidity and mortality in 5-year survivors of breast cancer.[1-7] Because both the number of long-term survivors and the use of complex cancer treatment regimens are increasing, the prevalence of CHF and CV events is growing in all patients receiving Anthracycline-based chemotherapy. The long-term consequences of CHF and CV events after breast cancer and treatment for lymphoma are staggering:

- In 236,000 breast cancer survivors treated and followed between 1975 and 2002, CV event rates increased by 25%, offsetting a 15% decrease in cancer-related mortality.[3,6]
- Using the Surveillance, Epidemiology and End Results (SEER) database of 66- to 80-year-old women (n=7,724) treated for adjuvant breast cancer, the incidence of CHF ranged from 29% to 38% depending on the adjuvant treatment regimen received.[23]
- In a study of 63,566 women with breast cancer aged >65 years, CV disease was the leading cause of death (15.9%) followed by breast cancer (15.1%) in 5-year survivors; and, of those women who died as a result of CV disease, only 25% had existing comorbid CV disease at their breast cancer diagnosis.[23] Similar findings were reported in women below the age of 65 years.[24]
- In 179 breast cancer survivors experiencing a stroke (from a cohort of 11,045), events were 2.5 fold higher in those receiving versus not receiving chemotherapy containing primarily Anthracycline-based chemotherapy.[22,25]

These findings suggest breast cancer survivors with no prior history of CV disease exhibit high rates of CV events, and a relationship exists between breast cancer therapy and survivors' subsequent CV events.

2.1. Anthracycline-based adjuvant therapy's cytotoxic tumor effects also injure cardiomyocytes by promoting peroxynitrite o($ONOO^-$) induced stress.

Anthracyclines interact with the enzyme topoisomerase-IIα and produce double-strand DNA breaks that inhibit tumor cell DNA synthesis, transcription, and replication.[31] This class of agents also generates oxygen- and nitrogen-derived free radicals that are highly cytotoxic for malignant cells.[32]

Generation of oxygen- and nitrogen-derived free radicals can be harmful to cardiomyocytes. In healthy cardiomyocytes, nitric oxide (NO) is beneficial (green, Fig. 1); however, after Anthracycline-based adjuvant therapy, the increased bioavailability of NO and superoxide (O_2^-) promotes preferential production of $ONOO^-$ that induces myocellular injury (red, Fig. 1).[32] Unlike most other organs, the heart has few free radical scavengers, so it is particularly susceptible to injury from free radical production.[32]

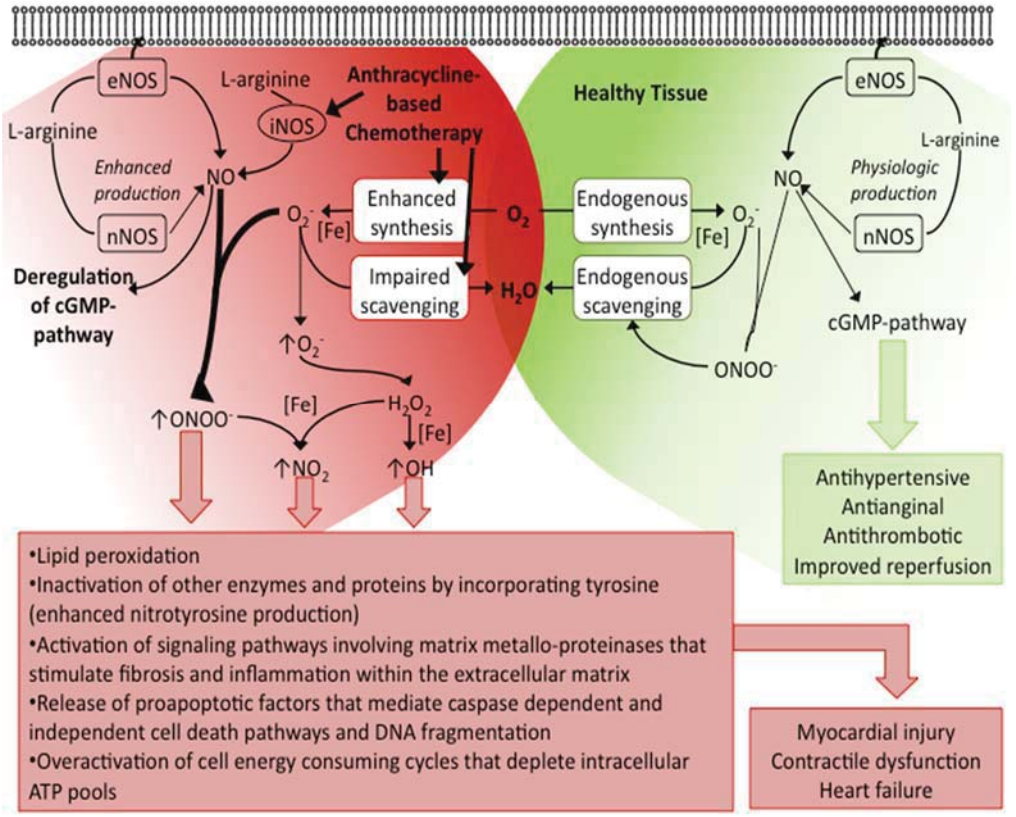

Figure 1: Mechanisms by which anthracycline chemotherapy causes cardiotoxicity. Doxorubicin increases oxidative and nitrosative stress, leading to increased peroxynitrite (ONOO). Peroxynitrite reacts further in both the cytoplasm and the mitochondria, leading to myocardial injury, contractile dysfunction, endothelial dysfunction and heart failure.

Several studies have linked the production of peroxynitrite (Fig. 1) after anthracycline exposure to both cellular myocyte (tension) and global (catheter-based measures of derivatives of pressure and time [dP/dt], LVEF, stroke volume, and cardiac output) abnormalities of LV myocardial performance.[32-34] In C57BL/6J mice receiving single intraperitoneal doses (up to 20 mg/kg) of doxorubicin (DOX, an anthracycline), myocardial apoptosis, levels of inducible nitric oxide synthase (iNOS) expression, mitochondrial superoxide generation, 3-nitrotyrosine (NT) formation (a downstream byproduct of elevated peroxynitrite), myocardial dysfunction, catalase, and glutathione peroxidase were all elevated.[32] These elevations were attenuated by peroxynitrite scavengers.[32] DOX-induced cell death and nitrotyrosine formation were also attenuated by iNOS inhibitors or in iNOS knockout mice.[32] *These studies indicate that Anthracycline-based adjuvant therapy promotes oxidative and nitrosative stress, thus initiating a cascade of events mediated by peroxynitrite formation that injures myocytes and promotes overall LV dysfunction*.

2.2 Statin pre-treatment reduces nitrotyrosine production (related to elevated peroxynitrite formation; red, Figure 1) and subsequent LV dysfunction during and after Anthracycline-based adjuvant therapy.

Five days after a single intraperitoneal injection of DOX at 20 mg/kg, LV function (measured as a change in dP/dt) was reduced in mice receiving DOX alone but not in mice receiving placebo or DOX + fluvastatin (Fig. 2B; red vs. blue and green bars).[14] DOX animals had increased myocellular lipid peroxidation and nitrotyrosine formation (a byproduct of elevated levels of peroxynitrite; Fig. 2A, red bar), increased tumor necrosis alpha (TNFα, a marker of systemic inflammation), and myocellular apoptosis ($p<0.05$ for all).[14] Mice given fluvastatin had lower cardiac expression of nitrotyrosine, an absence of LV dysfunction (Fig. 2B; green

bar), and no increases in markers of systemic inflammation (TNFα) or myocellular apoptosis.[14]

In a recent study of 40 patients scheduled to receive Anthracycline-based adjuvant therapy, those randomized to receive atorvastatin (40 mg per day before and during Anthracycline-based adjuvant therapy) had no decrease in LVEF at 6 months, but those randomized to receive Anthracycline-based adjuvant therapy without statins had an 8% reduction in LVEF (Table 1).[17] In other patients at high risk for CV events not receiving Anthracycline-based adjuvant therapy, pre-treatment with statin therapy before coronary artery interventions or elective surgery reduces subsequent CV events by reducing oxidative stress, inflammatory cytokines, and circulating neurohormones.[38-40] _These data suggest that statin pre-treatment may attenuate LV dysfunction upon receipt of Anthracycline-based adjuvant therapy_.

Table 1: Comparison of Echocardiographic Parameters: Baseline vs. Follow-Up Values in Pilot Study

LVEF (%)	Statin Group (n=20)	Control Group (n=20)	p Value
Baseline	61±8	63±7	
After 6 months	63±9	55±10	
Mean change	+1±4	-8±8	<0.001

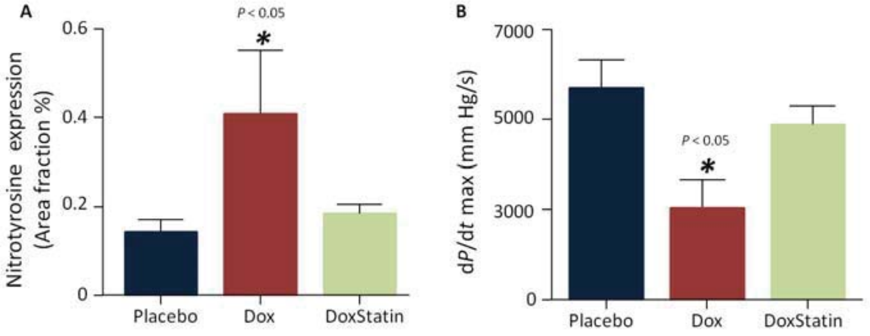

Figure 2: (A; left panel) Cardiac nitrotyrosine expression and **(B; right panel)** systolic LV function in 3 groups treated with placebo, doxorubicin, and a combination of fluvastatin and DOX. *Columns*, mean; *bars*, SE.[14]

2.3. Statins are well-tolerated and do not interfere with cancer therapy response rates or breast cancer recurrence after treatment for stage I-III breast cancer.

Degreef et al. found that statins were highly tolerated and contributed to lowering of adverse serum lipid levels during cancer treatment.[41] Statins plus classical cytotoxic agents in breast cancer cell lines often enhance anti-cancer effects.[42] Statins appear to stimulate apoptotic cell death in breast cancer cells and inhibit cancer cell proliferation by arresting the cell cycle at the G1S phase and inducing apoptosis.[43] Statins did not increase breast cancer recurrence in a population-based prospective cohort study of 18,769 women diagnosed with stage I-III invasive breast carcinoma.[18]

2.4. Preliminary Data

Six months after Anthracycline-based adjuvant therapy, PWV increases and LVEF and myocardial strain diminish.

Aortic stiffness increases upon receipt of anthracycline chemotherapy. J Clin Oncol. 2010;28(1):166-172.[19] Early and persistent evidence of subclinical cardiovascular injury after receipt of anthracycline chemotherapy. Circulation. 2010;122(21) Supplement:A12766[47] Supported by NIH-R33CA12196

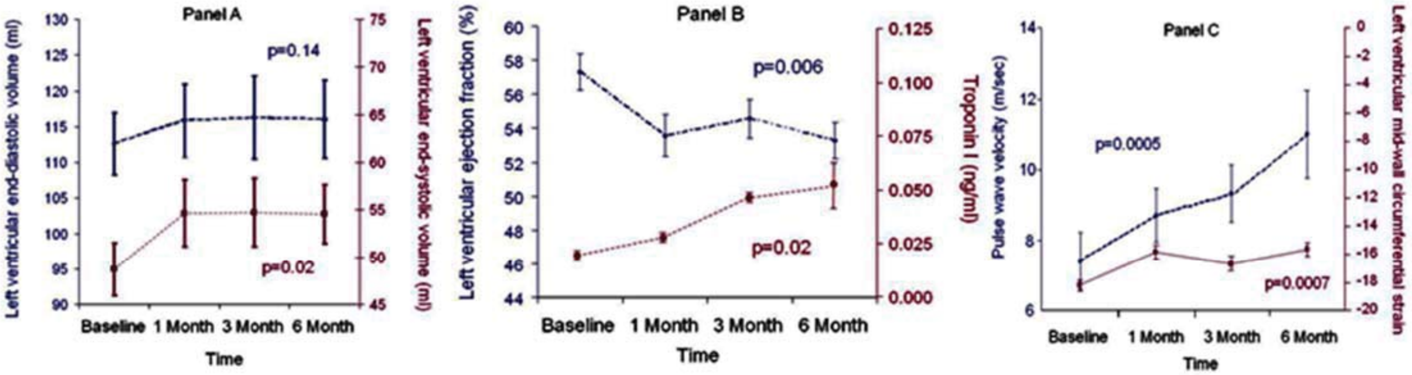

Figure 3: Time-dependent changes in left ventricular (LV) end diastolic volume (left y-axis; panel A) and LV end systolic volume (right y-axis; panel A); in LV ejection fraction (LVEF) (left y-axis; panel B); and mid-wall circumferential strain (right y-axis; panel C). The mean ± the standard error are shown. LV end systolic volumes increase and LVEF and myocardial strain decrease (less negative is a decrease), all indicating a subclinical deterioration in LV systolic performance. These subclinical abnormalities of LV and aortic function are associated with 2-4 fold increases in CV events.[47]

We measured LV volumes, EF and circumferential strain; aortic PWV (a measure of aortic stiffness that contributes to LV after-load; and serum troponin I (TnI); before and 1, 3, and 6 months after initiating 3-month courses of low to moderately dosed Anthracycline-based adjuvant therapy in 51 individuals with breast cancer, leukemia or lymphoma. Participants averaged 52±2 (range 19 to 81) years in age; 65% were women, and 20% were black. LV end systolic volume (LVESV), aortic PWV, and serum TnI increased, and LVEF and circumferential strain decreased after Anthracycline-based adjuvant therapy (Fig. 3, in color). These findings persisted after adjustment for age, gender, race (white-black), doxorubicin (DOX) equivalent chemotherapy dose, CV co-morbidities, cancer diagnosis (breast vs. not breast), and resting cardiac or vascular function (p=0.032 to 0.0001 for all).[47] Change in LV or aortic function parameters was not DOX equivalent dose-related (r=0.01 to 0.05; p=0.85-0.92 for both). In a separate study, we substantiated early decreases in LVEF and increases in PWV after Anthracycline-based adjuvant therapy will be compared to a control population after accounting for age, sex, cardiac output, cancer diagnosis, cardioactive medications, and underlying clinical conditions known to influence PWV, such as hypertension or diabetes (p<0.0001).[19] The 3- to 6-month increase in PWV was similar in effect to aging the vascular system by 15 years. *Cardiac dysfunction occurs early after relatively low to moderate doses of Anthracycline-based adjuvant therapy, and persists 6 months after initiation (3 months after completion) of Anthracycline-based adjuvant therapy*.

In a retrospective study from our institution, statin therapy appears to preserve LVEF after exposure to Anthracycline-based adjuvant therapy. ***Preservation of left ventricular ejection fraction with statins during receipt of anthracycline-based adjuvant therapy. J Am Coll Cardiol 2012;59:E986.[51]*** In 50 participants (33 women, 17 men; aged 48±14 years), we performed blinded MRI measurements of LVEF prior to and 6 months after initiation of Anthracycline-based adjuvant therapy for patients with breast cancer, leukemia, or lymphoma. 14 individuals received a statin for guideline-based indications and 36 did not. Statin therapy was 38±31 mg (range 5-80 mg) atorvastatin (n=6) or simvastatin (n=8). The cumulative anthracycline dose and DOX equivalent doses ranged from 30-450 mg/m^2 with means of 193±95 mg/m^2 and 201±99 mg/m^2 in statin and non-statin users, respectively. At baseline, the LVEF was 57±5% and 57±9% for those taking and not taking a statin, respectively. In a multivariable model accounting for age, gender, co-morbidities (DM, HTN, HLD), and the dose of anthracycline, the LVEF decline after statin use was -0.5% compared to -7.8% in non-statin users (Fig. 4; p=0.0005). Moreover, in those receiving 40-80 mg/day of a statin, LVEF increased 2%; it decreased 3% after low (5-20 mg/day) dose statin (p<0.03).

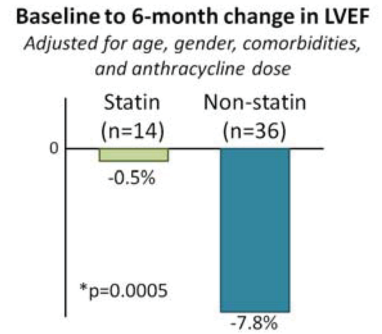

Figure 4: In a multivariable model adjusted for age, gender, co-morbidities, and the anthracycline dose, those who received statin had an LVEF decline of 0.5% compared to the non-statin group 7.8%.

These data indicate that patients on statins for other guideline-based indications may be protected against deterioration in LVEF after Anthracycline-based therapy.

Observational studies suggest that cognitive changes associated with statin use are uncommon and only temporarily affect cognition. Higher doses and advanced age were more associated with the predilection to develop cognitive decline. In our study, a moderate dose of atorvastatin (40 mg) per day will be utilized, and assessments of cognitive functioning across primary domains and cognitive symptoms will be collected using standardized methods from ongoing studies in the NCORPs. Subjective cognitive complaints are reported during chemotherapy by almost 70% of women;[75,84,85] 75% of breast cancer patients evaluated a year after adjuvant chemotherapy performed below age, education, and gender-adjusted means on a variety of neurocognitive tests.[86] Structural changes in cortical and subcortical brain regions have been identified; however, the cause and cognitive correlation are unknown.[87,88] Finally, cognitive deficits associated with LV dysfunction and CVD are well-documented,[89,90] therefore, possible cardio-protective effects of statin therapy on cognition and quality of life, which can be negatively impacted by deterioration in cognition, are important to assess. Based on studies of chemotherapy and cognition, cardio-function and cognition and statin therapy and cognition, we propose a battery of validated neurocognitive measures to evaluate memory (verbal and non-verbal), attention/concentration, working memory, language, psychomotor speed, processing speed, and executive functions, according to procedures utilized currently in the NCORP network by Stephen Rapp, PhD,

one of our co-investigators.[74,75] Additionally, key patient reported outcomes (cognitive symptoms, fatigue, mood, pain, social and physical functioning and sleep) will be assessed with brief, well-validated self-report questionnaires.

3. SUMMARY OF STUDY PLAN

3.1. Overview of Study and Study Design

We propose a double-blind, placebo-controlled randomized clinical trial of 40 mg of atorvastatin (Lipitor®) or placebo per day in 278 men and women age ≥ 21 years old scheduled to receive Anthracycline-based chemotherapy for Stage I-III including inflammatory Breast Cancer, or lymphoma Stage I-IV with a > 2 year life expectancy. Innovative noninvasive MRI measures of LV volumes, EF, myocardial strain, PWV, arterial wall thickness, and other serum markers plus key patient reported outcomes including cognition and quality of life measures will be collected at baseline, 6 and 24 months after initiating treatment with Anthracycline-based therapy. In addition, participants will be contacted by telephone to assess medication compliance/toxicities and Quality of Life at 12 and 18 months. Patients completing at least 24 months but no longer than 27 months of study medication will be followed for an additional 30 days after the last dose of study medication to evaluate any study related adverse events.

3.1.1 COVID-19 - Due to the inability to obtain a final 24 month assessment (related to social distancing because of Coronavirus) of the primary outcome measures for the Prevent study, this study has been amended to provide additional study drug to remaining participants for an additional 3 months. This provision extends the window of opportunity to collect the final study outcome measures within 27 months of beginning the study at a time when social distancing is not required due to the presence of the coronavirus in our communities. This amendment allows for final study related assessments to be accomplished with participants still receiving the study drug. Our statistical analyses which is ongoing to examine the change in outcomes over time allows the exact time between baseline and follow up to be accounted for (I.e. the exact number of days between visits). This modification to allow patients to have their final measurements after 24 months (I.e. 27 months) will not negatively impact our ability to evaluate the stated hypotheses and outcomes for this study. Moreover, this change we will ensure that to the best of our ability we will evaluate the efficacy of the study drug on patients who take the drug until their final outcome assessments, as was the intention of our trial.

3.2. We will use the established NCORP structure of the Wake Forest NCORP Research Base (WF NCORP RB). We will recruit from NCORP Community sites and/or NCI NCORP Research Bases with appropriate MRI facilities that treat modest numbers of breast cancer and lymphoma patients with Anthracycline-based therapy. Importantly, the PI has visited and conducted interviews with all participating NCORP Community sites and/or NCI NCORP Research Bases to verify their MRI and breast cancer and lymphoma recruitment capabilities.

3.3. MRI was selected to provide the endpoints of left ventricular volumes, LVEF, myocardial strain and PWV because of its:

- Reliability (>90% of the valuable subjects in the Jackson Heart Study for which Dr. Hundley serves as the Co-PI of the core lab and the preliminary cancer studies mentioned in Section C.1.),
- Reproducibility (<2% discordance for repeated measures in the pilot data from the cancer participants),
- Accuracy (its ability to detect informative changes indicative of both CV disease and forecasting prognosis),
- Translational capability (its association with large NHLBI efforts to assess CV disease and thereby make additional comparisons), and

- Versatility (in that with a single imaging modality one cannot only collect information regarding the measurement of LVEF but also the factors such as pre-load and after-load that can also impact the measurement of LVEF).

Importantly, all sites have been verified as to the performance of these procedures via onsite interviews and case image recovery.

Ultrasound and radioisotope studies can be used to assess cardiac function. These measures, however, exhibit high variance, are operator-dependent, and are difficult to obtain in individuals with unfavorable body habitus. Radioisotope studies require exposure to ionizing radiation. Catheter-based assessments of left ventricular and vascular structure or function can be acquired but these techniques are associated with the risks of an interventional procedure. Thus, we selected MRI for this study.

4. PARTICIPANT SELECTION

4.1. Inclusion Criteria

4.1.1 Stage I-III breast cancer (including inflammatory and newly diagnosed recurrent breast cancer) or lymphoma Stage I-IV. **(Patients should have** a > 2 year life expectancy)
4.1.2 Scheduled to receive chemotherapy with an Anthracycline (doxorubicin or epirubicin)
4.1.3 ≥ 21 years of age
4.1.4 Patients that are receiving or have received chemotherapy regimens are allowed.
4.1.5 Prior administration of anthracyclines is acceptable if therapy was completed > 6 months prior to study enrollment.
4.1.6 Able to hold breath for 10 seconds
4.1.7 ECOG 0 or 1
4.1.8 See section 4.2.14 for new **exclusion** criteria for breast patients with tissue **expanders.**
4.1.9 Patients with breast **implants** are usually permitted to have an MRI. Check with the MRI technician to confirm.
4.1.10 Participants must have required labs (within 30 days prior to enrollment) as defined below:

It is recommended the Lipid Profile and Glucose be drawn Fasting ≥ 8 hrs.

- X Serum Lipid Profile: Total Cholesterol/HDL/LDL/Triglycerides
  (LDL levels prior to chemotherapy must be ≤ 190 mg/dl)
- X Alanine aminotransferase level (ALT) ≤ 3 x ULN
- X Aspartate aminotransferase level (AST) ≤ 3 x ULN
- X Total bilirubin ≤ 2.0
- X TSH ≤ 1.5 times ULN
- X Creatine kinase ≤ 2.5 x the ULN
- X Glucose < 126 (Diabetics 40–75 years of age are not eligible, See 4.2.2)

If labs are drawn non-fasting and LDL levels are ≥ 190 mg/dl the Lipid Profile should be repeated fasting to determine eligibility. If glucose is ≥ 126 the glucose should be repeated fasting to determine eligibility.

4.2 Exclusion Criteria

4.2.1 Atherosclerotic cardiovascular disease (ASCVD) defined by history of acute coronary syndromes, MI, stable or unstable angina, coronary or other arterial revascularization, stroke, TIA, or peripheral arterial disease presumed to be of atherosclerotic origin.
4.2.2 40 to 75 years of age with diabetes per ACC/AHA 2013 Guidelines
4.2.3 Significant ventricular arrhythmias (>20 PVCs/min due to gating difficulty) atrial fibrillation with uncontrolled ventricular response
4.2.4 Current use of statin therapy
4.2.5 Current or history of hepatic dysfunction
4.2.6 Uncontrolled hypothyroidism
4.2.7 Recent extended history of constant-recurrent substance abuse or another medical condition that might compromise safety or the successful completion of the study.
4.2.8 Patients with ferromagnetic cerebral aneurysm clips or other intraorbital/intracranial metal; pacemakers, defibrillators, functioning neurostimulator devices or other implanted electronic devices.

4.2.9 Current use of the following CYP 3A4 inhibitors are not allowed: Boceprevir, Clarithromycin, Cyclosporine (oral), Darunavir plus ritonavir, Fosamprenavir, Fosamprenavir plus ritonavir, Gemfibrozil, Grapefruit juice >1 liter per day, Itraconazole, Lopinavir plus ritonavir, Nelfinavir, Saquinavir plus ritonavir, Telaprevir, Tipranavir plus ritonavir

4.2.10 Current use of Rifampin and Digoxin
4.2.11 Unable to provide informed consent
4.2.12 Symptomatic Claustrophobia
4.2.13 Pregnant or breast feeding. Due to unknown risks and potential harm to the unborn fetus a negative serum pregnancy test within 10 days prior to enrollment is required in women with child-bearing potential. For this reason women of child-bearing potential must agree to use adequate contraception (hormonal or barrier method of birth control; abstinence (not having sex), oral contraceptives, intrauterine device (IUD), DeProvera, tubal ligation, or vasectomy of the partner (with confirmed negative sperm counts) in a monogamous relationship (same partner). An acceptable, although less reliable method involves the careful use of condoms and spermicidal foam or gel and/or cervical cap or sponge prior to study entry and for the duration of study participation. Should a woman become pregnant or suspect she is pregnant while participating in this study, she should inform her study physician immediately.

Due to the potential effect of **atorvastatin** when excreted in breast milk, women who are currently breast-feeding are not eligible for this study.

4.2.14 Breast patients with tissue **expanders** are ***not*** allowed with the exception of tissue expanders made of material that are MRI compatible. Check with the MRI technician to confirm.

4.3. Inclusion of Women and Minorities

Table 2: Race/Ethnicity

**Gender**	White	Black or African-American	Hispanic or Latino	Asian or Pacific Islander	Unknown	Total
Male	30	8	0	0	0	38
Female	193	43	2	2	0	240
Total	223	51	2	2	0	278

4.4. Recruitment and Retention Plan

4.4.1 Over 600 breast and lymphoma patients should be eligible for enrollment among the NCORP sites, approximately 14% of whom will be males. Assuming conservatively that 17% (1 in 6) of the patients will consent to the study, we expect ~100 patients to be enrolled annually once the study is active at all planned sites. We will enroll 278 patients. We anticipate 28% of our cohort will drop out, so we expect 200 individuals to complete the study. In addition to NCORP sites, study enrollment will be opened to Alliance site members in which study coordinators, imaging expertise, and patient populations are readily available.

4.4.2. At each site, specific recruitment plans may include the following according to the site's institutional policy: screening clinic charts; tumor registry data; referral sources such as patient advocate groups, retirement communities, churches, support organizations, community organizations, newspapers, radio; or patient recruitment posters and recruitment letters.

4.4.3. The research PI or designee at each WF NCORP Research Base NCORP, which may include the clinic physician, resident, research nurse or research assistant, will review cancer registry and medical chart information to identify patients eligible for this protocol. Patients identified using these methods will be asked to join the study during their next clinic visit/consultation.

Accrual is expected to be 8 patients per month. Targeted accrual should be met in approximately 3 years for the 5 year study. A maximum of 278 patients will be enrolled on this trial. Patients will be followed for at least 24 months. Patients completing at least 24 months of study medication but no longer than 27 months will be followed for an additional 30 days after the last dose of study medication to evaluate any study related adverse events. After the Final assessment, the patient is no longer followed and data is no longer collected.

5. AGENT ADMINISTRATION

5.1. Dose Regimen and Dose Groups:

Group 1 (one 40 mg atorvastatin tablet each morning by mouth for at least 24 months but no longer than 27 months) and Group 2 (one placebo tablet each morning by mouth for at least 24 months but no longer than 27 months).

5.2. Atorvastatin/Placebo Administration

Patients will be 1:1 randomized to receive the study agent, (one 40 mg atorvastatin tablet daily by mouth for at least 24 months but no longer than 27 months) or a placebo (one tablet daily by mouth for at least 24 months but no longer than 27 months). The study agent or placebo should be started within 48 hours (2 days) prior to first chemotherapy treatment. The patient must receive 2 doses prior to first chemotherapy treatment. When possible those 2 doses may be given at least 12 hours apart. If necessary, due to time constraints, the patient may be given two tablets at the same time at least 90 minutes prior to first chemotherapy treatment.[98]

Either agent will be taken once any time of day, with or without food, 7 days per week, on an outpatient basis, for a total of at least 24 months but no longer than 27 months. The atorvastatin pills and matching placebo pills used for this study will be provided free of charge to the patient.

5.3. Run-in Procedures. N/A

5.4 Contraindications

Active liver disease, which may include unexplained persistent elevations in hepatic transaminase levels, or hypersensitivity to any component of this medication.

5.5. Concomitant Medications

Atorvastatin is metabolized by cytochrome P450 3A4. Concomitant administration of this statin with inhibitors of CYP 3A4 can lead to increases in plasma concentrations of atorvastatin. The extent of interaction and potentiation of effects depend on the variability of effect on CYP 3A4. The following CYP 3A4 inhibitors are not allowed: Boceprevir, Clarithromycin, Cyclosporine (oral), Darunavir plus ritonavir, Fosamprenavir, Fosamprenavir plus ritonavir, Gemfibrozil, Grapefruit juice >1 liter per day, Itraconazole, Lopinavir plus ritonavir, Nelfinavir, Saquinavir plus ritonavir, Telaprevir, Tipranavir plus ritonavir. It is also recommended due to possible reactions to not use Rifampin and Digoxin.

5.6 Medication Modification

5.6.1 Medication Modification for Myalgias

Grade 1 Myalgias – If needed patient may take NSAIDs/analgesics at the discretion of the treating physician. Frequently this type of skeletal pain is not relieved with these medications.

Grade 2 or 3 Myalgias - Study medication should be held and patient instructed in the use of NSAIDS/analgesics per treating physician orders. ALT, AST and CK should be drawn within 24 hours.

- If labs are in acceptable range (ALT and AST are < 3 x ULN and CK < 2.5 x ULN) study medication should be resumed.
- If Grade 2 or 3 myalgias redevelop, study medication should be discontinued. Patient will be considered off study medication but will continue to be followed.
- If ALT and/or AST are >3 x ULN and/or CK >2.5 x ULN study medication should be discontinued. Patient will be considered off study medication but will continue to be followed.

Any patient with suspected rhabdomyolysis/compartment syndrome of lower leg or rupture of tendon should stop the study medication immediately. The treating physician should assess the patient and order CK isoenzymes and/or other labs as deemed necessary. Patient will remain on study (off study medication) and be followed as usual.

5.6.2 **Medication Modification for Elevated ALT, AST and/or CK Labs**

5.6.2.1 **Elevated baseline ALT, AST and/or CK**

At one and three months (+ or – 7 days) additional labs will be drawn on patients with a baseline AST/ALT between ULN and ≤ 3 x ULN and CK between ULN and ≤ 2.5 x ULN. Labs are only repeated if baseline labs were above normal but within eligibility limits.

5.6.2.2 Elevated ALT, AST and/or CK while taking study medication

If AST, ALT is > 3 x ULN and/or CK is > 2.5 x ULN while patient is taking study medication lab should be repeated in 1 week. If still elevated in 1 week, study medication should be stopped and AST, ALT, CK repeated in 6 weeks.

At 6 weeks the site PI/treating physician should review labs and patients clinical status. If labs are within eligibility range patient may be re-challenged with study medication. The decision regarding re-challenging the patient is at the site PI/treating physician's discretion.

5.6.2.3 General Medication Modification

Contact Robin Rosdhal RN OCN at (336) 713-6519 for any medication related issues. This includes but is not limited to the patient experiencing difficulty with their medication regimen due to chemotherapy treatment and delays or interruptions in taking study medication.

5.7 Adherence

5.7.1 Adherence will be estimated as: 1) the proportion of expected pills taken while on treatment and 2) the proportion of the total number of pills that could be taken if the participant completed the study. We will calculate and report the mean adherence across all individuals as well as the proportion of patients who were 75% adherent (using both definitions of adherence). The primary analyses will include all randomized participants, regardless of adherence. Secondary analyses will be done using participants who were at least 75% adherent, separately using each definition.

5.7.2 To determine patients' adherence with the study agent, a medication diary will be utilized. It will be given to the patient by staff with instructions on how to complete. Every other month, medication diaries will be collected to verify medication usage. In addition, at 6 and 24

months any remaining bottle(s) of study agent will be collected from the patient and a pill count will be performed to verify the patient-recorded information in their medication diary.

6. PHARMACEUTICAL INFORMATION

6.1 Atorvastatin (Lipitor): The dosing for this study will be a daily dose of 40mg for at least 24 months but no longer than 27 months. Atorvastatin can be administered as a single dose at any time of the day, with or without food. Pharmaceutical tests of atorvastatin suggests aggressive dosing of HMG-CoA reductase inhibitors (statins) reduce lipid levels and cardiovascular mortality and morbidity to a significantly greater extent than standard statin doses, according to the PROVE IT and REVERSAL trials. However, in our study, dosage has been set at 40mg daily in consideration of cancer chemotherapy treatment.

Pharmacological Description: Atorvastatin is a synthetic lipid-lowering agent. Atorvastatin is an inhibitor of 3-hydroxy-3-methylglutaryl-coenzyme A (HMG-CoA) reductase. This enzyme catalyzes the conversion of HMG-CoA to mevalonate, an early and rate-limiting step in cholesterol biosynthesis.

Atorvastatin calcium is [R-(R*, R*)]-2-(4-fluorophenyl)-ß, δ-dihydroxy-5-(1-methylethyl)-3-phenyl-4-[(phenylamino)carbonyl]-1Hpyrrole-1-heptanoic acid, calcium salt (2:1) trihydrate. The empirical formula of atorvastatin calcium is (C33H34 FN2O5)2Ca•3H2O and its molecular weight is 1209.42. Its structural formula is:

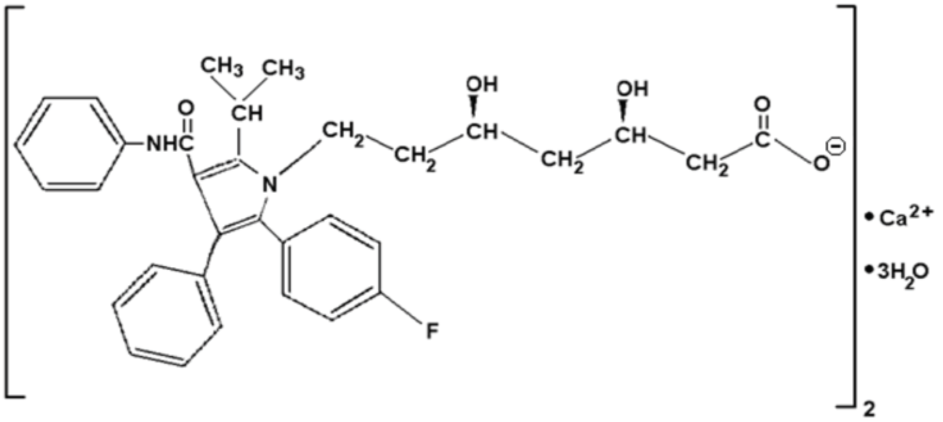

Atorvastatin calcium is a white to off-white crystalline powder that is insoluble in aqueous solutions of pH 4 and below. Atorvastatin calcium is very slightly soluble in distilled water, pH 7.4 phosphate buffer, and acetonitrile; slightly soluble in ethanol; and freely soluble in methanol. Lipitor tablets for oral administration contain 10, 20, 40, or 80 mg atorvastatin and the following inactive ingredients: calcium carbonate, USP; candelilla wax, FCC; croscarmellose sodium, NF; hydroxypropyl cellulose, NF; lactose monohydrate, NF; magnesium stearate, NF; microcrystalline cellulose, NF; Opadry White YS-1-7040 (hypromellose, polyethylene glycol, talc, titanium dioxide); polysorbate 80, NF; simethicone emulsion.

ProHance® (Gadoteridol) Injection is a nonionic contrast medium for magnetic resonance imaging (MRI), available in vials for intravenous injection into either a vein or currently placed central line (port-a-cath) to help identify structures within the body during a MRI scan.

The ProHance® injection dose is 0.15 mmol/kg, infused at 1 ml/sec, followed by a saline flush of 20 ml. If the IV gauge is small, the infusion rate may be decreased and is not critical.

6.2 Reported Adverse Events and Potential Risks

Atorvastatin

Most Common Events

- **Gastrointestinal:** Abdominal pain (In pre-marketing and open extension studies with a median follow-up period of 18 months, abdominal pain was reported in 7.3% of enrolled patients [n=2423]), constipation (In pre-marketing and open extension studies with a median follow-up period of 18 months, constipation was reported in 6.6% of enrolled patients [n=2423]), nausea (In pre-marketing and open extension studies with a median follow-up period of 18 months, nausea was reported in 5.4% of enrolled patients [n=2423])
- **Neurologic:** Headache (In pre-marketing and open extension studies with a median follow-up period of 18 months, headache was reported in 7.4% of all patients enrolled [n=2423])
- **Respiratory:** Upper respiratory infection (9%)
- **Muscle Pain (Myalgia):** Soreness usually in upper arms and shoulders or hip and thighs about 6%.

Less Common

- Confusion < 2%

Rare but Serious Events

- **Hepatic:** Cholestatic hepatitis, increased liver enzymes (1%)
- **Musculoskeletal:** Compartment syndrome of lower leg, disorder of muscle (20 to 40 mg/day, 0.02% to 0.08%; 80 mg/day, 0.53% to 0.9%), rhabdomyolysis, rupture of tendon
- **Endocrine:** Diabetes < 0.25% or 1 per 400.

ProHance®

Most Common Events

- headache, nausea, vomiting, itching, watery eyes, skin rash, facial and/or tongue swelling,
- wheezing, chest tightness, or troubled breathing, hypotension (a decrease in blood pressure) may occur in about 2% of patients.

Less Common

- Extravasation happens in one out of every 250 to 500 procedures. <0.2 to 0.4%

Rare but Serious Events

- renal failure
- nephrogenic systemic fibrosis (NSF)
- confusion

The MRI contrast agent is used to help identify structures within the body on a MRI scan. Minor reactions {principally headache, nausea, vomiting, itching, watery eyes, skin rash, facial swelling, thickening of the tongue, wheezing, chest tightness, or troubled breathing, hypotension (a decrease in blood pressure)} may occur in about 2% of patients. Serious or life-threatening reactions have been reported in about 1 in 400,000 patients who receive these agents. All standard of care, medications and monitoring will be provided during this MRI scan. You may still participate in this study if a) you cannot receive Gadolinium-contrast because you have known allergies to this contrast or have other severe drug allergies or b) you are not allergic to gadolinium-contrast but choose not to receive this contrast due the risks associated with the procedure.

- Sometimes contrast may leak out of the IV and into the fatty tissue around a vein which is called an extravasation (x TRAH vuh SAY shun). This happens in one out of every 250 to 500 procedures. Nonionic contrast is not likely to cause tissue damage and the contrast is reabsorbed. If extravasation occurs, MRI staff will manage according to established protocols at Wake Forest Baptist Health.

- In very rare circumstances associated with dialysis and complete renal failure, gadolinium in very high doses has been reported to be associated with nephrogenic systemic fibrosis (NSF), a condition that causes hardening of tissue under the skin and potentially death. To minimize the risk of developing this disorder, this study complies with all FDA criteria for administration of gadolinium. Dialysis patients are excluded from receipt of gadolinium contrast as part of this study. As a consequence, an estimation of your kidney function obtained within a month of your MRI scans will be used to identify potential risk to study participation as defined by the FDA. Those with a glomerular filtration rate <60 ml/min will not receive contrast as part of this study.

- Recent medical literature publications have reported that patients receiving gadolinium contrast may accumulate gadolinium in parts of the brain long after the last administration. Currently available information has not identified any adverse health effects resulting from this accumulation of gadolinium based contrast agents. Participants will be notified of any new information related to gadolinium-based contrast as it becomes available.

MRI Sedation

In the event a patient is claustrophobic (extreme discomfort or fear of small spaces) they may be offered an intravenous injection of a sedative medication to make the patient drowsy, relaxed and comfortable during the MRI scan. This is at the discretion of the site physician. Because sedatives may decrease alertness and cause lightheadedness or dizziness, the patient must have another adult drive them home from the clinic if they are given sedation.

Possible risks and side effects of frequently used sedative medications may include:

Most Common Events may occur in about 2% of patients.

- dizziness
- headache

Less Common

- unusually fast/slow/irregular heartbeat,
- fainting,
- confusion,
- mental/mood changes,
- trouble breathing,
- muscle twitching and involuntary movements,

Rare but Serious Events

- throat discomfort,
- skin rash and hives.

.

6.3 Drug Availability and Description

Atorvastatin will be manufactured and supplied by Greenstone. The study agent will be directly supplied to Biologics, Inc. who will fill pill bottles and create labeled prescriptions.

Biologics, Inc. will provide procurement, over encapsulation and placebo manufacture services.

Atorvastatin - supplied as 40mg capsules packaged in 90-count bottles. Biologics will procure the active supply from wholesaler for use in this study. The active supply will then be over encapsulated to

maintain the blind of the study. Each bottle of over encapsulated Atorvastatin will be 180-ct. Biologics anticipates making two purchases of active supply, one prior to the start of the study and one at year two, to accommodate the five year length of the trial and expirations of the product.

Prior to study launch, Biologics will procure and over encapsulate approximately 54,720 tablets.
At year two, Biologics will procure and over encapsulate approximately 54,720 tablets to complete the study.

Placebo - A placebo is a substance, like a sugar pill, that is not thought to have any effect on the disease or condition. Placebos are used in research studies to see if the drug being studied really does have an effect.

Matching placebo supplied as capsules packaged in 90-count bottles. Biologics will facilitate the manufacture of a matching placebo to maintain the blind of the study. Each bottle of matching Placebo will be 180-ct. Biologics anticipates the manufacture of two batches of the matching placebo, one prior to the start of the study and one at year two, to accommodate the five year length of the trial and expirations of the product.

Prior to study launch, Biologics will facilitate the manufacture of approximately 54,720 placebo capsules. At year two, Biologics will facilitate the manufacture of approximately 54,720 placebo capsules to complete the study.

Shipment 1
12 Month Patient Specific Supply
- Biologics will ship 2 bottles of study drug to the study site
- Months 1-12: 2 bottles (180-ct)

At approximately month 11, Biologics will contact the site to prepare the subsequent shipment of study drug for the patient to complete months 13-24.

Shipment 2
12 Month Patient Specific Supply
- Biologics will ship 2 bottles of study drug to the study site
- Months 13-24: 2 bottles (180-ct)

Shipment 3 (if applicable)
12 Month Patient Specific Supply
- Biologics will ship 1 bottle (180 ct) of study drug to the patient if needed.
- Patient will return all unused study drug to the site upon final assessment.

ProHance® - supplied and shipped to each individual site according to their site specific research agreement with Bracco Diagnostics Inc. MRI may be performed without contrast until all arrangements for administering contrast at a site has been established. For more information, please contact Kimberly Lane at klane@wakehealth.edu.

6.4 Agent Ordering and Distribution

Biologics, Inc. will provide with atorvastatin 40mg pills and matching placebo and will distribute study drug/placebo directly to participating sites (WF exempt). The contact information for Biologics, Inc. is:

Biologics Clinical Research Services
120 Weston Oaks Court
Cary, NC 27513
(1-800) 693-4906
clinicaltrials@biologicsinc.com

6.5. Agent Accountability

Atorvastatin or placebo pills
Each participating NCORP and Alliance site will maintain a careful record of the inventory and disposition of the study agents received (atorvastatin or placebo pills) using the NCI Drug Accountability Record Form (DARF). This will include adequate records of receipt, dispensing, quantities, dates and final disposition of study agent.

ProHance®: Each participating MRI NCORP site will maintain a careful record of the inventory and disposition of the ProHance® received using the NCI Drug Accountability Record Form (DARF) or drug accountability form used at site. This will include adequate records of receipt, dispensing, quantities, dates and final disposition of the ProHance®. All remaining and or expired ProHance® must be disposed of according to site institutional policy. The site is responsible for maintaining the drug accountability record.

Each participating MRI NCORP site will maintain a careful record of the inventory and disposition of any sedative medication given to the patient according to institutional policy.

6.6 Packaging and Labels

Summary
The study will remain open approximately 5 years or until all 278 patients have been accrued and can complete the study. Biologics, Inc. will send to the participating site the 24-month supply for each patient randomized to the study, except for those patients who may be extending to within 27 months from the start of study For these patients, Biologics may ship directly to patients.

Packaging
- All study drugs will include a patient specific label adhered to the bottles. Biologics will place the bottles in a Ziploc bag and include a patient specific label on the outside. Biologics will also include the study number on the label to avoid confusion at the sites.
- Each shipment includes a label on the Ziploc bags with the following information (as needed):
- The Study Number
- The Patient study ID number
- The Patient's name or initials
- The number of capsules
- Administration instructions: Take one tablet daily by mouth Drug identification (Atorvastatin 40mg OR Placebo)
- Expiration Date
- Dosing instructions (Administer as Directed per Protocol)
- Storage instructions ("Store at controlled room temperature, 20-25°C (68-77°F)")
- Date dispensed
- IND caution statement and/or local regulatory statements
- Emergency contact instructions

For all drug shipments from Biologics, Inc., a packing slip will be enclosed that includes the date and quantity of drug provided patient name/initials, study ID number, drug identification and expiration date.

Biologics, Inc. will process and ship "same day" of patient randomization if received before 5:00 p.m. EST Monday through Friday. Orders received after 5:00 pm EST Monday through Friday will be processed and shipped the next business morning.

All drug orders are shipped via *FedEx for Priority Overnight* delivery. Study drug is shipped in boxes designed to maintain temperature stability.

Once study drug is received at the clinical trial site, the designated site coordinator validates contents of package match information provided on packing slip with study medication received. The inventory and disposition of atorvastatin or placebo pills must be recorded using the NCI Drug Accountability Record Form (DARF).

ProHance® will be supplied by Bracco Diagnostics Inc. ProHance® is not dispensed as a patient specific medication; it is shipped in bulk to each individual site with appropriate manufacturing labeling and directions.

6.7. Storage

Atorvastatin pills are supplied as 40mg pills in 90 count bottles and stored at 20-25°C (68-77°F). ProHance® *will be* stored according to Bracco Diagnostics Inc. package insert.

6.8. Registration Process

All patient enrollments for Wake and non-Wake sites will go through the Wake Forest NCORP Research Base (see Section 6.8.2). NCORP sites can request membership through the Wake Forest NCORP Research Base.

6.8.1 Non-Wake sites can register through CTSU.

CTSU Registration Procedures for Wake and non-Wake NCORP Sites:

Cancer Therapy Evaluation Program Investigator Registration Procedures

Food and Drug Administration (FDA) regulations and National Cancer Institute (NCI) policy require all individuals contributing to NCI-sponsored trials to register and to renew their registration annually. To register, all individuals must obtain a Cancer Therapy Evaluation Program (CTEP) Identity and Access Management (IAM) account (https://ctepcore.nci.nih.gov/iam). In addition, persons with a registration type of Investigator (IVR), Non-Physician Investigator (NPIVR), or Associate Plus (AP) (i.e., clinical site staff requiring write access to OPEN, RAVE, or acting as a primary site contact) must complete their annual registration using CTEP's web-based Registration and Credential Repository (RCR) (https://ctepcore.nci.nih.gov/rcr).

RCR utilizes five person registration types.

- IVR — MD, DO, or international equivalent;
- NPIVR — advanced practice providers (e.g., NP or PA) or graduate level researchers (e.g., PhD);
- AP — clinical site staff (e.g., RN or CRA) with data entry access to CTSU applications (e.g., Roster Update Management System (RUMS), OPEN, Rave,);
- Associate (A) — other clinical site staff involved in the conduct of NCI-sponsored trials; and
- Associate Basic (AB) — individuals (e.g., pharmaceutical company employees) with limited access to NCI-supported systems.

RCR requires the following registration documents:

Documentation Required	IVR	NPIVR	AP	A	AB
FDA Form 1572	✓	✓			
Financial Disclosure Form	✓	✓	✓		
NCI Biosketch (education, training, employment, license, and certification)	✓	✓	✓		
GCP training	✓	✓	✓		
Agent Shipment Form (if applicable)	✓				
CV (optional)	✓	✓	✓		

An active CTEP-IAM user account and appropriate RCR registration is required to access all CTEP and Cancer Trials Support Unit (CTSU) websites and applications. In addition, IVRs and NPIVRs must list all clinical practice sites and IRBs covering their practice sites on the FDA Form 1572 in RCR to allow the following:

- Addition to a site roster;
- Assign the treating, credit, consenting, or drug shipment (IVR only) tasks in OPEN;
- Act as the site-protocol Principal Investigator PI on the IRB approval; and
- Assign the Clinical Investigator (CI) role on the Delegation of Tasks Log (DTL).

In addition, all investigators act as the Site-Protocol PI, consenting/treating/drug shipment, or as the CI on the DTL must be rostered at the enrolling site with a participating organization (i.e., Alliance).

Additional information can be found on the CTEP website at https://ctep.cancer.gov/investigatorResources/default.htm. For questions, please contact the **RCR Help Desk** by email at RCRHelpDesk@nih.gov.

Cancer Trials Support Unit Registration Procedures:

This study is supported by the NCI CTSU

IRB Approval:

For CTEP and Division of Cancer Prevention (DCP) studies open to the National Clinical Trials Network (NCTN) and NCI Community Oncology Research Program (NCORP) Research Bases after March 1, 2019, all U.S.-based sites must be members of the NCI Central Institutional Review Board (NCI CIRB). In addition, U.S.-based sites must accept the NCI CIRB review to activate new studies at the site after March 1, 2019. Local IRB review will continue to be accepted for studies that are not reviewed by the CIRB, or if the study was previously open at the site under the local IRB. International sites should continue to submit Research Ethics Board (REB) approval to the CTSU Regulatory Office following country-specific regulations.

Sites using their local IRB or REB, must submit their approval to the CTSU Regulatory Office using the Regulatory Submission Portal located in the Regulatory section of the CTSU website. Acceptable documentation of local IRB/REB approval includes:

- Local IRB documentation;
- IRB-signed CTSU IRB Certification Form; and/or

- Protocol of Human Subjects Assurance Identification/IRB Certification/Declaration of Exemption Form.

In addition, the Site-Protocol Principal Investigator (PI) (i.e. the investigator on the IRB/REB approval) must meet the following criteria to complete processing of the IRB/REB approval record:

- Holds an Active CTEP status;
- Rostered at the site on the IRB/REB approval (*applies to US and Canadian sites only*) and on at least one participating roster;
- If using NCI CIRB, rostered on the NCI CIRB Signatory record;
- Includes the IRB number of the IRB providing approval in the Form FDA 1572 in the RCR profile; and
- Holds the appropriate CTEP registration type for the protocol.

Additional Requirements

Additional requirements to obtain an approved site registration status include:

- An active Federal Wide Assurance (FWA) number.
- An active roster affiliation with the Lead Protocol Organization (LPO) or a Participating Organization (PO); and
- Compliance with all protocol-specific requirements (PSRs).

Downloading Site Registration Documents:

Download the site registration forms from the protocol-specific page located on the CTSU members' website. Permission to view and download this protocol and its supporting documents is restricted based on person and site roster assignment. To participate, the institution and its associated investigators and staff must be associated with the LPO or a PO on the protocol.

- Log on to the CTSU members' website https://www.ctsu.org using your CTEP-IAM username and password;
- Click on the Protocols tab in the upper left of your screen
  - Enter the protocol number in the search field at the top of the protocol tree, or
  - Click on the By Lead Organization folder to expand, then select [Corresponding Organization], and protocol number [NCI Protocol #];

Protocol Specific Requirements for WF- 98213 Site Registration:

- CTSU IRB Certification (for sites not participating via the NCI CIRB)
- CTSU IRB/Regulatory Approval Transmittal Sheet (for sites not participating via the NCI CIRB)
- ***See Section 8 for protocol specific training requirements. This must be completed prior to enrollment.***

Submitting Regulatory Documents:

Submit required forms and documents to the CTSU Regulatory Office via the Regulatory Submission Portal on the CTSU website.

To access the Regulatory Submission Portal log on to the CTSU members' website → Regulatory →Regulatory Submission

Institutions with patients waiting that are unable to use the Regulatory Submission Portal should alert the CTSU Regulatory Office immediately at 1-866-651-2878 in order to receive further instruction and support.

Checking Your Site's Registration Status:

You can verify your site's registration status on the members' side of the CTSU website.

- Log on to the CTSU members' website;
- Click on *Regulatory* at the top of your screen;
- Click on *Site Registration;*
- Enter your 5-character CTEP Institution Code and click on Go.

Note: The status shown only reflects institutional compliance with site registration requirements as outlined above. It does not reflect compliance with protocol requirements for individuals participating on the protocol or the enrolling investigator's status with the NCI or their affiliated networks.

6.8.2 Online Patient Enrollment Procedures for Sites (Wake and non-Wake sites)

Do Not Enroll Patients Through "OPEN"

IRB letter of approval and an IRB approved consent form must be received by the Research Base Protocol Information Office – Attn: Site Coordinator prior to patient enrollment. Fax: (336) 716-6275. An "Eligibility Checklist / Enrollment Form" will be used to complete the on-line enrollment.

Online Enrollment
Log on to the Wake Forest NCORP Research Base enrollment web site at https://ccrbis.phs.wakehealth.edu. Enter your user name and password (which may be obtained by contacting Robin Rosdhal, RN at rosdhal@wakehealth.edu In the 'Patient Enrollment and Protocol Information' table, click the 'Register Patient/Patient Info', with the corresponding protocol number found in the drop down box to the right. Fill in the eligibility criteria forms using the drop down boxes. If further information is needed by Biologics or Data Management, they will contact you. Once the patient information has been entered online print a copy of the eligibility checklist/enrollment form for your records. Press the submit button, a confirmation page will appear. Print this confirmation sheet for your records. The Wake Forest NCORP On-line Protocol Enrollment/Eligibility form, initial flow sheet, signed consent, should be faxed to 336-713-6476 or mailed to Data Management:

Wake Forest NCORP Research Base
Data Management Center
Wake Forest Baptist Medical Center
Building 525@Vine, 4th floor
Medical Center Boulevard
Winston-Salem, NC 27157

These forms should be retained in the patient's study file. These forms will be evaluated during an institutional NCI/Wake Forest NCORP Research Base site member audit.

If you have questions related to the enrollment process or require assistance with enrollment, please contact the Wake Forest NCORP Research Base DMC between 8:30am and 4:00pm EST, Monday through Friday at (336) 713-3172 or 713-6507.

6.8.3 **Randomization Process**
See Section 12.1

6.9. Unblinding Methods

In the event a patient on this study develops a toxicity (adverse event or severe adverse event) for which the patient's physician or other health care professional feels that it is in the patient's best interest to know what drug they are taking (active study drug or placebo), the following procedure should be followed:

- Step 1: The patient's physician or a designated health care professional should call the Wake Forest University Baptist Medical Center Physician Access Line at (336) 716-7654 and ask that Dr. Glenn Lesser, Director of the WF NCORP Research Base, be contacted immediately either in his office, by pager, or at home. In the event Dr. Lesser cannot be reached, the PAL operator should contact Dr. Kate Weaver, Director, Symptom Treatment Protocols, in her office, by pager, or at home. If neither Dr. Lesser nor Dr. Weaver can be reached, the PAL operator should contact Dr. David Herrington, Co-Investigator, in his office, by pager, or at home. If Dr. Lesser, Dr. Weaver or Dr. Herrington cannot be reached, the PAL operator should contact Dr. Joseph Yeboah, Co-Investigator, in his office, by pager, or at home.

- Step 2: Once contact has been made, the patient's physician or health care professional should explain the reason for the request to unblind the treatment arm that the patient is on. If the Research Base representative feels that the toxicity (AE/SAE) is possibly, probably or definitely related to the study drug, then the next step will be followed.

- Step 3: The responsible Research Base representative will call the pharmacist @ Biologics, Inc. (phone: 1-800-850-4306). There is an "on-call" service provided 24 hours a day, seven days a week for the Chemical Drug Trials unblinding service. The Biologics pharmacist may contact the patients' physician and/or health care professional directly with the unblinding information. Written documentations of the unblinding process will be sent to the Research Base Principal Investigator by Biologics, Inc.

- Step 4: In the event that the patient's treatment is unblinded, that patient will be taken off study with no further study follow-up. Appropriate procedures for grading toxicities, assigning causality, and reporting severe adverse events (if applicable), should be followed. The event will be reviewed by the WF NCORP Safety and Toxicity Review Committee and reviewed by the NCORP Research Base Data Safety and Monitoring Board.

  Record the details of the adverse event and/or unblinding in the site source documentation and complete appropriate AE forms.

Unblinding Study Participants at Study Completion

Study participants may be unblinded at the conclusion of the study if **all** patient specific data for the requesting site are completed and submitted to the Data Management Center (DMC) and all of the primary study outcomes have been determined and the final data set is cleaned and prepared for analysis. Site members can obtain unblinding information by sending an email request to the WF NCORP Administrator or Data Management Supervisor with a list of PID #s. After confirming with the DMC that patient specific data for all patients at the requesting site have been received, completed and entered into the RB database, the Research Pharmacy will be notified. An email from the Research Pharmacy containing the unblinding information will be sent directly to the requesting site.

6.10. Agent Destruction/Disposal

Unused drug should be destroyed on site following site institutional policies and procedures.

7. CLINICAL EVALUATIONS AND PROCEDURES

7.1 Schedule of Events

At each participating Research Base site, medical charts will be screened to determine potential eligibility by physicians (including residents or fellows, if applicable), research nurses, or clinical research associates. This information should be recorded on the Screening Form. Patients identified as potentially eligible will then be asked to consider joining the study. Patients meeting initial eligibility criteria and who agree to participate in the study will sign informed consent and then undergo a baseline nurse exam, laboratory tests and quality of life tests. At baseline, 6, and 24 months (or within 27 months), the entire battery of neurocognitive tests and questionnaires will be administered by a trained and certified examiner. At 12 and 18 months participants will be contacted by phone to assess toxicities and medication compliance. Quality of Life Questionnaires will be completed via mail. Patients will be instructed in the self-administration of the study agent. Patients will take one pill daily beginning within 48 hours (2 days) prior to the start of chemotherapy. It may be helpful if the patient receives 2 doses prior to first chemotherapy treatment and those 2 doses be at least 12 hours apart. If necessary however due to time constraints, the patient may be given 2 tablets at the same time at least 90 minutes prior to first chemotherapy treatment. Potential side effects and risks are described in Section 6.2. Adverse event reporting is described in Section 10. Study outcomes will be assessed at baseline, after 6 months and at the end of 24 month (or within 27 months). Patients completing at least 24 months of study medication but no more than 27 months will be followed for an additional 30 days after the last dose of study medication to evaluate any study related adverse events.

7.2. Screening/ Baseline Evaluations

Due to the scheduling complexity of protocol requirements prior to the start of the first chemotherapy treatment, screening and baseline procedures and randomization may be performed at the sites discretion once the patient has signed the consent. The patient should be registered within 30 days of screening labs.

Subjects will be checked for height, weight, waist circumference, heart rate and blood pressure. Body Mass Index (BMI) will be calculated at the data center. Patient physical exam including peripheral edema, smoking status and family history will be collected by study personnel.

Waist circumference Measuring Directions

- Patient should be instructed to not hold their breath.
- The measuring tape should be secure but not tight.
- The area being measured can be found by placing the hands on the waist and moving them downward until the top curve of the hip bone can be felt.
- The area being measured can be found by placing the hands on the waist and moving them downward until the top curve of the hip bone can be felt.
- Place the tape measure around the top of the hip curve and wrap around at the level of the belly button. (This may not be the same level as the top of the pants.)
- Record this measurement in inches on the flow sheet.

Study personnel will download the CV Risk Calculator according to specifications in Appendix 3. After collecting the appropriate information, it will be entered in the CV Risk Calculator. Note: If the patient is < 40 years old, data cannot be entered in CV Risk Calculator. The result will be documented on the flow sheet and the Physician Statin Update Letter to be mailed to the treating and primary care physician. **If the study participant should have statin therapy initiated, the study coordinator should complete the Open Label Form (Appendix 12) and submit to the Data Management Center.**

Serum and blood derived biomarkers - A blood sample of approximately 4 tablespoons will be taken from a vein in the arm/central line for lab analysis.

Statins exert pleiotropic effects (modulation of systemic inflammation and the neurohormonal axis) that in other models of heart failure influence LV function,[57,58] and have been modulated in pilot animal studies.[14] In this study, we will determine the relationship between Anthracycline-based therapy administration, statin use and biomarker evidence of systemic inflammation[59] and neurohormonal axis activation.[60] We will associate these relationships with changes in both LV and vascular function.

Labs for future testing - Participants who consent to have blood stored for future research will have an additional 1 tablespoon of blood drawn. These labs will be picked up and processed by LabCorp. Blood samples will be shipped and stored with a unique identifier and will not include any information protected by HIPAA regulations. LabCorp's Bio-repository for blood samples is located in Kannapolis, North Carolina.

MRI Variables - MRI exams will be accomplished according to previously published techniques described on the Research Base website under "Helpful Guides" located in the Appendices section. Our research group and Dr. Hundley (PI) has extensive experience developing and utilizing these techniques in single center or multi-center efforts including the Jackson Heart Study (Dr. Hundley is Co-PI for the core lab) and the MESA study.

LVEF is the primary outcome because it is the most widely clinically implemented assessment of LV performance, and is used to guide therapeutic intervention. LVEF has limitations as it can vary dependent on LV pre-load or after-load. To account for these limitations we are utilizing MRI to measure LVEDV (pre-load) and PWV (after-load). Also, we will perform advanced assessments of LV contractility including myocardial strain which is utilized in other NHLBI studies (such as MESA). This will allow us to measure systolic performance independent of LV pre-load and enable cross. Aortic distensibility will be assessed as an exploratory aim.

The MRI is for study purposes only, the imaging results will not be reviewed at the site. The WF NCORP Research Base will send a clinical update when the MRI is reviewed and/or alert the site study staff if an unrecognized myocardial infarction was identified.

At baseline, if a routine standard of care ECHO or MUGA is obtained and available at the time of enrollment, the LVEF result should be documented on the enrollment form. If the LVEF result is available after enrollment, record the result on the flow sheet. .

An MRI should be obtained between 5 and 7 ½ months. All anthracycline therapy should be completed and tissue expanders (if applicable) removed before obtaining the cardiac MRI. For time periods outside of these ranges, special permission for extenuating circumstances (e.g., significant healthcare issue warranting hospitalization) may be obtained by direct contact with Dr. Hundley, the study PI. If it is determined a patient cannot have a 6 month (5-7 $^{1/2}$ month) MRI, the patient will continue on study as per protocol.

The 24 – 27 month MRI should be obtained within 30 days prior to the last dose of study medication. For time periods outside of these ranges, special permission for extenuating circumstances (e.g., significant healthcare issue warranting hospitalization) may be obtained by direct contact with Dr. Hundley, the study PI.

Due to unknown risks and potential harm to the unborn fetus and because some methods of birth control are not 100% reliable, it is suggested that women of childbearing potential take a pregnancy test prior to each MRI exam, as written in the informed consent form. It is also suggested that a

negative serum or urine pregnancy test be performed within 10 days of each MRI exam. Each site should follow their institution's policies.

MRI may be performed without contrast but it is preferred contrast be used to better define the cardiac structures. If the site has a signed contract with Bracco and ProHance is supplied, the site will be responsible for maintaining a careful record of the inventory and disposition of the ProHance® received.

Neurocognitive Function

Cognition and Quality of Life

Once eligibility has been established the neurocognitive test battery and the study questionnaires will be completed. Individual components of the full battery are described below. Tests have been selected to represent a range of cognitive abilities which have been reported in the literature to be affected by chemotherapy including attention, verbal memory, working memory, executive functions, speed of mental processing and verbal fluency. An abbreviated battery consisting of two tests has been developed for the baseline assessment to reduce participant burden (see below). All cognitive testing will be performed by a trained and certified research assistant blinded to treatment assignment. The WF NCORP Research Base has conducted training workshops and produced videos for administering this cognitive test battery for our completed trials among irradiated brain tumor patients and breast cancer patients exposed to chemotherapy, and maintains a stringent certification process (Section 8.2).

An abbreviated battery of the cognitive tests and shorter versions of quality of life questionnaires will be used at baseline to reduce participant burden and due to time constraints related to the start of the first chemotherapy treatment. The shorter battery includes the same tests in the longer version used at 6 and between 24 - 27 months to ensure we have adequate consistency across assessment points. Each cognitive test has adequate psychometric properties and has been used in cancer research including large national and international clinical trials (Ahles & Saykin, 2002; Mehta et al., 2002; Meyers et al., 2004). The entire cognitive test battery takes 10 minutes to administer at baseline and 30 minutes to administer at 6 months and 24 months..It will be administered with the questionnaires which takes 10 minutes at baseline 6 months and 24 months.

Controlled Oral Word Association Test (COWA)

The COWA (Benton & Hamsher, 1976) measures speed of mental processing, verbal fluency, and executive function. Subjects are asked to name as many words as possible all beginning with a specified letter. A total of three trials are administered, each with a different letter (C-F-L). The score on the COWA is the total number of words named across the three trials minus repetitions. The COWA has two equivalent forms (C-F-L and P-R-W) that will reduce practice effects. Internal consistency reliability (alpha=0.83) and test-retest reliability (r = 0.74) are excellent (Ruff et al., 1996).

Hopkins Verbal Learning Test-Revised (HVLT-R)

The HVLT-R measures verbal learning and memory. It consists of a 12-item word list which is read to subjects on three successive learning trials. Free recall scores are recorded for each learning trial. After a 20-minute interval during which subjects complete other non-interfering tasks and questionnaires they are asked to recall the target words. Lastly, a yes/no recognition task is then presented in which subjects are asked to identify all target words by responding "yes," and to reject 12 non-target words by responding "no." The HVLT has six equivalent alternate forms (Brandt, 1991) to minimize confounding by practice effects. Test-retest reliability of the HVLT is quite good (0.74). The test is brief, taking only 10 minutes to administer, and it is well-tolerated by compromised (geriatric and dementia) populations. Scores for immediate recall (total of three trials), delayed recall (total number of words recalled after 20 minutes), and recognition (total number of words correctly identified) will be the variables derived from the HVLT-R.

Trail Making Test, Parts A & B (TMT-A, TMT-B)
Part A of the TMT(Reitan, 1958) measures attention and visual motor skills and processing speed and requires subjects to connect 25 numbered circles in the proper sequence (1-2-3-…) as quickly as possible. TMT-B is similar except subjects are required to connect dots in an alternating numerical and alphabetical sequence (1-A-2-B-…). TMT-B with its added complexity and set shifting requirements is a widely used measure of executive function. The score for TMT-A and TMT-B is the total time in seconds required to complete the task. Scores can also be generated for number of errors and number of circles correctly connected. The TMT has excellent reliability and validity (Reitan, 1992).

Digit Span Test (DST)
The DST (Wechsler, 1981) assesses attention and working memory. It requires respondents to repeat back gradually increasing spans of numbers. Seven series of two spans of each length are presented and repeated backwards. A total score is the sum of the longest span backwards.

Self-Report Questionnaires:
The Patient Reported Outcomes Measurement Information System (PROMIS) is a collection of well-validated, brief, fee-free instruments assessing key patient reported outcomes related to this study. Each PROMIS scale has excellent psychometric properties (reliability and validity) and are being used in many clinical trials and observational studies. For this study, we will include the short forms for:

PROMIS Applied Cognition – General Concerns – Short Form 4a (4 items)

PROMIS Applied Cognition – Abilities – Short Form 4a (4 items)

PROMIS Fatigue – Short Form 4a (4 items)

PROMIS Mood – Emotional Distress - Anger – Short Form 5a (5 items)
Emotional Distress - Anxiety – Short Form 4a (4 items)
Emotional Distress - Depression – Short Form 4a (4 items)

PROMIS Pain Intensity - Short Form 3a (3 items)

PROMIS Pain Interference - Short Form 4a (4 items)

PROMIS Sleep Disturbance - Short Form 4a (4 items)

PROMIS Physical Function - Short Form 4a (4 items)

PROMIS Ability to Participate in Social Roles and Activities - Short Form 4a (4 items)

The total administration time for these questionnaires is 10 minutes.

7.3. Evaluations During Study Intervention

Study participants in both statin and placebo groups will have a Cardiac MRI (see Section 7.2 for pregnancy testing prior to MRI exams), and blood drawn for a cardio profile, lipids, CK, ALT, AST, Total Bilirubin, glucose, TSH, Hematocrit (only if gadolinium contrast is administered for the MRI) and neurohormonal biomarkers at screening/baseline, 6 months and between 24 - 27 months. Biomarkers studied include ultrasensitive troponin I (for myocellular injury); C-reactive protein, interleuken-6, tumor necrosis factor-a (systemic inflammation); and renin and aldosterone (circulating neurohormones). Additional blood samples will be drawn on participants who consented to have blood stored for future research.

See section 5.6.1 for Modification of Study Medication related to Myalgias which may require additional labs at 1 and 3 months.

See section 5.6.2 for Modification of Study Medication related to Elevated ALT, AST and CK Labs.

Neurocognitive test battery and Quality of Life Questionnaires will be obtained. At 12 and 18 months participants will be contacted by phone to assess toxicities and medication compliance. Quality of Life Questionnaires will be completed via mail.

7.4. Evaluations at Completion of Study Intervention

All study participants will have an Cardiac MRI (see Section 7.2 for pregnancy testing prior to MRI exams) and blood drawn for a cardio profile, Lipids, CK, ALT, AST, Total Bilirubin, glucose, TSH, Hematocrit (only if gadolinium contrast is administered for the MRI procedure) and neurohormonal biomarkers noted in 7.3, between 24 – 27 months. Additional blood samples will be drawn on participants who consented to have blood stored for future research

Neurocognitive testing and Quality of Life Questionnaires will be obtained. Follow-up assessments will include laboratory evaluations, pill counts, and structured interviews assessing outcomes and potential adverse events.

7.5. Post-intervention Follow-up Period.

Patients completing 24 months of study medication will be followed for an additional 30 days after the last dose of study medication to evaluate any related adverse events. After the 25 month contact, the patient is no longer followed and data is no longer collected from the patient.

7.6 Methods for Clinical Procedures

Processes for obtaining the MRI and lab sample data are provided in Appendices 11 and 12

MRI invoices should be emailed to Teresa Crotts at tcrotts@wakehealth.edu or mailed to:

Attn: Teresa Crotts
Wake Forest Baptist Health
Department of Internal Medicine
Section on Cardiology
Medical Center Boulevard
Winston-Salem, NC 27157

7.7. Study Parameters Table

Study agent or placebo should be started when received. The patient must receive 2 doses prior to first chemotherapy tx. and those 2 doses may be given at least 12 hours apart. If necessary, due to time constraints, patient may be given 2 tablets at the same time at least 90 minutes prior to first chemotherapy tx. All evaluations/procedures are to be completed within + or – 30 days of specified time unless indicated otherwise. ***Note: 24 month MRI should be obtained within 30 days prior to the last dose of study medication.***

**Evaluation Procedure**	**Screening** (C)	**Baseline** (D)	**1 & 3 Mths** (G)	**6 Mths**	**12 Mths**	**18 Mths**	**between 24 - 27 Mths**	**1 Mth Post Final Assess ment**
Informed Consent	X							
Demographics (p. 6 of enrollment form)	X							
Nurse Exam		X		X			X	
Cardiac/Vascular MRI (A)(B)		X (A1)(B)		X (A2)(B)			X (A3)(B)	
Screening labs (C)	X							
CV Risk Calculator		X						
General Labs (D) (E)		X		X			X	
Biomarker Labs (F)		X		X			X	
Future Research Lab (if pt. consents)		X		X			X	
ALT/AST and CK - Above normal at baseline (G)			X					
Unscheduled ALT/AST/ CK for myalgias/ elevated levels (See Section 5.6)								
Baseline Booklet (H)		X						
6 Month Booklet (H)				X				
12 Month Booklet (H)					X			
18 Month Booklet (H)						X		
24 Month Booklet (H)							X	
Flow Sheet/Addenda		X	X	X	X	X	X	
Phone Contact Form					X	X		
MRI Encounter Form (I)		X		X			X	
Current Med Form		X		X	X	X	X	
Medication Diaries & pill count (J)		Every other month						
Chemo Form	Submit this form after each course (not cycle) of chemotherapy							
RT Form	To be completed when last RT tx. is received or indicating patient will not receive RT.							
Open Label Form	If placed on a statin by their physician. (See Appendix 12 Statin Physician Update)							
Early Withdrawal Treatment/Consent	Submit if patient withdraws from active tx, or withdraws consent prior to end of .study.							

Screening Form	Submit this form every month.	
1 Month Post Contact Form (K)	For patients completing between 24 -27 months of study med – Submit this form 30 days after last dose of study medication.	X

A1 - Baseline MRI's are required. If a routine standard of care LVEF is obtained by ECHO or MUGA this result should be documented on the flow sheet or the enrollment form.

A2 - 6 month MRI should be obtained between 5 and 7 ½ months once anthracycline therapy has been completed and tissue expanders (if applicable) have been removed. For time periods outside of these ranges, special permission for extenuating circumstances (e.g., significant healthcare issue warranting hospitalization) may be obtained by direct contact with Dr. Hundley, the study PI. If it is determined a patient cannot have a 6 month (5-7 1/2 month) MRI, the patient will continue on study as per protocol.

A3 - 24 – 27 month MRI should be obtained within 30 days prior to the last dose of study medication. It is important to recognize that this sample point is very important for the conduct of the study. For scans that need to be performed outside of this range of time, special permission for extenuating circumstances (e.g., significant healthcare issue warranting hospitalization) may be obtained by direct contact with Dr. Hundley, the study PI.

B - It is suggested that a negative serum or urine pregnancy test be performed within 10 days of each MRI exam for all women of childbearing potential.

C - Screening labs must be within 30 days prior to enrollment. Screening CMP results should be used for Baseline CMP (ALT, AST, Glucose, Total Bili), Lipid Panel, Creatine Kinase, TSH -- Serum HCG must be drawn within 10 days prior to enrollment in women of child-bearing potential

D - Baseline evaluations must be completed prior to start of chemo.

E - Hematocrit - Obtain only when gadolinium contrast is administered for the Cardiac MRI.

F - Biomarkers (C-reactive Protein, Troponin-I, Aldosterone, Interleukin-6, TNFα, Renin)

G - Repeat labs at 1 and 3 months should be collected within + or **-** 7 days if baseline values are above normal but within eligibility limits.

H - Booklets for the additional cognitive and QOL questionnaires are described in Section 11.1.

I - Submit MRI Encounter Form on all patients. If MRI not performed, indicate this on MRI Encounter Form and submit to the Wake Forest NCORP Research Base.

J - For both blinded and open label patients, medication diaries should be collected in-person or by mail every other month. A pill count is required when the patient is seen at 6 months, 24 months or at any time the patient discontinues the study medication.

K - 1 Month Post Final Assessment contact form - Patients completing 24 - 27 months of study medication should be followed for an additional 30 days after last dose of study medication for related adverse events.

7.8. Off Treatment Criteria

Participants may stop taking atorvastatin for the following reasons: completed the protocol-prescribed intervention, adverse event or serious adverse event, inadequate agent supply, concomitant medications, medical contraindication, or interruption of chemotherapy due to adverse events or death.

Participants will continue to be followed, if possible, for safety reasons and in order to collect endpoint data according to the schedule of events. The study is designed to account for drop-outs due to poor health or death or loss to follow-up; thus, there will be no replacement of subjects that must drop out of the study after enrollment.

7.9 Off-Study Criteria

Participants may go 'off-study' for the following reasons: the protocol intervention and any protocol-required follow-up period is completed, adverse event/serious adverse event, lost to follow-up, concomitant medication, medical contraindication, withdraw consent, or death.

Since the study inception and development of the inclusion/exclusion criteria, guidelines regarding the potential administration of statins have been released by the ACC/AHA on November 12, 2013. These new guidelines suggest the use of a calculator to assess cardiovascular risk with subsequent initiation of statin therapy based on that risk. Concomitant with the release of the guidelines, however, have been several articles suggesting that the calibration of the calculator was somewhat imperfect, particularly in regards to data from both the Nurses' Health and Physicians' Health studies.

As a result of these controversies, the study investigators elected to leave the initial entry and exclusion criteria intact. Importantly however, their respective primary care physician will be mailed a letter raising their awareness to the publication of these guidelines. If the study participant and their primary care physician elect to initiate statin therapy, they will be counseled to stop their study drug and notify the respective investigators at their study site. They will still be allowed to participate in all other aspects of the trial as an open label statin user. Data analyses regarding intention to treat as well as treatment effect will be performed.

8. PROTOCOL SPECIFIC TRAINING REQUIREMENTS

8.1 MRI Training

- Refer to the “Helpful Guides” located on the Research Base website in the Appendices section.
- The study PI presented the MRI protocol for review and discussion with the MRI technologists and associated study coordinators during the Investigator meeting held at the 2013 Annual Research Base NCORP Meeting.
  Prior to the start of participant enrollment into the study, Wake Forest University Medical Center cardiology imaging specialists observe and continue MRI training at each of the study locations.

Each site will be required to submit 2 satisfactorily performed studies to the image coordinating center at Wake Forest before initiating the study.

8.2 NEUROCOGNITIVE TRAINING

8.2.1 Certification Requirement

Certification for the administration of the neurocognitive battery and questionnaires will include self-study, internet-based training, didactic presentations, role-played administrations, and Q&A and feedback. All training will be supervised by experienced test administrators (Dr. Rapp at WFUSM, Robin Rosdhal RN OCN, Site Coordinator or other trained/certified administrator who will be responsible for certifying test administrators.) They will also be responsible for helping staff maintain certification by having regular meetings to discuss the procedures and providing supplemental training as needed.

Bilingual staff must be certified to administer neurocognitive battery to non-English speaking patients. Certification procedure must be completed by staff prior to patient enrollment.

8.2.2 Certification Procedures

All neurocognitive assessments will be conducted by trained and certified research personnel. Training and certification procedures must be completed by research staff prior to patient enrollment. Certification for administration of the neurocognitive battery will include viewing of a training video on the Wake Forest University Health Sciences website, reviewing the content of the protocol-specific test booklets including all aspects of administration and scoring, a didactic presentation of each test and questionnaire to be administered, and role-playing of administrations with Q&A and feedback. All training will be supervised by experienced test administrators (Dr. Stephen Rapp, psychologist and co-PI of this study, and other trained/certified administrators designated by the WF NCORP Research Base.)

There are 3 ways to become certified: 1) Training and certification on-site by WF NCORP certified personnel, contact NCORP@wakehealth.edu; 2) Training and certification at the annual meeting of the WF NCORP Research Base, held each fall (contact the Research Base Administrative Secretary at (336) 716-0891 for date of next meeting; 3) By sending in a recorded presentation and administration booklet to NCORP@wakehealth.edu for

certification review. Alliance sites that have current credentialing for Alliance neurocognitive studies may choose #3 for WF certification.

Periodic re-certifications will be required if it has been more than 6 months since the staff member administered the tests. This procedure has been successfully used in our studies of cognitive effects of cancer and its treatments as well as by Dr. Rapp and his team in large scale, multi-site clinical trials and observational studies (e.g., WHIMS, CoSTAR, SPRINT, Look AHEAD, LIFE, MESA).

9. SPECIMEN MANAGEMENT

9.1 Storage of Blood for Future Research Testing (if applicable)

At baseline, 6 months and 2 years an additional 2 tubes of blood will be collected on patients who have given consent to store blood for future research testing.

These future tests may include searches for combinations of genetic nucleotide polymorphisms that are associated with susceptibility to a fall in LVEF in patients receiving chemotherapy. At present, planned testing is not specified and thus not included in our specific aims. All blood samples will be de-identified and stored in LabCorp's Bio-repository located in Greenfield, IN. Only researchers approved by Dr. Greg Hundley, the principal investigator at Wake Forest School of Medicine will receive the sample. Lab supplies and instructions for blood stored for future research testing will be provided by LabCorp. (See Appendix 10 for further lab instructions).

Labs for future research testing should not be drawn and sent to LabCorp until specific consent for future research testing has been verified and eligibility established.

9.2 Lab Corp: Routine Labs required Per Protocol

Lab Corp will be utilized for all labs**.** LabCorp couriers will deploy to each site from local LabCorp facilties for specimen pick-up as samples are drawn. All specimens should be collected in containers provided by LabCorp. Note storage times and temperature instructions for each test.

Eligibility and baseline labs (Cholesterol, LDL, HDL, Triglycerides, Glucose, Creatine Kinase, TSH, ALT, AST, Total Bilirubin, Hematocrit [only if gadolinium contrast is to be administered for the MRI procedure] and BHCG if applicable) will be drawn and analyzed by LabCorp. Once eligibility has been determined the remaining protocol labs (Hematocrit preferred within 8 hours but acceptable to within 40 hours of MRI for patient's receiving gadolinium contrast) CRP, Troponin 1, Aldosterone, IL-6, TNF-a, and Renin will be drawn and sent to LabCorp.

9.3 Collection and Handling Procedures

Refer to Appendix 10 for instructions.

10. REPORTING ADVERSE EVENTS

- A list of serious adverse events that have occurred or might occur that are related to this study intervention can be found in Section 6.2.
- Serious Adverse Event reporting begins after the informed consent is signed.
- Serious Adverse Events occurring within 30 days of study completion must be reported via FDA Form 3500 (MedWatch).

10.1 Protocol Specific Reporting for Serious Adverse Events (SAEs)

DEFINITION: ICH Guideline E2A and Fed. Reg. 62, Oct. 7, 1997 define serious adverse events as those events which meet any of the following criteria?

- Results in death

- Is life threatening (Note: the term life-threatening refers to an event in which the patient was at risk of death at the time of the event; it does not refer to an event which hypothetically might have caused death if it were more severe).
- Requires inpatient hospitalization or prolongation of existing hospitalization
- Results in persistent or significant incapacity or substantial disruption of the ability to conduct normal life functions.
- Is a congenital abnormality/birth defect
- Events that may not meet these criteria, but which the investigator finds very unusual and/or potentially serious, will also be reported in the same manner.

- **Grades 4 and 5 expected (solicited) and unexpected (unsolicited) SAEs that meet the above definition for SAEs and/or regardless of attribution (i.e. regardless of whether they are related to this study intervention or not) should be reported to the RB DMC using the FDA Form 3500 (MedWatch).**

- Site staff and/or Principal Investigators will report to the RB Data Management Staff within 24 hours of discovering the details of all unexpected severe, life-threatening (grade 4) and/or fatal adverse events (grade 5) if there is reasonable suspicion that the event was definitely, probably, or possibly related to the study intervention.

Otherwise, the MedWatch should be sent to the RB DMC by fax or email within 10 working days of discovering the details of the SAE.

Data Elements to include on the MedWatch are:
- SAE reported date
- CTCAE Term (v4.03)
- Event onset date and event ended date
- Severity grade (use table provided in Section 10.1.3 below)
- Attribution to study intervention (relatedness)
- Action taken with the study participant and intervention
- Outcome of the event
- Comments

10.2 Guidelines to determine grade and severity of AEs and/or SAEs

Identify the adverse event using the NCI Common Terminology Criteria for Adverse Events (CTCAE) version 4.03. The CTCAE provides descriptive terminology and a grading scale for each adverse event listed. A copy of the CTCAE can be found at http://ctep.cancer.gov/protocolDevelopment/electronic_applications/ctc.htm#ctc_40

AEs will be assessed according to the CTCAE grade associated with the AE term. AEs that do not have a corresponding CTCAE term will be assessed according to the general guidelines for grading used in the CTCAE v4.03. as stated below.

Grade	Severity	Description
1	Mild	Mild; asymptomatic or mild symptoms; clinical or diagnostic observations only; intervention not indicated.
2	Moderate	Moderate; minimal, local or noninvasive intervention indicated; limiting age-appropriate instrumental ADL*.
3	Severe	Severe or medically significant but not immediately life-threatening;<br>Hospitalization or prolongation of hospitalization indicated; disabling; limiting self-care ADL**.

4	Life threatening	Life-threatening consequences; urgent intervention indicated.
5	Fatal	Death related to AE.

Activities of Daily Living (ADL)

* Instrumental ADL refer to preparing meals, shopping for groceries or clothes, using the telephone, managing money, etc.

** Self-care ADL refer to bathing, dressing and undressing, feeding self, using the toilet, taking medications, and not bedridden.

The Research Base Grant PI, Safety and Toxicity Review Committee and/or Study Chair will take appropriate action to inform the membership and statistical personnel of any protocol modifications and/or precautionary measures, if this is warranted.

The RB DMC is responsible for communicating AEs/SAEs to the FDA, the drug sponsor, WF IRB, the WF Safety and Toxicity Review Committee (STRC) and/or other regulatory agencies as appropriate per agency reporting requirements.

Institutions must comply with their individual Institutional Review Board (IRB) policy regarding submission of documentation of adverse events. All MedWatch reports should be sent to the local IRB in accordance with the local IRB policies.

10.3 Follow-up of SAEs

Site staff should send follow-up reports as requested when additional information is available. Additional information should be entered on the MedWatch form in the appropriate format. Follow-up information should be sent to the RB Data Management Center as soon as available.

SAEs (Grade 4 and/or Grade 5) for this protocol should be followed for those related to the study intervention. Documentation should include:

- PID
- Date of SAE
- Description of the event
- Relationship of the SAE to the study intervention
- Severity
- Intervention/Resolution

10.4 Adverse Event Posting

Adverse event summaries are posted on the WF RB website each month. New MedWatches are emailed to sites the 1St week of each month and posted on the password protected CCRBIS.

11. STUDY MONITORING

11.1 Data Management Schedule

The Eligibility checklist/Enrollment Form should be completed on-line prior to placing the patient on study. Data forms will be submitted to the WF NCORP Research Base. See Section 6.8.2 for mailing address, or fax to (336) 713-6476 according to the timetable below:

Form	Submission Schedule
Consent Form, MRI Encounter Form, Baseline Neurocognitive Booklet, Flow Sheet/Addenda, Current Medication Form	Baseline within 14 days of enrollment
MRI Encounter Form, 6 month Neurocognitive Booklet, Flow Sheet/Addenda, Current Medication Form	6 months within 14 days of assessment
Flow Sheet/Addenda	1 and 3 months within 14 days of assessment
Radiation Treatment Form	Complete within 14 days of pt. receiving last RT tx. or indicating patient will not receive RT.
12 month Neurocognitive Booklet, Flow Sheet/Addenda, Telephone Contact Form, Current Medication Form	12 months within 14 days of assessment
18 month Neurocognitive Booklet, Flow Sheet/Addenda, Telephone Contact, Current Medication Form	18 months within 14 days of assessment
MRI Encounter Form, 24 month Neurocognitive Booklet, Flow Sheet/Addenda, Current Medication Form	24 months within 14 days of assessment
Medication Diaries	Every other month within 14 days of completion
Early Withdrawal Form	Participants who withdraw from treatment **or** study prior to completion. Submit within 14 days of withdrawal.
Open Label Form (See page 2 Physician Update)	If placed on a statin by their physician within 14 days of occurrence.
Chemotherapy Treatment Form	Within 14 days of completion. of each course (not cycle)
Screening Form	Every month within 14 days of completion
1 Month Post Final Assessment Contact Form (For patients completing at least 24 months of study medication but no more than 27 months)	Complete this form 30 days after last dose of study medication. Submit within 14 days of assessment.

11.2 Case Report Forms

Participant data will be collected using protocol-specific case report forms (CRF).

11.3 Source Documents

Source documents are the original signed and dated records of participant information (e.g., the medical record, shadow chart) which may include electronic documents containing all the information related to a participant's protocol participation. Source documents are used to verify the integrity of the study data, to verify participant eligibility, and to verify that mandatory protocol procedures were followed. An investigator and other designated staff are required to prepare and maintain adequate and accurate documentation that records all observations and other data pertinent to the investigation for each individual participating in the study. All data recorded in the research record (including data recorded on CRFs) must originate in the participant's medical record, study record, or other official document sources.

Source documents substantiate CRF information. All participant case records (e.g., flow sheets, clinical records, physician notes, correspondence) must adhere to the following standards:

- Clearly labeled in accordance with HIPAA practices so that they can be associated with a particular participant or PID;
- Legibly written in ink;
- Signed and dated in a real time basis by health care practitioner evaluating or treating the participant;
- Correction liquid or tape must not be used in source documents or on CRFs.
- Corrections are made by drawing a single line through the error. Do not obliterate the original entry. Insert the correct information, initial, and date the entry.

All laboratory reports and imaging study and scans must have:

- Complete identifying information (name and address of the organization performing, analyzing, and/or reporting the results of the test); and
- Range of normal values for each result listed.

11.4 Data and Safety Monitoring Board

The Data Safety Monitoring Board meets every six months to review all phase II and phase III protocols. The Board includes members demonstrating experience and expertise in oncology, biological sciences, biostatistics and ethics. The DSMB report is generated by the RB statistician. Areas of review may include the following: Date study Opened; Study Objectives; Patient Accrual; Patient Status and Retention; Study Status; Last Contact Status; Patient Compliance; Number of Biopsies/Labs as needed; Patient Characteristics; Summary of Observed Toxicities; Adverse Events; Date, Event briefly described, Relationship to Drug, Arm assigned; Summary of Primary and Secondary Measures. Following each meeting, WF RB will provide DSMB reports to all accruing sites via the regular weekly email correspondence.

11.5 Record retention

Clinical records for all participants, including CRFs, all source documentation (containing evidence to study eligibility, history and physical findings, laboratory data, results of consultations, etc.), as well as IRB records and other regulatory documentation will be retained by the Investigator in a secure storage facility in compliance with HIPAA, OHRP, FDA regulations and guidance, and NCI/DCP requirements unless the standard at the site is more stringent.

11.6 CDUS Reporting

The WF NCORP Research Base Data Management Center will submit quarterly reports to DCP/CTEP by electronic means using the Clinical Data Update System (CDUS)

12. STATISTICAL CONSIDERATIONS

12.1. Study Design/Endpoints

This is a multi-center, double-blind, placebo-controlled clinical trial that randomizes 278 participants into 1 of 2 treatment arms: Group 1 (one 40 mg atorvastatin tablet each morning by mouth for 24 months) or Group 2 (one placebo tablet each morning by mouth for at least 24 months but no more than 27 months) with the goal of having at least 200 remaining at the trial's end for final assessments (assuming an approximate 28% loss to follow-up). A permuted block randomization stratified by cancer type (breast vs lymphoma) and **planned** Anthracycline-based therapy doses of Doxorubicin < or ≥ 240 mg/m^2 or Epirubucin < or ≥ 400mg/ m^2; close to the median dose of our pilot studies) will ensure treatment group balance is achieved within strata. As in all our pilot studies and clinical practice, Doxorubicin and Epirubicin doses will be assessed to account for receipt of different anthracyclines. The primary endpoint in this trial is LVEF, and thus the primary efficacy analysis will examine whether the statin therapy can attenuate the reduction in LVEF in patients with breast cancer or lymphoma who receive Anthracycline-based therapy. Primary endpoints include LVESV, LV end diastolic volume (LVEDV), LV mid-wall circumferential strain, T1-ECV (a measure of replacement fibrosis), PWV, aortic wall thickness, CRP and TNF-α, serum lipoprotein levels, and circulating neurohormones.

12.2. Primary Aims

Specific Aim 1: To determine if atorvastatin administration preserves LVEF 24 – 27 months after receipt of Anthracycline-based therapy for breast cancer and lymphoma.

Specific Aim 2: To determine if baseline to 6-month differences in LVEF predict baseline to 24- 27 month differences in LVEF after Anthracycline-based therapy and concomitant atorvastatin therapy.

12.3. Analysis Plan

The primary hypothesis for Specific Aim 1 is that statin use will protect against a harmful reduction in LVEF measured over 24 – 27 months for patients with breast cancer or lymphoma who receive Anthracycline-based therapy. All randomized patients will be used in the analysis to compare groups ("intent to treat" approach). Since LVEF is measured on a continuous scale and this is a randomized trial, our primary analysis will fit an analysis of covariance (ANCOVA) model that compares the 24-27 month values of LVEF between groups adjusting for the baseline (pre-treatment) levels of LVEF and stratification factors. Note that patients are stratified by planned dose and that is the stratification that will be used in the analysis, even if the patient ends up getting a different dose. This analysis will address the first specific aim.

To address the second primary aim, we will examine whether the early treatment effect of statin use (6 months post randomization) predicts the long-term (24 – 27 month) effect of statins. Pilot data (Section 2) suggest that Anthracycline-based therapy injury occurs early and concomitant statin use attenuates this early Anthracycline-based therapy-mediated injury. We will fit an ANCOVA model that compares the 24- 27 month LVEF values for the treated and control groups and will also include covariates for the baseline LVEF and 6-month change in LVEF values. We will examine the treatment by 6-month LVEF change interaction to determine whether the correlation between early and late changes in LVEF differs by treatment. Thus, this model will have 4 terms of interest: the main effect for treatment, the main effect for the change in LVEF after 6 months, the main effect for baseline pre-treatment LVEF, and the treatment by change in LVEF after 6-month interaction. If the interaction is non-significant, we will examine the model with the 3 main effects. Since there may be limited power to test this interaction, we will use a 0.15 threshold for testing significance of the

interaction term in the model.

After primary efficacy analyses, additional analyses of the primary hypotheses will be done to assess relationships between treatment and outcome. We will fit ANCOVA models that consider the 24-27 month LVEF measure as the outcome, the baseline LVEF measure as a covariate, and other patient-level characteristics (e.g. age, cancer type, stage, and race) in order to control for other baseline covariates possibly related to LVEF in the model. Interactions between the treatment and covariates will be examined to determine whether the treatment effect is consistent across subgroups (e.g., black/white race by treatment interaction). Finally, we will conduct a repeated measures ANCOVA model that incorporates the intermediate (6-month) LVEF measurement into the model. We anticipate examining different covariance structures. Past experience suggests that a compound symmetry structure is usually appropriate; we will also examine whether an unstructured covariance is needed. We will fit these models using a mixed models approach, where the individual patient will be treated as a random effect in the model and the treatment indicator as patient characteristics (and baseline LVEF) will be considered as fixed effects. A mixed models approach for modeling the longitudinal data will allow for appropriate inference to be made in the presence of missing data (i.e., patient drop-outs) if the missing data are missing at random (MAR). Below, we describe methods to address MAR assumptions. Diagnostics and residual plots will be reviewed to ensure that all assumptions are met. If not, transformations of the outcome data will be considered, where the order of the priority in choosing a transformation will be to satisfy the (1) linearity assumption, (2) homogeneity assumption (homoscedasticity), and (3) normality assumption.

12.4. Potential Analytic Concerns in Primary Efficacy Analyses

Lost to Follow-up: We designed this study to recruit 278 total patients so that 200 are available at the end of the study. Thus, we will need to consider how to analyze data from the 78 patients who may drop out. To examine whether the missing data are either missing completely at random or missing at random, we will compare the baseline characteristics of those who drop out and those who complete the trial. If there is no evidence of informative missing data, analyses will be done using the repeated measures ANCOVA with a mixed models approach (PROC MIXED in SAS). However, if there is evidence that the missing data are informative and treatment-related (e.g. if there are fewer dropouts in the statin group or if missingness is related to other medical outcomes that are strongly associated with the outcome of interest) and not MAR, then we will use more sophisticated statistical methods.[78-80] One possible approach is to model the probability of dropping-out for each participant conditional on their baseline characteristics and treatment assignment. These probabilities would then be used to stratify participants (both completers and drop-outs) before making treatment comparisons. Thus each stratum would contain participants with similar predicted probabilities of trial completion (although only a subset would be completers). The final treatment effect estimates could be made using the completers with weights assigned to them based on the ratio of drop-outs to completers within each stratum. This approach and other methods for handling missing data have been by previously described by Dr. D'Agostino.[78-80]

12.5. Secondary Analyses: Once the primary efficacy analysis is completed, secondary efficacy variables will be examined to provide insight into mechanisms by which statins may influence LVEF. These include: LVESV and strain (reflective of LV contractility), LVEDV (reflective of LV pre-load), PWV and aortic wall thickness (reflective of LV after-load), and LVECV (a measure of myocellular replacement fibrosis). In addition, we will measure a series of subclinical biomarkers, including serum lipids (LDL, TC, HDL), glucose, serum troponin I, CRP, IL-6, TNF-α, renin, and aldosterone at the 6- and 24-27 month endpoints. For each, we will fit ANCOVA models that will examine the 24-27 month endpoints adjusted for baseline assessments, followed by ANCOVA models
that also include patient-level characteristics. Next, we will fit repeated measures ANCOVA models that incorporate intermediate (6-month) time point assessments. This approach will be repeated for the cognitive and patient reported outcomes. The latter analyses will also include data for the 12 and 18 months follow-ups.

12.6. Power and Sample Size considerations for the primary hypothesis

For the primary hypothesis, sample size and power calculations can be made based on direct comparisons between the expected changes in outcome means in the different treatment groups. These calculations need to account for the proportion of the variance in the primary efficacy of outcome (LVEF) explained by the other terms in the model (i.e., baseline values). To adjust for the proportion of variance explained by the baseline measurement when estimating the variance for the follow-up outcome measure, we used the formula [$V_{\text{follow-up adjusted for baseline covariates}} = V_{\text{follow-up}}(1-r^2)$], where r^2 is the square of the correlation between the baseline and follow-up measure. Our primary efficacy analysis is based on comparing the change in LVEF levels from baseline to 24 – 27 months, and r above is the correlation between these two measurements. These power calculations may be conservative, since intermediate assessments of LVEF at 6 months are not included. In other words, the mixed models approach (described in the additional analyses) would likely provide greater power than the ANCOVA model used here, but further assumptions concerning the covariance structure to be used and correlation among intermediate measures would need to be made. Thus, we believe that the power described by the simpler method (ANCOVA) is adequate to address our hypotheses and is expected to be conservative.

The following formula was used to describe the minimum detectable difference for the LVEF between the statin and placebo groups:

$$\text{Detectable difference} = \sigma \frac{\sqrt{2(1-r^2)(Z_{1-\alpha/2}+Z_{1-\beta})^2}}{\sqrt{n}}$$

In the above, r^2 is the percent of the variance of the follow-up outcome explained by the baseline measurement (LVEF), $Z_{1-\alpha/2}$ is the value from the standard normal distribution corresponding to the alpha level chosen (1.96, which corresponds to alpha=0.05 [two sided]), $Z_{1-\beta}$ is the value from the standard normal distribution that corresponds to the power chosen for the study (here 80% and 90%), and n=100 corresponds to the total number of patients studied for the statin group. We chose this sample size (100 per group) for power calculations to allow for up to 78 patients out of the 278 accrued to potentially drop-out. Note that this will somewhat conservative for the mixed models as those with partial data will also provide information. Based on preliminary data examining LVEF levels in comparable patients measured over a 6-month period we found that the standard deviation was approximately 7.7 and the correlation between baseline and 6-months was 0.5. Since we do not know what the correlation will be after 2 years, Table 5 depicts detectable differences for different correlations and power values. In addition, we show detectable differences for key secondary variables for which we have preliminary data (LVEDV, LVESV, and PWV).

Thus, for example, we have 80% power to detect a difference of 2.6% in LVEF between groups if the correlation between the baseline and 24-month LVEF is 0.5. In preliminary observational data, we saw a difference in LVEF between statin users vs. those who were not of 2.5% at 6 months. Thus, effect sizes of the magnitude in Table 5 are plausible and clinically meaningful. 2-6 ml increases in LVESV, or 3-5% drops in LVEF (from a normal resting LVEF) have been associated with adverse CV prognoses in a recent large trial of patients with HF and a preserved resting LVEF.[81] In 483 hypertensive patients aged 50±13 years, aortic PWV measures of 11 m/s were associated with odds ratio of 2.14 for cardiac events (p<0.0001).[82] Other studies that have initiated generic therapies such as ACE inhibitors or beta blockers with antioxidant properties have shown a reduced occurrence of CV events in cancer survivors.[83] Importantly however, it remains to be seen whether early initiation of statin therapy reduces the onset of subclinical CV disease in patients with breast cancer or lymphoma.

Table 5: Detectable Differences for Different Power and Correlation Values							
Outcome	Estimated SD	80% Power			90% Power		
		Correlation between baseline and 2-yr measure					
		0.4	0.5	0.6	0.4	0.5	0.6
LVEF (%)	7.7	2.79	2.64	2.44	3.23	3.06	2.82
LV-EDV (ml)	33.1	12.0	11.4	10.5	13.9	13.1	12.1
LV-ESV (ml)	19.7	7.1	6.8	6.2	8.3	7.8	7.2
PWV (m/sec)	3.7	1.34	1.27	1.2	1.6	1.5	1.4

12.7. Reporting and Exclusions

We will measure the level of compliance in randomized patients. If a patient drops out of the study early due to an adverse event that may be related to statin use, they will be non-compliant for subsequent treatment, and thus still inform the primary endpoint for our analyses, and their outcome will be considered as “non-compliant”. For patients who drop out early due to a non-statin related adverse event or die during the study we will analyze them in the following ways.

First we will conservatively assume that all drop-outs/deaths are non-compliant. Since there is a placebo group in this study we can assume that the rates would be similar between groups and still allow a comparison of compliance rates between groups. Next, we will consider two forms of imputation for secondary analyses. The first will use early compliance data (if available) to determine a compliance rate and that value will be carried forward to the end (i.e., Last observation carried forward) and the second would be to use a multiple imputation procedure to impute final compliance rates for participants based on modeling the compliance rates for those with complete data.

12.8. Evaluation of Toxicity

All participants will be evaluable for toxicity from the time of their first dose of atorvastatin.

12.9. Evaluation of Response

All participants will be assessed for compliance once they are randomized to treatment and receive at least one dose of treatment. The primary analysis will consist of all randomized patients who receive at least one dose of study drug. Secondary analyses will consist of patients who either complete the trial (with ≥ 75% compliance) or who drop out for adverse events that are related to the use of statins. Patients who drop out for non-related adverse events or die for reasons not related to statin use will be removed from the secondary analysis. Adverse events described by the Data Safety Monitoring Committee as related to statin use will be differentiated so as to render decisions regarding study drug compliance.

12.10. Interim Analysis

Interim reviews will be performed by the Data Safety Monitoring Committee every six months to assess accrual, retention, compliance, the incidence of AEs, SAEs, diabetes and LV function measures.

References

1. Brenner H. Long-term survival rates of cancer patients achieved by the end of the 20th century: a period analysis. *Lancet* 2002;360(9340):1131-5.
2. Coughlin SS, Ekwueme DU. Breast cancer as a global health concern. *Cancer Epidemiol* 2009;33(5):315-8.
3. Du XL, Fox EE,Lai D. Competing causes of death for women with breast cancer and change over time from 1975 to 2003. *Am J Clin Oncol* 2008;31(2):105-16.
4. Hooning MJ, Botma A, Aleman BM, Baaijens MH, Bartelink H, Klijn JG, Taylor CW,van Leeuwen FE. Long-term risk of cardiovascular disease in 10-year survivors of breast cancer. *J Natl Cancer Inst* 2007;99(5):365-75.
5. Maurea N, Coppola C, Ragone G, Frasci G, Bonelli A, Romano C,Iaffaioli RV. Women survive breast cancer but fall victim to heart failure: the shadows and lights of targeted therapy. *J Cardiovasc Med (Hagerstown)* 2010;11(12):861-8.
6. Schultz PN, Beck ML, Stava C,Vassilopoulou-Sellin R. Health profiles in 5836 long-term cancer survivors. *Int J Cancer* 2003;104(4):488-95.
7. Sukel MP, Breekveldt-Postma NS, Erkens JA, van der Linden PD, Beiderbeck AB, Coebergh JW,Herings RM. Incidence of cardiovascular events in breast cancer patients receiving chemotherapy in clinical practice. *Pharmacoepidemiol Drug Saf* 2008;17(2):125-34.
8. Feola M, Garrone O, Occelli M, Francini A, Biggi A, Visconti G, Albrile F, Bobbio M,Merlano M. Cardiotoxicity after anthracycline chemotherapy in breast carcinoma: effects on left ventricular ejection fraction, troponin I and brain natriuretic peptide. *Int J Cardiol* 2011;148(2):194-8.
9. Harbeck N, Ewer MS, De Laurentiis M, Suter TM,Ewer SM. Cardiovascular complications of conventional and targeted adjuvant breast cancer therapy. *Ann Oncol* 2011;22(6):1250-8.
10. Unverferth BJ, Magorien RD, Balcerzak SP, Leier CV,Unverferth DV. Early changes in human myocardial nuclei after doxorubicin. *Cancer* 1983;52(2):215-21.
11. Zambelli A, Della Porta MG, Eleuteri E, De Giuli L, Catalano O, Tondini C,Riccardi A. Predicting and preventing cardiotoxicity in the era of breast cancer targeted therapies. Novel molecular tools for clinical issues. *Breast* 2011;20(2):176-83.
12. Marty M, Espie M, Llombart A, Monnier A, Rapoport BL,Stahalova V. Multicenter randomized phase III study of the cardioprotective effect of dexrazoxane (Cardioxane) in advanced/metastatic breast cancer patients treated with anthracycline-based adjuvant therapy. *Ann Oncol* 2006;17(4):614-22.
13. Wiseman LR, Spencer CM. Dexrazoxane. A review of its use as a cardioprotective agent in patients receiving anthracycline-based adjuvant therapy. *Drugs* 1998;56(3):385-403.
14. Riad A, Bien S, Westermann D, Becher PM, Loya K, Landmesser U, Kroemer HK, Schultheiss HP,Tschope C. Pretreatment with statin attenuates the cardiotoxicity of Doxorubicin in mice. *Cancer Res* 2009;69(2):695-9.
15. Ramasubbu K, Estep J, White DL, Deswal A,Mann DL. Experimental and clinical basis for the use of statins in patients with ischemic and nonischemic cardiomyopathy. *J Am Coll Cardiol* 2008;51(4):415-26.
16. Zhou Q, Liao JK. Statins and cardiovascular diseases: from cholesterol lowering to pleiotropy. *Curr Pharm Des* 2009;15(5):467-78.
17. Acar Z, Kale A, Turgut M, Demircan S, Durna K, Demir S, Meric M,Agac MT. Efficiency of atorvastatin in the protection of anthracycline-induced cardiomyopathy. *J Am Coll Cardiol* 2011;58(9):988-9.
18. Ahern TP, Pedersen L, Tarp M, Cronin-Fenton DP, Garne JP, Silliman RA, Sorensen HT,Lash TL. Statin prescriptions and breast cancer recurrence risk: a Danish nationwide prospective cohort study. *J Natl Cancer Inst* 2011;103(19):1461-8.
19. Chaosuwannakit N, D'Agostino R, Jr., Hamilton CA, Lane KS, Ntim WO, Lawrence J, Melin SA, Ellis LR, Torti FM, Little WC,Hundley WG. Aortic stiffness increases upon receipt of anthracycline chemotherapy. *J Clin Oncol* 2010;28(1):166-72.
20. Twomley KM, D'Agostino Jr., R., Lawrence, J., et al. Low dose anthracycline-based adjuvant therapy and subclinical cardiovascular disease. *Circulation* 2011.

21. Pinder MC, Duan Z, Goodwin JS, Hortobagyi GN,Giordano SH. Congestive heart failure in older women treated with adjuvant anthracycline chemotherapy for breast cancer. *J Clin Oncol* 2007;25(25):3808-15.
22. Cardinale D, Colombo A, Lamantia G, Colombo N, Civelli M, De Giacomi G, Rubino M, Veglia F, Fiorentini C,Cipolla CM. Anthracycline-induced cardiomyopathy: clinical relevance and response to pharmacologic therapy. *J Am Coll Cardiol* 2010;55(3):213-20.
23. Patnaik JL, Byers T, DiGuiseppi C, Dabelea D,Denberg TD. Cardiovascular disease competes with breast cancer as the leading cause of death for older females diagnosed with breast cancer: a retrospective cohort study. *Breast Cancer Res* 2011;13(3):R64.
24. Bowles E, et al.: The Pharmacovigilance Research Group. Increased heart failure risk following anthracycline and trastuzumab treatment in a population-based cohort of women with invasive breast cancer. *Povisionally accepted at JNCI* 2012.
25. Geiger AM, Fischberg GM, Chen W,Bernstein L. Stroke risk and tamoxifen therapy for breast cancer. *J Natl Cancer Inst* 2004;96(20):1528-36.
26. Davies C, Godwin, J. (Early Breast Cancer Trialists Collaborative Group [EBCTCG]). Effects of chemotherapy and hormonal therapy for early breast cancer on recurrence and 15-year survival: an overview of the randomised trials. *Lancet* 2005;365(9472):1687-717.
27. Hortobagyi GN. Treatment of breast cancer. *N Engl J Med* 1998;339(14):974-84.
28. Valdivieso M, Burgess MA, Ewer MS, Mackay B, Wallace S, Benjamin RS, Ali MK, Bodey GP,Freireich EJ. Increased therapeutic index of weekly doxorubicin in the therapy of non-small cell lung cancer: a prospective, randomized study. *J Clin Oncol* 1984;2(3):207-14.
29. Slamon D, Eiermann W, Robert N, Pienkowski T, Martin M, Press M, Mackey J, Glaspy J, Chan A, Pawlicki M, Pinter T, Valero V, Liu MC, Sauter G, von Minckwitz G, Visco F, Bee V, Buyse M, Bendahmane B, Tabah-Fisch I, Lindsay MA, Riva A,Crown J. Adjuvant trastuzumab in HER2-positive breast cancer. *N Engl J Med* 2011;365(14):1273-83.
30. Di Leo A, Desmedt C, Bartlett JM, Piette F, Ejlertsen B, Pritchard KI, Larsimont D, Poole C, Isola J, Earl H, Mouridsen H, O'Malley FP, Cardoso F, Tanner M, Munro A, Twelves CJ, Sotiriou C, Shepherd L, Cameron D, Piccart MJ,Buyse M. HER2 and TOP2A as predictive markers for anthracycline-containing chemotherapy regimens as adjuvant treatment of breast cancer: a meta-analysis of individual patient data. *Lancet Oncol* 2011;12(12):1134-42.
31. Scully RE, Lipshultz SE. Anthracycline cardiotoxicity in long-term survivors of childhood cancer. *Cardiovasc Toxicol* 2007;7(2):122-8.
32. Mukhopadhyay P, Rajesh M, Batkai S, Kashiwaya Y, Hasko G, Liaudet L, Szabo C,Pacher P. Role of superoxide, nitric oxide, and peroxynitrite in doxorubicin-induced cell death in vivo and in vitro. *Am J Physiol Heart Circ Physiol* 2009;296(5):H1466-83.
33. Smith LA, Cornelius VR, Plummer CJ, Levitt G, Verrill M, Canney P,Jones A. Cardiotoxicity of anthracycline agents for the treatment of cancer: systematic review and meta-analysis of randomised controlled trials. *BMC Cancer* 2010;10:337.
34. Steinherz LJ, Steinherz PG, Tan CT, Heller G,Murphy ML. Cardiac toxicity 4 to 20 years after completing anthracycline therapy. *JAMA* 1991;266(12):1672-7.
35. Lipshultz SE, Scully RE, Lipsitz SR, Sallan SE, Silverman LB, Miller TL, Barry EV, Asselin BL, Athale U, Clavell LA, Larsen E, Moghrabi A, Samson Y, Michon B, Schorin MA, Cohen HJ, Neuberg DS, Orav EJ,Colan SD. Assessment of dexrazoxane as a cardioprotectant in doxorubicin-treated children with high-risk acute lymphoblastic leukaemia: long-term follow-up of a prospective, randomised, multicentre trial. *Lancet Oncol* 2010;11(10):950-61.
36. Swain SM, Vici P. The current and future role of dexrazoxane as a cardioprotectant in anthracycline treatment: expert panel review. *J Cancer Res Clin Oncol* 2004;130(1):1-7.
37. Swain SM, Whaley FS, Gerber MC, Weisberg S, York M, Spicer D, Jones SE, Wadler S, Desai A, Vogel C, Speyer J, Mittelman A, Reddy S, Pendergrass K, Velez-Garcia E, Ewer MS, Bianchine JR,Gams RA. Cardioprotection with dexrazoxane for doxorubicin-containing therapy in advanced breast cancer. *J Clin Oncol* 1997;15(4):1318-32.
38. Adler S, Huang H, Trochu JN, Xu X, Gupta S,Hintze TH. Simvastatin reverses impaired regulation of renal oxygen consumption in congestive heart failure. *Am J Physiol Renal Physiol* 2001;281(5):F802-9.

39. Delbosc S, Cristol JP, Descomps B, Mimran A,Jover B. Simvastatin prevents angiotensin II-induced cardiac alteration and oxidative stress. *Hypertension* 2002;40(2):142-7.
40. Gao L, Wang W,Zucker IH. Simvastatin inhibits central sympathetic outflow in heart failure by a nitric-oxide synthase mechanism. *J Pharmacol Exp Ther* 2008;326(1):278-85.
41. Degreef LE, Opdam FL, Teepe-Twiss IM, Jukema JW, Guchelaar HJ,Tamsma JT. The tolerability and efficacy of low-dose simvastatin in statin-intolerant patients. *Eur J Intern Med* 2010;21(4):293-6.
42. Damrot J, Nubel T, Epe B, Roos WP, Kaina B,Fritz G. Lovastatin protects human endothelial cells from the genotoxic and cytotoxic effects of the anticancer drugs doxorubicin and etoposide. *Br J Pharmacol* 2006;149(8):988-97.
43. Kotamraju S, Williams CL,Kalyanaraman B. Statin-induced breast cancer cell death: role of inducible nitric oxide and arginase-dependent pathways. *Cancer Res* 2007;67(15):7386-94.
44. Stephens NG, Parsons A, Schofield PM, Kelly F, Cheeseman K,Mitchinson MJ. Randomised controlled trial of vitamin E in patients with coronary disease: Cambridge Heart Antioxidant Study (CHAOS). *Lancet* 1996;347(9004):781-6.
45. Vivekananthan DP, Penn MS, Sapp SK, Hsu A,Topol EJ. Use of antioxidant vitamins for the prevention of cardiovascular disease: meta-analysis of randomised trials. *Lancet* 2003;361(9374):2017-23.
46. Albini A, Pennesi G, Donatelli F, Cammarota R, De Flora S,Noonan DM. Cardiotoxicity of anticancer drugs: the need for cardio-oncology and cardio-oncological prevention. *J Natl Cancer Inst* 2010;102(1):14-25.
47. Twomley KM, D'Agostino, R. Jr., Lawrence, J., Melin, S.A., Ellis, L.R., STacey, R.B., Hamilton, C.A., Torti, F.M., Little, W.C., Hundley, W.G. Early and persistent evidence of subclinical cardiovascular injury after receipt of anthracycline chemotherapy. *Circulation* 2010;122(21):Supplement:A12766.
48. Lightfoot JC, D'Agostino RB, Jr., Hamilton CA, Jordan J, Torti FM, Kock ND, Workman S,Hundley WG. Novel approach to early detection of doxorubicin cardiotoxicity by gadolinium-enhanced cardiovascular magnetic resonance imaging in an experimental model. *Circ Cardiovasc Imaging* 2010;3(5):550-8.
49. Jordan JH, D'Agostino, R.B. Jr., Hamilton, C.A., Hundley, W.G. Early increase in myocardial cardiovascular magnetic resonance gadolinium signal intensity after anthracyclines but not other agents used to treat breast cancer or hematologic malignancy. *J Am Coll Cardiol* 2011;57:E782.
50. Flett AS, Hayward MP, Ashworth MT, Hansen MS, Taylor AM, Elliott PM, McGregor C,Moon JC. Equilibrium contrast cardiovascular magnetic resonance for the measurement of diffuse myocardial fibrosis: preliminary validation in humans. *Circulation* 2010;122(2):138-44.
51. Chotenimitkhun R DAJR, Lawrence JA, Hamilton CA, Jordan J, Vasu S, Lash TL, Hundley WG. Preservation of left ventricular ejection fraction with statins during receipt of anthracycline-based adjuvant therapy. *J Am Coll Cardiol* 2012;59:E986.
52. Hundley WG, Kitzman DW, Morgan TM, Hamilton CA, Darty SN, Stewart KP, Herrington DM, Link KM,Little WC. Cardiac cycle-dependent changes in aortic area and distensibility are reduced in older patients with isolated diastolic heart failure and correlate with exercise intolerance. *J Am Coll Cardiol* 2001;38(3):796-802.
53. Herrington DM, Brown WV, Mosca L, Davis W, Eggleston B, Hundley WG,Raines J. Relationship between arterial stiffness and subclinical aortic atherosclerosis. *Circulation* 2004;110(4):432-7.
54. Rerkpattanapipat P, D'Agostino RB, Jr., Link KM, Shahar E, Lima JA, Bluemke DA, Sinha S, Herrington DM,Hundley WG. Location of arterial stiffening differs in those with impaired fasting glucose versus diabetes: implications for left ventricular hypertrophy from the Multi-Ethnic Study of Atherosclerosis. *Diabetes* 2009;58(4):946-53.
55. Puntawangkoon C, Morgan TM, Herrington DM, Hamilton CA,Hundley WG. Submaximal exercise coronary artery flow increases in postmenopausal women without coronary artery disease after estrogen and atorvastatin. *Menopause* 2010;17(1):114-20.
56. Yeh ET, Tong AT, Lenihan DJ, Yusuf SW, Swafford J, Champion C, Durand JB, Gibbs H, Zafarmand AA,Ewer MS. Cardiovascular complications of cancer therapy: diagnosis, pathogenesis, and management. *Circulation* 2004;109(25):3122-31.
57. Chu AY, Guilianini F, Barratt BJ, Nyberg F, Chasman DI,Ridker PM. Pharmacogenetic determinants of statin-induced reductions in C-reactive protein. *Circ Cardiovasc Genet* 2012;5(1):58-65.
58. Krum H, Ashton E, Reid C, Kalff V, Rogers J, Amarena J, Singh B,Tonkin A. Double-blind, randomized, placebo-controlled study of high-dose HMG CoA reductase inhibitor therapy on ventricular remodeling,

pro-inflammatory cytokines and neurohormonal parameters in patients with chronic systolic heart failure. *J Card Fail* 2007;13(1):1-7.
59. Chow SL, O'Barr SA, Peng J, Chew E, Pak F, Quist R, Patel P, Patterson JH,Heywood JT. Modulation of novel cardiorenal and inflammatory biomarkers by intravenous nitroglycerin and nesiritide in acute decompensated heart failure: an exploratory study. *Circ Heart Fail* 2011;4(4):450-5.
60. Weir RA, Tsorlalis IK, Steedman T, Dargie HJ, Fraser R, McMurray JJ,Connell JM. Aldosterone and cortisol predict medium-term left ventricular remodelling following myocardial infarction. *Eur J Heart Fail* 2011;13(12):1305-13.
61. Chahal H, Johnson C, Tandri H, Jain A, Hundley WG, Barr RG, Kawut SM, Lima JA,Bluemke DA. Relation of cardiovascular risk factors to right ventricular structure and function as determined by magnetic resonance imaging (results from the multi-ethnic study of atherosclerosis). *Am J Cardiol* 2010;106(1):110-6.
62. Heckbert SR, Post W, Pearson GD, Arnett DK, Gomes AS, Jerosch-Herold M, Hundley WG, Lima JA,Bluemke DA. Traditional cardiovascular risk factors in relation to left ventricular mass, volume, and systolic function by cardiac magnetic resonance imaging: the Multiethnic Study of Atherosclerosis. *J Am Coll Cardiol* 2006;48(11):2285-92.
63. Edvardsen T, Rosen BD, Pan L, Jerosch-Herold M, Lai S, Hundley WG, Sinha S, Kronmal RA, Bluemke DA,Lima JA. Regional diastolic dysfunction in individuals with left ventricular hypertrophy measured by tagged magnetic resonance imaging--the Multi-Ethnic Study of Atherosclerosis (MESA). *Am Heart J* 2006;151(1):109-14.
64. Hundley WG, Bluemke DA, Finn JP, Flamm SD, Fogel MA, Friedrich MG, Ho VB, Jerosch-Herold M, Kramer CM, Manning WJ, Patel M, Pohost GM, Stillman AE, White RD,Woodard PK. ACCF/ACR/AHA/NASCI/SCMR 2010 expert consensus document on cardiovascular magnetic resonance: a report of the American College of Cardiology Foundation Task Force on Expert Consensus Documents. *Circulation* 2010;121(22):2462-508.
65. Messroghli DR, Nordmeyer S, Dietrich T, Dirsch O, Kaschina E, Savvatis K, D Oh-I, Klein C, Berger F,Kuehne T. Assessment of diffuse myocardial fibrosis in rats using small-animal Look-Locker inversion recovery T1 mapping. *Circ Cardiovasc Imaging* 2011;4(6):636-40.
66. Gai N, Turkbey EB, Nazarian S, van der Geest RJ, Liu CY, Lima JA,Bluemke DA. T1 mapping of the gadolinium-enhanced myocardium: adjustment for factors affecting interpatient comparison. *Magn Reson Med* 2011;65(5):1407-15.
67. Redheuil A, Yu WC, Mousseaux E, Harouni AA, Kachenoura N, Wu CO, Bluemke D,Lima JA. Age-related changes in aortic arch geometry: relationship with proximal aortic function and left ventricular mass and remodeling. *J Am Coll Cardiol* 2011;58(12):1262-70.
68. Apple FS, Smith SW, Pearce LA, Ler R,Murakami MM. Use of the Centaur TnI-Ultra assay for detection of myocardial infarction and adverse events in patients presenting with symptoms suggestive of acute coronary syndrome. *Clin Chem* 2008;54(4):723-8.
69. Kusnierz-Cabala B, Gernand W, Zabek-Adamska A, Tokarz A,Naskalski JW. Comparison of high-sensitivity C-reactive protein serum assay results obtained using Dade-Behring BNII nephelometer and Ortho Vitros FS 5.1 clinical analyzer in respect of CRP-related risk assessment of chronic metabolic diseases. *Clin Lab* 2008;54(9-10):341-6.
70. Hudzik B, Szkodzinski J, Romanowski W, Danikiewicz A, Wilczek K, Lekston A, Polonski L,Zubelewicz-Szkodzinska B. Serum interleukin-6 concentration reflects the extent of asymptomatic left ventricular dysfunction and predicts progression to heart failure in patients with stable coronary artery disease. *Cytokine* 2011;54(3):266-71.
71. Navarro-Gonzalez JF, Mora C, Muros M, Jarque A, Herrera H,Garcia J. Association of tumor necrosis factor-alpha with early target organ damage in newly diagnosed patients with essential hypertension. *J Hypertens* 2008;26(11):2168-75.
72. Kerstens MN, Kobold AC, Volmer M, Koerts J, Sluiter WJ,Dullaart RP. Reference values for aldosterone-renin ratios in normotensive individuals and effect of changes in dietary sodium consumption. *Clin Chem* 2011;57(11):1607-11.
73. Brennan ML, Wu W, Fu X, Shen Z, Song W, Frost H, Vadseth C, Narine L, Lenkiewicz E, Borchers MT, Lusis AJ, Lee JJ, Lee NA, Abu-Soud HM, Ischiropoulos H,Hazen SL. A tale of two controversies: defining both the role of peroxidases in nitrotyrosine formation in vivo using eosinophil peroxidase and

myeloperoxidase-deficient mice, and the nature of peroxidase-generated reactive nitrogen species. *J Biol Chem* 2002;277(20):17415-27.
74. Sorensen HT, Riis AH, Lash TL,Pedersen L. Statin use and risk of amyotrophic lateral sclerosis and other motor neuron disorders. *Circ Cardiovasc Qual Outcomes* 2010;3(4):413-7.
75. Shaw EG, Rosdhal R, D'Agostino RB, Jr., Lovato J, Naughton MJ, Robbins ME,Rapp SR. Phase II study of donepezil in irradiated brain tumor patients: effect on cognitive function, mood, and quality of life. *J Clin Oncol* 2006;24(9):1415-20.
76. Sattar N, Preiss D, Murray HM, Welsh P, Buckley BM, de Craen AJ, Seshasai SR, McMurray JJ, Freeman DJ, Jukema JW, Macfarlane PW, Packard CJ, Stott DJ, Westendorp RG, Shepherd J, Davis BR, Pressel SL, Marchioli R, Marfisi RM, Maggioni AP, Tavazzi L, Tognoni G, Kjekshus J, Pedersen TR, Cook TJ, Gotto AM, Clearfield MB, Downs JR, Nakamura H, Ohashi Y, Mizuno K, Ray KK,Ford I. Statins and risk of incident diabetes: a collaborative meta-analysis of randomised statin trials. *Lancet* 2010;375(9716):735-42.
77. Therasse P, Arbuck SG, Eisenhauer EA, Wanders J, Kaplan RS, Rubinstein L, Verweij J, Van Glabbeke M, van Oosterom AT, Christian MC,Gwyther SG. New guidelines to evaluate the response to treatment in solid tumors. European Organization for Research and Treatment of Cancer, National Cancer Institute of the United States, National Cancer Institute of Canada. *J Natl Cancer Inst* 2000;92(3):205-16.
78. D'Agostino RB, Jr. Propensity score methods for bias reduction in the comparison of a treatment to a non-randomized control group. *Stat Med* 1998;17(19):2265-81.
79. D'Agostino RB, Jr., Rubin, D.B. Estimating and using propensity scores with partially missing data. *J Am Stat Assn* 2000;95(451):749-59.
80. Lavori PW, Dawson R,Shera D. A multiple imputation strategy for clinical trials with truncation of patient data. *Stat Med* 1995;14(17):1913-25.
81. Yusuf S, Teo KK, Pogue J, Dyal L, Copland I, Schumacher H, Dagenais G, Sleight P,Anderson C. Telmisartan, ramipril, or both in patients at high risk for vascular events. *N Engl J Med* 2008;358(15):1547-59.
82. Laurent S, Boutouyrie P, Asmar R, Gautier I, Laloux B, Guize L, Ducimetiere P,Benetos A. Aortic stiffness is an independent predictor of all-cause and cardiovascular mortality in hypertensive patients. *Hypertension* 2001;37(5):1236-41.
83. Cardinale D, Colombo A, Sandri MT, Lamantia G, Colombo N, Civelli M, Martinelli G, Veglia F, Fiorentini C,Cipolla CM. Prevention of high-dose chemotherapy-induced cardiotoxicity in high-risk patients by angiotensin-converting enzyme inhibition. *Circulation* 2006;114(23):2474-81.
84. Wagner LI SJ, Butt Z, et al. Trajectory of cognitive impairment during breast cancer treatment: a prospective analysis. *Journal of Clinical Oncology (Meeting Abstracts)* 2006;24(18suppl):8500.
85. Wefel JS, Lenzi R, Theriault R, Buzdar AU, Cruickshank S,Meyers CA. 'Chemobrain' in breast carcinoma?: a prologue. *Cancer* 2004;101(3):466-75.
86. O'Brien JT. Vascular cognitive impairment. *Am J Geriatr Psychiatry* 2006;14(9):724-33.
87. Stampfer MJ. Cardiovascular disease and Alzheimer's disease: common links. *J Intern Med* 2006;260(3):211-23.
88. Wendell CR, Zonderman AB, Metter EJ, Najjar SS,Waldstein SR. Carotid intimal medial thickness predicts cognitive decline among adults without clinical vascular disease. *Stroke* 2009;40(10):3180-5.
89. Elias MF, Sullivan LM, Elias PK, D'Agostino RB, Sr., Wolf PA, Seshadri S, Au R, Benjamin EJ,Vasan RS. Left ventricular mass, blood pressure, and lowered cognitive performance in the Framingham offspring. *Hypertension* 2007;49(3):439-45.
90. Unverzagt FW, McClure LA, Wadley VG, Jenny NS, Go RC, Cushman M, Kissela BM, Kelley BJ, Kennedy R, Moy CS, Howard V,Howard G. Vascular risk factors and cognitive impairment in a stroke-free cohort. *Neurology* 2011;77(19):1729-36.
91. Marcus DS, Olsen TR, Ramaratnam M,Buckner RL. The Extensible Neuroimaging Archive Toolkit: an informatics platform for managing, exploring, and sharing neuroimaging data. *Neuroinformatics* 2007;5(1):11-34.
92. Michielutte R, Beal P. Identification of community leadership in the development of public health education programs. *J Community Health* 1990;15(1):59-68.

93. Minasian LM, Carpenter WR, Weiner BJ, Anderson DE, McCaskill-Stevens W, Nelson S, Whitman C, Kelaghan J, O'Mara AM,Kaluzny AD. Translating research into evidence-based practice: the National Cancer Institute Community Clinical Oncology Program. *Cancer* 2010;116(19):4440-9.
94. Hortobagyi GN. Treatment of breast cancer. *N Eng J Med*, 1998;339:974-84.
95. Valdivieso M, Burgess MA, Dwer MS, Mackay B, Wallace S, Benjamin RS, Ali MK, Bodey GP, Freireich EJ. Increased therapeutic index of weekly Doxorubicin in the therapy of non-small cell lung cancer: a prospective, randomized study. *J Clin Onc* 1984;2:207-14.

96. Torti FM, Aston D, Lum BL, Kohler M, Williams R, Spaulding JT, Shortliffe L, Freiha FS. Weekly Doxorubicin in endocrine-refractory carcinoma of the prostate. *J Clin Oncol* 1983;8:477-482.

97. Lipshultz SE, Colan SD, Gelber RD, Perez-Atayde AR, Sallan SE, Sanders SP. Late cardiac effects of Doxorubicin therapy for acute lymphoblastic leukemia in childhood. *N Engl J Med* 1991;324:808-15.

98. LaRosa JC, Grundy SM, Waters DD, Shear C, Barter P, Fruchart JC, Gotto AM, Greten H, Kastelein JJ, Shepherd J, Wenger NK, Treating to New Targets (TNT) Investigators. Intensive lipid lowering with atorvastating in patients with stable coronary disease. N Engl J Med. 2005 Apr 7;352(14):1425-35. PMID: 15755765.

.
