# Supplementary material for: Preserved Cognitive Function After Statin Administration During Cancer Treatment With Doxorubicin: A Secondary Analysis of a Randomized Clinical Trial
Source: JAMA Netw Open. 2025 Oct 21;8(10):e2538325. doi: 10.1001/jamanetworkopen.2025.38325 (PMC12541534; doi:10.1001/jamanetworkopen.2025.38325)
Supplement: Supplement 2. — eFigure 1. Flowchart of Included Study Participants eTable 1. Baseline Characteristics of Those With and Without Cognitive Outcome Data in the Overall Population eTable 2. Baseline Characteristics of Those With and Without Cognitive Outcome Data in the Analyzed Population eTable 3. Cognitive Assessment Scores Across All Three Visits by Group: Anthracycline Based Chemotherapy and Placebo or Anthracycline Based Chemotherapy and Statin eTable 4. Part A Trail Making Test (TMT-A), Time in Seconds to Complete eTable 5. Part A Trail Making Test (TMT-A), Number of Errors eFigure 2. Part A Trail Making Test (TMT-A), Number of Errors eFigure 3. Estimated Part B Trail Making Test (TMT-B) Errors eTable 6. Part B Trail Making Test (TMT-B), Time in Seconds to Complete eTable 7. Unadjusted Within-Group Changes From Baseline to 24 Months on Cognitive Assessment Scores Across All Measures eTable 8. Part B Trail Making Test (TMT-B), Number of Errors eTable 9. COWA-Estimated Frequency (Words) [file jamanetwopen-e2538325-s002.pdf]

Supplemental Online Content

Grizzard PJ, O'Connell NS, Rapp SR, et al. Preserved cognitive function after statin administration during cancer treatment with doxorubicin: a secondary analysis of a randomized clinical trial. *JAMA Netw Open*. 2025;8(10):e2538325. doi:10.1001/jamanetworkopen.2025.38325

eFigure 1. Flowchart of Included Study Participants
eTable 1. Baseline Characteristics of Those With and Without Cognitive Outcome Data in the Overall Population
eTable 2. Baseline Characteristics of Those With and Without Cognitive Outcome Data in the Analyzed Population
eTable 3. Cognitive Assessment Scores Across All Three Visits by Group: Anthracycline Based Chemotherapy and Placebo or Anthracycline Based Chemotherapy and Statin
eTable 4. Part A Trail Making Test (TMT-A), Time in Seconds to Complete
eTable 5. Part A Trail Making Test (TMT-A), Number of Errors
eFigure 2. Part A Trail Making Test (TMT-A), Number of Errors
eFigure 3. Estimated Part B Trail Making Test (TMT-B) Errors
eTable 6. Part B Trail Making Test (TMT-B), Time in Seconds to Complete
eTable 7. Unadjusted Within-Group Changes From Baseline to 24 Months on Cognitive Assessment Scores Across All Measures
eTable 8. Part B Trail Making Test (TMT-B), Number of Errors
eTable 9. COWA-Estimated Frequency (Words)

This supplemental material has been provided by the authors to give readers additional information about their work.

© 2025 Grizzard PJ et al. *JAMA Network Open.*

eFigure 1. Flowchart of Included Study Participants

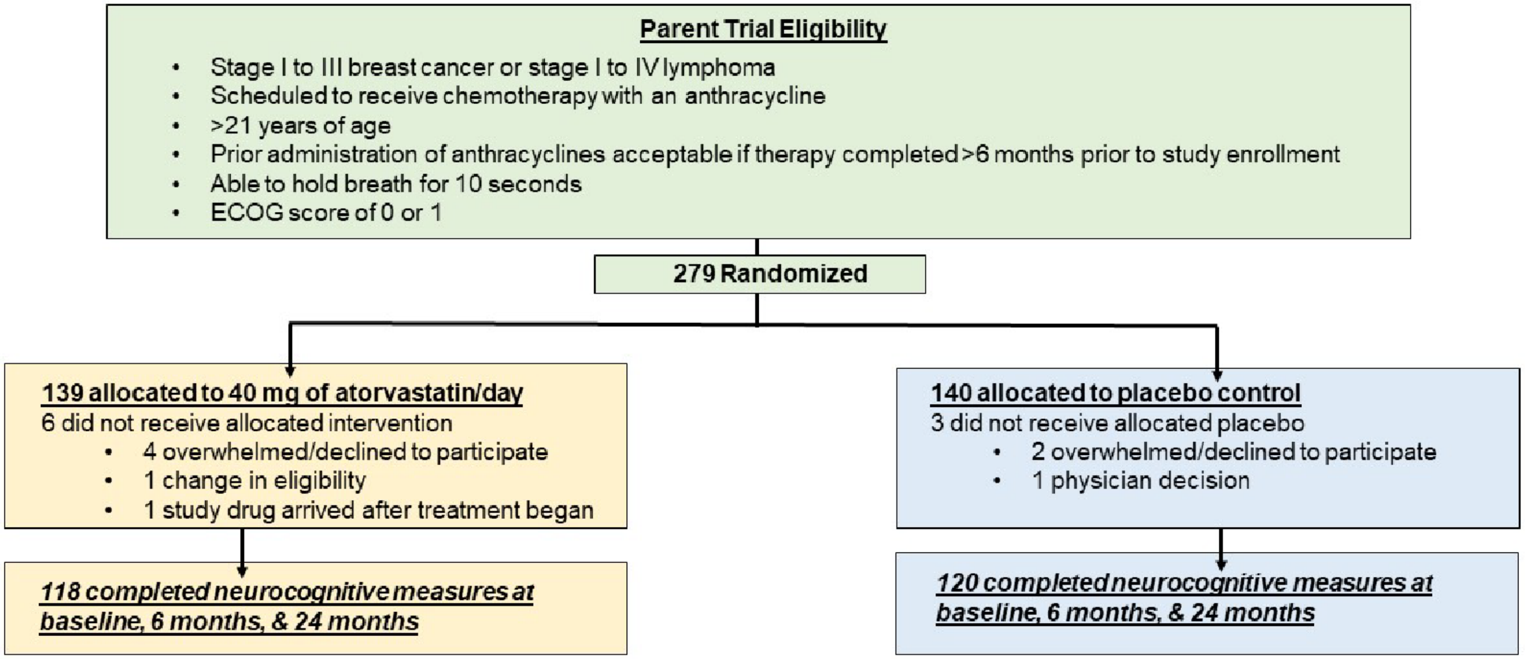

Figure S1: Flow Chart of Included Study Participants in this Secondary Analysis

© 2025 Grizzard PJ et al. *JAMA Network Open.*

eTable 1. Baseline Characteristics of Those With and Without Cognitive Outcome Data in Overall Population

Mean (± SD)			
	**Does not have 24 Month Cognition Data (N=80)**	**Has 24 Month Cognition Data (N=199)**	**Overall (N=279)**
**Treatment Group**			
Placebo	37 (46.3%)	103 (51.8%)	140 (50.2%)
Statin	43 (53.8%)	96 (48.2%)	139 (49.8%)
**Height (cm)**	165 (6.77)	166 (8.28)	166 (7.90)
**Weight (kg)**	82.0 (20.3)	82.9 (19.9)	82.6 (20.0)
**Body-mass Index**	30.2 (7.45)	29.9 (6.83)	30.0 (7.00)
**Age**	46.2 (12.2)	50.1 (11.7)	49.0 (12.0)
**Sex**			
Female	77 (96.3%)	179 (89.9%)	256 (91.8%)
Male	3 (3.8%)	20 (10.1%)	23 (8.2%)
**Race**			
Black	14 (17.5%)	24 (12.1%)	38 (13.6%)
Other*	5 (6.3%)	5 (2.5%)	10 (3.6%)
White	61 (76.3%)	170 (85.4%)	231 (82.8%)
**Cancer Type**			
Breast	70 (87.5%)	168 (84.4%)	238 (85.3%)
Lymphoma	10 (12.5%)	31 (15.6%)	41 (14.7%)
**Cancer Stage**			
1	22 (27.5%)	24 (12.1%)	46 (16.5%)
2	34 (42.5%)	120 (60.3%)	154 (55.2%)
3	24 (30.0%)	50 (25.1%)	74 (26.5%)
4	0 (0%)	5 (2.5%)	5 (1.8%)
**Marital Status**			
Married	39 (48.8%)	139 (69.8%)	178 (63.8%)
Other**	41 (51.3%)	60 (30.2%)	101 (36.2%)
**Education**			
College	44 (55.0%)	113 (56.8%)	157 (56.3%)

© 2025 Grizzard PJ et al. *JAMA Network Open.*

Graduate or Professional	12 (15.0%)	48 (24.1%)	60 (21.5%)
High School	24 (30.0%)	38 (19.1%)	62 (22.2%)
**Income**			
<$35k	23 (28.8%)	51 (25.6%)	74 (26.5%)
$35k-$75k	24 (30.0%)	60 (30.2%)	84 (30.1%)
>$75k	33 (41.3%)	88 (44.2%)	121 (43.4%)
**Job Status**			
Disabled	5 (6.3%)	9 (4.5%)	14 (5.0%)
Employed	44 (55.0%)	134 (67.3%)	178 (63.8%)
Other***	24 (30.0%)	34 (17.1%)	58 (20.8%)
Retired	7 (8.8%)	22 (11.1%)	29 (10.4%)
**Ethnicity**			
Hispanic/Latino	3 (3.8%)	5 (2.5%)	8 (2.9%)
Not Hispanic/Latino	77 (96.3%)	193 (97.0%)	270 (96.8%)
Unknown	0 (0%)	1 (0.5%)	1 (0.4%)
**TMT-A, time (s)†**	29.8 (13.3)	30.2 (14.1)	30.1 (13.9)
Missing	7 (8.8%)	0 (0%)	7 (2.5%)
**TMT-B, time (s)†**	69.5 (41.1)	71.8 (40.7)	71.2 (40.7)
Missing	12 (15.0%)	11 (5.5%)	23 (8.2%)
**TMTB - TMTA**	39.7 (37.1)	41.3 (33.4)	40.8 (34.4)
Missing	12 (15.0%)	11 (5.5%)	23 (8.2%)
**TMT-A Errors**	0.229 (0.487)	0.271 (0.566)	0.260 (0.546)
Missing	10 (12.5%)	0 (0%)	10 (3.6%)
**TMT-B Errors**	0.462 (0.920)	0.511 (0.978)	0.498 (0.962)
Missing	15 (18.8%)	11 (5.5%)	26 (9.3%)
**COWA**	38.6 (10.4)	38.9 (11.5)	38.8 (11.2)
Missing	10 (12.5%)	0 (0%)	10 (3.6%)

*Other races include Asian, Native Hawaiian/Pacific Islander, Native American/Alaskan, and Unknown.
**Other marital statuses include Single - never married, Living in a married like relationship, Separated or divorced, Widowed, and Prefer not to answer.
***Other job statuses include Not working, Homemaker, and Unknown.
† Time in seconds (s)

© 2025 Grizzard PJ et al. *JAMA Network Open.*

eTable 2. Baseline Characteristics of Those With and Without Cognitive Outcome Data in Analyzed Population Mean (± SD)

	Does not have 24 Month Cognition Data (N=50)	Has 24 Month Cognition Data (N=188)	Overall (N=238)
**Treatment Group**			
Placebo	24 (48.0%)	96 (51.1%)	120 (50.4%)
Statin	26 (52.0%)	92 (48.9 %)	118 (49.6%)
**Height (cm)**	165 (7.19)	166 (8.24	166 (8.02)
**Weight (kg)**	81.6 (21.8)	82.9 (20.0)	82.6 (20.4)
**Body-mass Index**	29.8 (7.99)	29.9 (6.88)	29.9 (7.11)
**Age**	46.1 (12.8)	49.9 (11.5)	49.1 (11.9)
**Sex**			
Female	47 (94.0%)	170 (90.4%)	217 (91.2%)
Male	3 (6.0%)	18 (9.6%)	21 (8.8%)
**Race**			
Black	8 (16.0%)	22 (11.7%)	30 (12.6%)
Other*	3 (6.0%)	4 (2.1%)	7 (2.9%)
White	39 (78.0%)	162 (86.2%)	201 (84.5%)
**Cancer Type**			
Breast	43 (86.0%)	159 (84.6%)	202 (84.9%)
Lymphoma	7 (14.0%)	29 (15.4%)	36 (15.1%)
**Cancer Stage**			
1	14 (28.0%)	23 (12.2%)	37 (15.5%)
2	20 (40.0%)	113 (60.1%)	133 (55.9%)
3	16 (32.0%)	47 (25.0%)	63 (26.5%)
4	0 (0%)	5 (2.7%)	5 (2.1%)
**Marital Status**			
Married	24 (48.0%)	132 (70.2%)	156 (65.5%)
Other**	26 (52.0%)	56 (29.8%)	82 (34.5%)
**Education**			
College	30 (60.0%)	108 (57.4%)	138 (58.0%)

© 2025 Grizzard PJ et al. *JAMA Network Open.*

Graduate or Professional	10 (20.0%)	43 (22.9%)	53 (22.3%)
High School	10 (20.0%)	37 (19.7%)	47 (19.7%)
**Income**			
<$35k	15 (30.0%)	47 (25.0%)	62 (26.1%)
$35k-$75k	18 (36.0%)	58 (30.9%)	76 (31.9%)
>$75k	17 (34.0%)	83 (44.1%)	100 (42.0%)
**Job Status**			
Disabled	0 (0%)	9 (4.8%)	9 (3.8%)
Employed	29 (58.0%)	130 (69.1%)	159 (66.8%)
Other***	16 (32.0%)	29 (15.4%)	45 (18.9%)
Retired	5 (10.0%)	20 (10.6%)	25 (10.5%)
**Ethnicity**			
Hispanic/Latino	0 (0%)	5 (2.7%)	5 (2.1%)
Not Hispanic/Latino	50 (100%)	182 (96.8%)	232 (97.5%)
Unknown	0 (0%)	1 (0.5%)	1 (0.4%)
**TMT-A, time (s)†**	29.7 (12.2)	30.2 (13.8)	30.1 (13.5)
Missing	0 (0%)	0 (0%)	0 (0%)
**TMT-B, time (s)†**	74.6 (52.6)	70.8 (37.7)	71.6 (41.0)
Missing	4 (8.0%)	8 (4.3%)	12 (5.0%)
**TMTB - TMTA**	44.5 (45.0)	40.4 (31.5)	41.3 (34.6)
Missing	4 (8.0%)	8 (4.3%)	12 (5.0%)
**TMT-A Errors**	0.204 (0.499)	0.287 (0.578)	0.270 (0.563)
Missing	1 (2.0%)	0 (0%)	1 (0.4%)
**TMT-B Errors**	0.333 (0.929)	0.528 (0.994)	0.489 (0.982)
Missing	5 (10.0%)	8 (4.3%)	13 (5.5%)
**COWA**	38.9 (12.0)	38.9 (11.4)	38.9 (11.5)
Missing	1 (2.0%)	0 (0%)	1 (0.4%)

*Other races include Asian, Native Hawaiian/Pacific Islander, Native American/Alaskan, and Unknown.
**Other marital statuses include Single - never married, Living in a married like relationship, Separated or divorced, Widowed, and Prefer not to answer.
***Other job statuses include Not working, Homemaker, and Unknown.
† Time in seconds (s)

© 2025 Grizzard PJ et al. *JAMA Network Open.*

eTable 3. Cognitive Assessment Scores Across All Three Visits by Group: Anthracycline Based Chemotherapy and Placebo or Anthracycline Based Chemotherapy and Statin

	Mean (±SD)					
	Baseline		6 Months		24 Months	
	Placebo (N=120)	Statin (N=118)	Placebo (N=120)	Statin (N=118)	Placebo (N=120)	Statin (N=118)
**TMT-A, time (s)***	28.1 (11.0)	32.1 (15.4)	26.9 (11.8)	31.1 (21.0)	26.8 (10.2)	29.3 (13.7)
Missing	0 (0%)	0 (0%)	1 (0.8%)	0 (0%)	18 (15.0%)	25 (21.2%)
**TMT-B, time (s)***	69.0 (39.3)	74.1 (42.7)	65.6 (35.3)	71.1 (40.2)	67.7 (39.7)	63.4 (30.7)
Missing	9 (7.5%)	3 (2.5%)	4 (3.3%)	2 (1.7%)	24 (20.0%)	26 (22.0%)
**TMTB -TMTA**	40.6 (34.6)	41.9 (34.7)	38.7 (29.1)	39.8 (34.4)	41.1 (33.0)	34.6 (22.8)
Missing	9 (7.5%)	3 (2.5%)	4 (3.3%)	2 (1.7%)	24 (20.0%)	26 (22.0%)
**TMT-A Errors**	0.24 (0.48)	0.31 (0.63)	0.23 (0.51)	0.25 (0.51)	0.28 (0.57)	0.28 (0.58)
Missing	1 (0.8%)	0 (0%)	5 (4.2%)	2 (1.7%)	18 (15.0%)	26 (22.0%)
**TMT-B Errors**	0.44 (0.94)	0.54 (1.02)	0.47 (0.93)	0.37 (0.74)	0.40 (0.76)	0.37 (0.75)
Missing	10 (8.3%)	3 (2.5%)	8 (6.7%)	4 (3.4%)	24 (20.0%)	26 (22.0%)
**COWA**	40.7 (11.8)	37.1 (10.8)	40.8 (11.5)	37.6 (11.1)	45.3 (11.9)	41.1 (12.4)
Missing	1 (0.8%)	0 (0%)	4 (3.3%)	2 (1.7%)	18 (15.0%)	26 (22.0%)

* Time in seconds (s)

© 2025 Grizzard PJ et al. *JAMA Network Open.*

eTable 4. Part A Trail Making Test (TMT-A), Time in Seconds to Complete

Predictors	*Estimates*	*CI*	*p*
(Intercept)	13.38	2.13 – 24.64	**0.02**
Visit: 6 M	-0.17	-3.44 – 3.11	0.92
Visit: 24 M	-0.79	-4.16 – 2.58	0.64
Statin group	4.22	0.26 – 8.19	**0.04**
Glucose levels	0.03	-0.03 – 0.09	0.30
Job Status: Employed	-1.86	-8.87– 5.16	0.60
Job Status: Other	3.88	-3.68 – 11.43	0.31
Job Status: Retired	-1.23	-9.39 – 6.94	0.77
Age	0.24	0.11 – 0.37	**<0.001**
Visit 6M: Statin group	-0.07	-4.64 – 4.50	0.98
Visit 24M: Statin group	-2.15	-6.97 – 2.66	0.38

© 2025 Grizzard PJ et al. *JAMA Network Open.*

eTable 5. Part A Trail Making Test (TMT-A), Number of Errors

Predictors	*Incidence Rate Ratios*	*CI*	*p*
(Intercept)	0.12	0.03 – 0.44	**0.001**
Visit: 6 M	0.93	0.55 – 1.59	0.80
Visit: 24 M	1.13	0.67 – 1.89	0.64
Statin group	1.17	0.69 – 1.98	0.57
Age	1.02	1.00 – 1.04	**0.02**
College Degree	0.5	0.32 – 0.77	**0.002**
Graduate/Professional Degree	0.77	0.47 – 1.28	0.31
Job status: Employed	0.92	0.39 – 2.16	0.84
Job status: Other	0.87	0.34 – 2.21	0.77
Job status: Retired	0.65	0.24 – 1.80	0.41
Visit 6M: Statin group	0.88	0.43 – 1.81	0.72
Visit 24M: Statin group	0.81	0.39 – 1.68	0.57

© 2025 Grizzard PJ et al. *JAMA Network Open.*

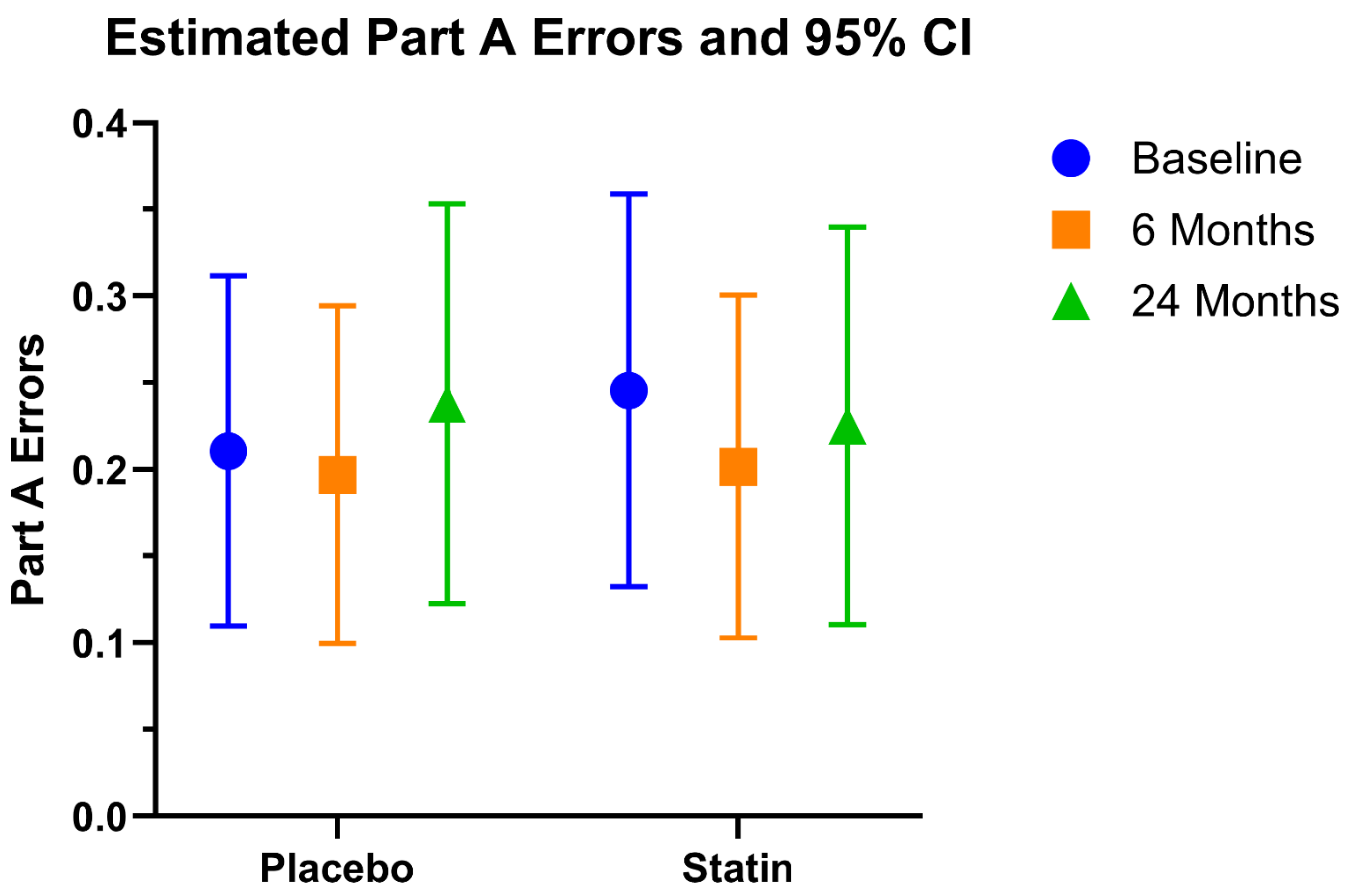

eFigure 2. Part A Trail Making Test (TMT-A), Number of Errors. Differences in number of errors on TMT-A are shown for the individuals (x-axis) who were randomly assigned to placebo (left) or statin (right). The baseline (circles), 6 months (squares), and 24 months (triangles) show the mean number of errors and 95% confidence interval across the y-axis estimated from the generalized linear mixed model shown in Table S5.

© 2025 Grizzard PJ et al. *JAMA Network Open.*

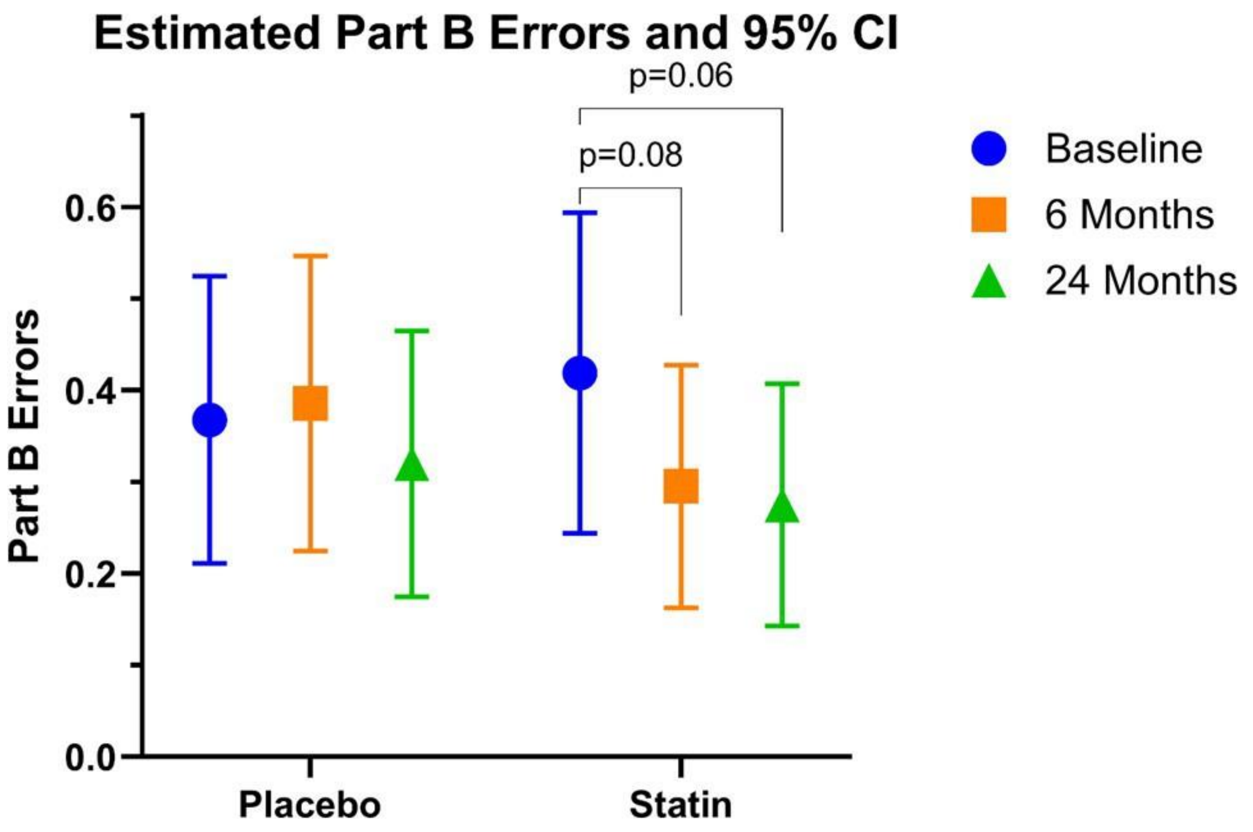

eFigure 3. Estimated Part B Trail Making Test (TMT-B) Errors. Differences in number of errors on TMT-B are shown for the individuals (x-axis) who were randomly assigned to placebo (left) or statin (right). The baseline (circles), 6 months (squares), and 24 months (triangles) show the mean number of errors and 95% confidence interval across the y-axis estimated from a generalized linear mixed model shown in Table S8.

© 2025 Grizzard PJ et al. *JAMA Network Open.*

eTable 6. Part B Trail Making Test (TMT-B), Time in Seconds to Complete

Predictors	*Estimates*	*CI*	*p*
(Intercept)	55.4	22.98 – 87.82	**0.001**
Visit: 6 M	-4.07	-12.03 – 3.89	0.32
Visit: 24 M	0.2	-8.09 – 8.48	0.96
grp: Statin	4.6	-5.82 – 15.01	0.39
Age	0.83	0.50 – 1.17	**<0.001**
College Degree	-19.87	-30.01 – -9.72	**<0.001**
Graduate/Professional Degree	-13.07	-25.91 – -0.22	**0.05**
Income: $35k-$75k	-6.58	-16.89 – 3.73	0.21
Income: >$75k	-9.89	-20.37 – 0.59	0.06
Race: Black	10.81	-1.16 – 22.78	0.08
Race: Other	37.28	14.18 – 60.39	**0.002**
Cancer type: Lymphoma	5.8	-5.31 – 16.91	0.31
Diastolic BP	-0.14	-0.37 – 0.09	0.23
HDL	-0.1	-0.31 – 0.11	0.34
Glucose	0.05	-0.10 – 0.20	0.53
Tumor necrosis factor-α	1.28	-1.26 – 3.81	0.32
Visit 6M: Statin group	1.15	-9.75 – 12.04	0.84
Visit 24M: Statin group	-10.4	-22.02 – 1.23	0.08

© 2025 Grizzard PJ et al. *JAMA Network Open.*

eTable 7. Unadjusted Within-Group Changes From Baseline to 24 Months on Cognitive Assessment Scores Across All Measures

	Part A Time Sec Total			Part B Time Sec Total			Part B minus A (time)		
Predictors	*Estimates*	*CI*	*p*	*Estimates*	*CI*	*p*	*Estimates*	*CI*	*p*
(Intercept)	28.05	25.48 – 30.62	**<0.001**	68.69	61.59 – 75.80	**<0.001**	40.58	34.66 – 46.50	**<0.001**
sqnb [6M]	-1.23	-4.10 – 1.65	0.402	-3.78	-9.97 – 2.41	0.231	-2.34	-7.91 – 3.24	0.411
sqnb [24M]	-1.44	-4.46 – 1.58	0.35	-2.24	-8.84 – 4.36	0.506	-0.22	-6.15 – 5.72	0.943
grp [Statin]	4.03	0.38 – 7.69	**0.03**	4.85	-5.17 – 14.86	0.342	1.02	-7.31 – 9.36	0.809
sqnb [6M] × grp [Statin]	0.27	-3.80 – 4.34	0.897	1.15	-7.55 – 9.84	0.795	0.44	-7.38 – 8.26	0.912
sqnb [24M] × grp [Statin]	-1.8	-6.15 – 2.54	0.416	-7.01	-16.35 – 2.34	0.142	-5.78	-14.18 – 2.63	0.178
Type III Test for Interaction			0.61			0.19			0.28
Within Group Changes at 24M (Baseline - 24M)									
Placebo	1.44	(-1.59, 4.46)	0.35	2.24	(-4.37, 8.85)	0.506	0.216	(-5.73, 6.16)	0.943
Statin	3.243	(0.11, 6.37)	0.042	9.247	(2.62, 15.87)	0.006	5.991	(0.03, 11.95)	0.049

© 2025 Grizzard PJ et al. *JAMA Network Open.*

eTable 7 (continued)

	Part A Errors			Part B Errors			COWA		
Predictors	*Incidence Rate Ratios*	*CI*	*p*	*Incidence Rate Ratios*	*CI*	*p*	Estimates	CI	p
(Intercept)	0.17	0.11 – 0.26	**<0.001**	0.27	0.19 – 0.39	**<0.001**	40.73	38.64 – 42.83	**<0.001**
sqnb [6M]	1.03	0.61 – 1.73	0.909	1.03	0.70 – 1.50	0.899	0.48	-0.96 – 1.93	0.51
sqnb [24M]	1.23	0.74 – 2.05	0.427	0.85	0.56 – 1.30	0.464	4.4	2.89 – 5.91	**<0.001**
grp [Statin]	1.33	0.80 – 2.21	0.277	1.14	0.72 – 1.80	0.578	-3.66	-6.63 – -0.69	**0.016**
sqnb [6M] × grp [Statin]	0.85	0.42 – 1.70	0.64	0.68	0.40 – 1.16	0.159	0.12	-1.92 – 2.16	0.906
sqnb [24M] × grp [Statin]	0.75	0.37 – 1.53	0.425	0.76	0.42 – 1.39	0.376	-0.3	-2.48 – 1.88	0.788
Type III Test for Interaction			0.72			0.35			0.93
Within Group Changes at 24M (Baseline - 24M)									
Placebo	0.039	(-0.138, 0.059)	0.437	0.04	(-0.066, 0.145)	0.46	-4.395	(-5.91, -2.88)	<0.001
Statin	0.018	(-0.089, 0.126)	0.74	0.108	(-0.066, 0.145)	0.043	-4.097	(-5.67, -2.53)	<0.001

© 2025 Grizzard PJ et al. *JAMA Network Open.*

eTable 8. Part B Trail Making Test (TMT-B), Number of Errors

Predictors	*Incidence Rate Ratios*	*CI*	*p*
(Intercept)	0.16	0.04 – 0.58	**0.005**
Visit: 6 M	1.05	0.71 – 1.56	0.81
Visit: 24 M	0.87	0.56 – 1.34	0.53
Statin group	1.14	0.71 – 1.84	0.59
Age	1.03	1.01 – 1.05	**0.001**
College Degree	0.52	0.32 – 0.82	**0.005**
Graduate/Professional Degree	0.79	0.46 – 1.36	0.4
Job Status: Employed	0.52	0.22 – 1.21	0.13
Job Status: Other	0.71	0.28 – 1.78	0.47
Job Status: Retired	0.63	0.24 – 1.70	0.37
Visit 6M: Statin group	0.67	0.39 – 1.16	0.16
Visit 24M: Statin group	0.75	0.41 – 1.38	0.36

© 2025 Grizzard PJ et al. *JAMA Network Open.*

eTable 9. COWA-Estimated Frequency (Words)

Predictors	*Estimates*	*CI*	*p*
(Intercept)	46.74	34.52 – 58.96	**<0.001**
Visit: 6 M	0.05	-1.82 – 1.92	0.96
Visit: 24 M	3.62	1.71 – 5.54	**<0.001**
Statin Group	-3.64	-6.81 – -0.47	**0.02**
Age	-0.09	-0.23 – 0.04	0.17
College Degree	7.27	3.69 – 10.85	**<0.001**
Graduate/Professional Degree	8.77	4.26 – 13.28	**<0.001**
Job Status: Employed	-4.74	-11.79 – 2.31	0.19
Job Status: Other	-4.17	-11.67 – 3.34	0.28
Job Status: Retired	-7.94	-15.99 – 0.10	0.05
Visit 6 M: Statin Group	1.15	-1.64 – 3.94	0.42
Visit 24 M: Statin Group	1.11	-1.65 – 3.88	0.43

© 2025 Grizzard PJ et al. *JAMA Network Open.*
