# Supplementary material for: Preserved Cognitive Function After Statin Administration During Cancer Treatment With Doxorubicin: A Secondary Analysis of a Randomized Clinical Trial
Source: JAMA Netw Open. 2025 Oct 21;8(10):e2538325. doi: 10.1001/jamanetworkopen.2025.38325 (PMC12541534; doi:10.1001/jamanetworkopen.2025.38325)
Supplement: Supplement 3. — Data Sharing Statement [file jamanetwopen-e2538325-s003.pdf]

Data Sharing Statement

Data
Additional Information: Preventing Anthracycline Cardiovascular Toxicity With Statins (PREVENT) https://clinicaltrials.gov/study/NCT01988571 ClinicalTrials.gov ID: NCT01988571
Data available: No
